# Enantioconvergent Synthesis of Diarylmethane Drugs via Privileged Benzhydrol Intermediates

**DOI:** 10.1021/acs.joc.5c02530

**Published:** 2025-12-26

**Authors:** Eduard Frank, Jana L. Flügel, Ludwig d’Heureuse, Sophie Woick, Alexander Breder

**Affiliations:** Institute for Organic Chemistry, 9147 University of Regensburg 93053 Regensburg, Germany

## Abstract

The benzhydryl motif is a privileged pharmacophore in antihistaminic and neuroactive drugs. We present a broadly applicable, enantioconvergent synthesis of benzhydrols via asymmetric migratory Tsuji-Wacker oxidation of stilbenes. This constitutionally stereodivergent protocol operates without preactivated or sterically biased substrates, affording chiral α,α-diaryl ketones in up to 91% *ee*, which convert to benzhydrols without erosion of stereochemistry. The method enables concise syntheses of (*S*)-cloperastine and isotopically labeled (*S*)-diphenhydramine-*d*_5_, establishing chiral benzhydrols as versatile intermediates for redox- and step-economic drug assembly.

## Graphical abstract

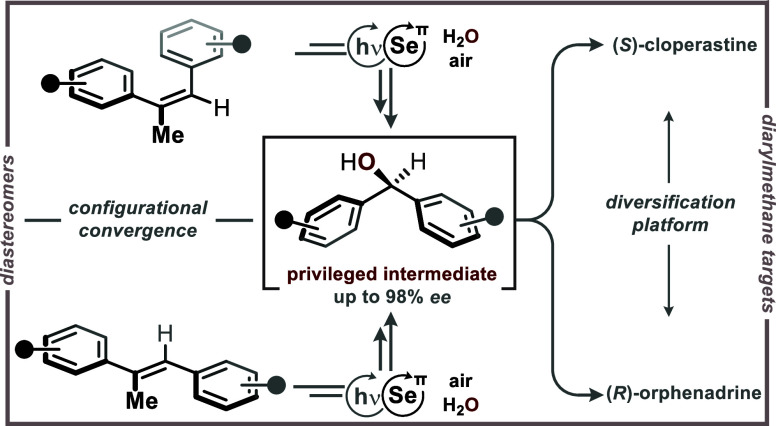

Diarylmethanes are a prevalent structure motif found in over 300 drug targets with broad pharmacological activities, including antihistaminic and analgesic effects among others (Figure 1a).^1,2^ For example, the widely used diphenhydramine, a Parkinson’s medication,^3^ combines antihistaminic and anticholinergic properties with sedative effects,^4^ inhibits neuronal Na^+^ channels^5^ and interacts with opioid receptors.^6^ While such multitarget activity expands therapeutic potential, it can also cause adverse effects, including intoxication.^7–10^ An early approach to enhance its specificity was the installation of methyl groups in the arene periphery, for example, realized in the design of orphenadrine by exchange of a phenyl group for an *o*-tolyl residue (i.e., *o*-methylation). Orphenadrine (Figure 1a, left) shows enhanced anticholinergic effects,^11^ and is employed as a muscle relaxant and analgesic, often in combination with paracetamol,^12^ while the discontinued neobenodine, featuring a *p*-tolyl group, has enhanced antihistaminic activity.^1,11^ Initially, most antihistamines were administered as racemic mixtures. Levocetirizine, the (*R*)-enantiomer of cetirizine (Figure 1a, center), was FDA-approved in 2007 after being identified as the eutomer (i.e., responsible for most antihistaminic activity) and shown to possess more favorable pharmacokinetics, including slower clearance.^13–15^ For many modern diarylmethane-based drugs, only the eutomer is used to enhance efficacy and minimize adverse effects. For example, escitalopram, the (*S*)-enantiomer of citalopram (Figure 1a, right), is a widely prescribed selective serotonin reuptake inhibitor (SSRI) for depression and anxiety.^16^ Escitalopram is twice as potent as racemic citalopram and 27 times more potent than the distomer (*R*)-citalopram,^17,18^ which is not only inactive for serotonin reuptake inhibition, but also antagonizes the activity of (*S*)-citalopram.^19^ These findings highlight the importance of enantioselective methods for the development of diarylmethane pharmaceuticals and, in particular, benzhydrols as their common precursors.

**Figure 1:**
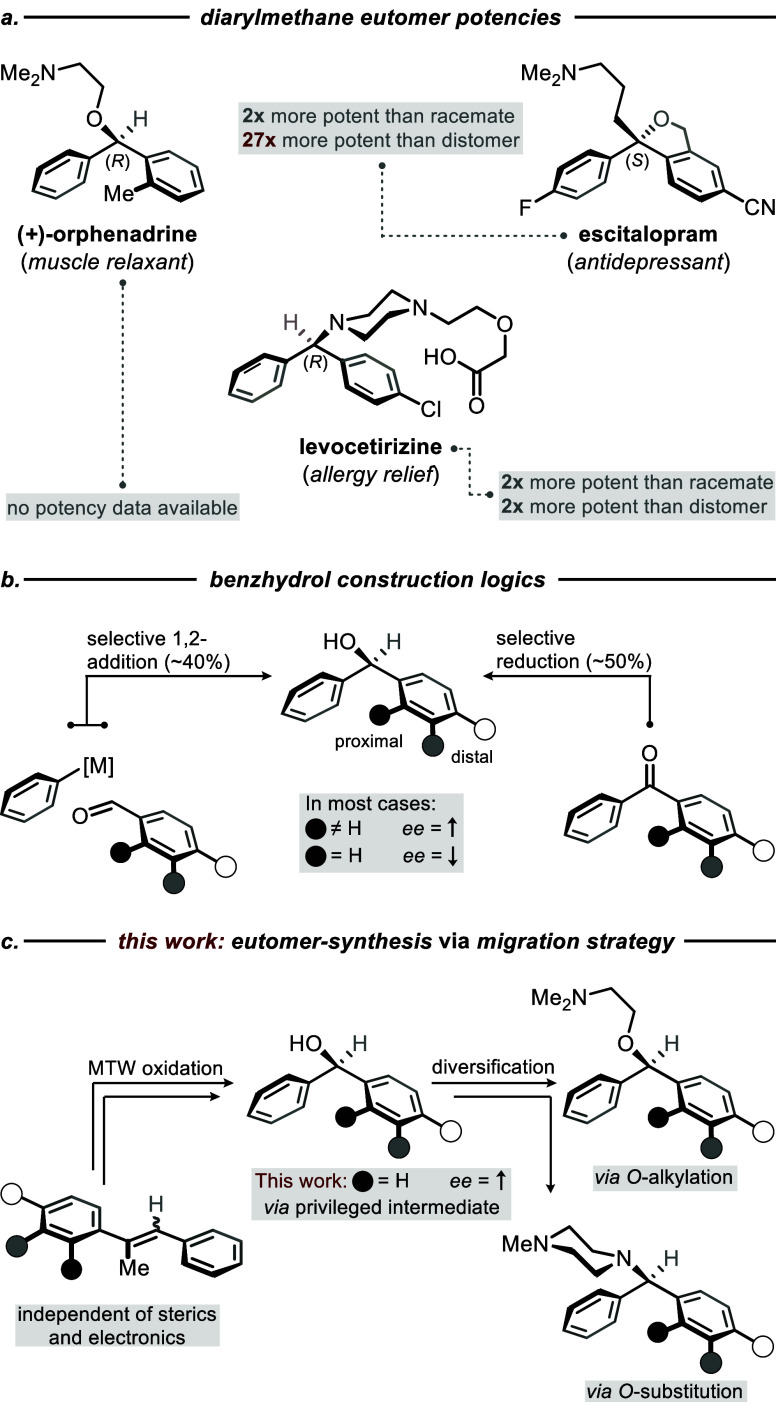
(a) Selected examples of diarylmethane-based pharmaceuticals and their potency data. (b) General construction logics for the assembly of chiral benzhydrols. (c) This work: eutomer-synthesis of diarylmethanes via asymmetric migratory Tsuji-Wacker oxidation of stilbenes.

Among the various synthetic approaches to benzhydrols,^20–22^ most asymmetric variants rely on two key strategies: (1) selective 1,2-addition of metalated arenes onto prochiral carbonyl groups as in benzaldehydes and phenones^23–27^ or (2) reduction of diarylketones (Figure 1b).^28–33^ Instructive examples for strategy (1) include the Rh-catalyzed addition of phenylboronic acid onto *p*-tolualdehyde by Arao et al. in their total synthesis of (*R*)-neobenodine which was completed in 76% overall yield and >99% *ee*.^23^ Key to their success was the implementation of a hemilabile chiral P-ligand on the Rh center, which ensured a rigid coordination sphere resulting in selective addition. While similar approaches also achieved high *ee* values, a common feature is the need for preactivated substrates, e.g., in the form of *p*-chlorinated^25^ or *o*-silylated^26^ arenes that require additional postmodification steps to complete the total syntheses. Along the same lines, enantioselective reduction of benzophenones (strategy 2) requires a chiral catalyst to efficiently distinguish between the two arene rings, either by means of sterics^28–31^ or electronics,^32,33^ which becomes increasingly difficult with the lack of *o*-substituents or strong electron-donating or electron-withdrawing groups. An elegant solution toward this problem was reported by Li et al. by exploitation of a selective Cr­(CO)_3_ arene coordination prior to asymmetric reduction to secure high *ee* values by temporarily increasing the sterics on one of the arene rings.^33^ Their idea was predicated on the stabilities of Cr­(CO)_3_ arene complexes, adapted from a report by Corey and Helal on their total synthesis of cetirizine.^34^ However, electronic discrimination and postmodification is still required, albeit to a minor extent.

Against this background, we became interested in the idea of accessing the privileged benzhydryl motif by means of an intramolecular rearrangement^35,36^ such as our previously established asymmetric migratory Tsuji-Wacker (MTW) oxidation of stilbenes (Figure 1c).^37^ While affording α,α-diaryl ketones in high enantioselectivities, our approach is completely independent of any steric and electronic factors within the arene rings. Furthermore, the protocol is also stereoconvergent with respect to the stilbene configuration, thus allowing to access a broad variety of benzhydrols in high *ee* in two steps from the α,α-diaryl ketone intermediate without installation or removal of directing groups. The privileged benzhydrol intermediates can be harnessed as expedient lynchpins toward various diarylmethane pharmacophores. Another feature that must be particulary emphasized from a conceptional point of view, is the methods ability to divergently access both enantiomers from constitutionally isomeric stilbenes, simply by relocating the stilbene’s methyl group. This subtle change allows us to ensure the selective assembly of the actual pharmacologically active enantiomer (i.e., the eutomer).

To demonstrate the potential of our method, we commenced with the syntheses of (*R*)-orphenadrine (**3**), its analogue **3′**, and (*R*)-naphthoneobenodine (**4**) (Scheme 1a). By applying the MTW oxidation to the respective stilbene precursors (Scheme S1, Supporting Information), we were able to obtain enantioenriched ketones **1a** and **1b** in 91% *ee* and 87% *ee*, respectively (Scheme 1a). A sequence of Baeyer–Villiger oxidation and basic hydrolysis^38^ with methanolic K_2_CO_3_ furnished the corresponding benzhydrols **2a** and **2b** with only minor erosion of stereoinformation (i.e., 87% *ee* and 77% *ee*), which could be enhanced by a simple recrystallization step to 98% *ee* and 90% *ee*, respectively. Williamson etherification^39^ completed the total syntheses of (*R*)-orphenadrine (**3**) and its cyclohexyl analogue **3′** with total yields of 24% and 18%, respectively, over six steps and *ee* values of 98% for both products. Similarly, for the first time, (*R*)-naphthoneobenodine (**4**) was obtained in 25% yield over six steps with an *ee* of 87%. These results clearly highlight the flexibility and expedience of our method. Simultaneously, they underscore the method’s independence from any steric biases in the arene units, which is of pertinence when considering a prospective implementation of such a protocol in the design of drug candidate libraries. Compared to established synthetic routes toward (*R*)-orphenadrine (**3**),^25,26,31^ our pathway ranks among the highest in terms of enantioselectivity without the need for postimplementation of the *o*-methyl group (Scheme 1b).

**Scheme 1:**
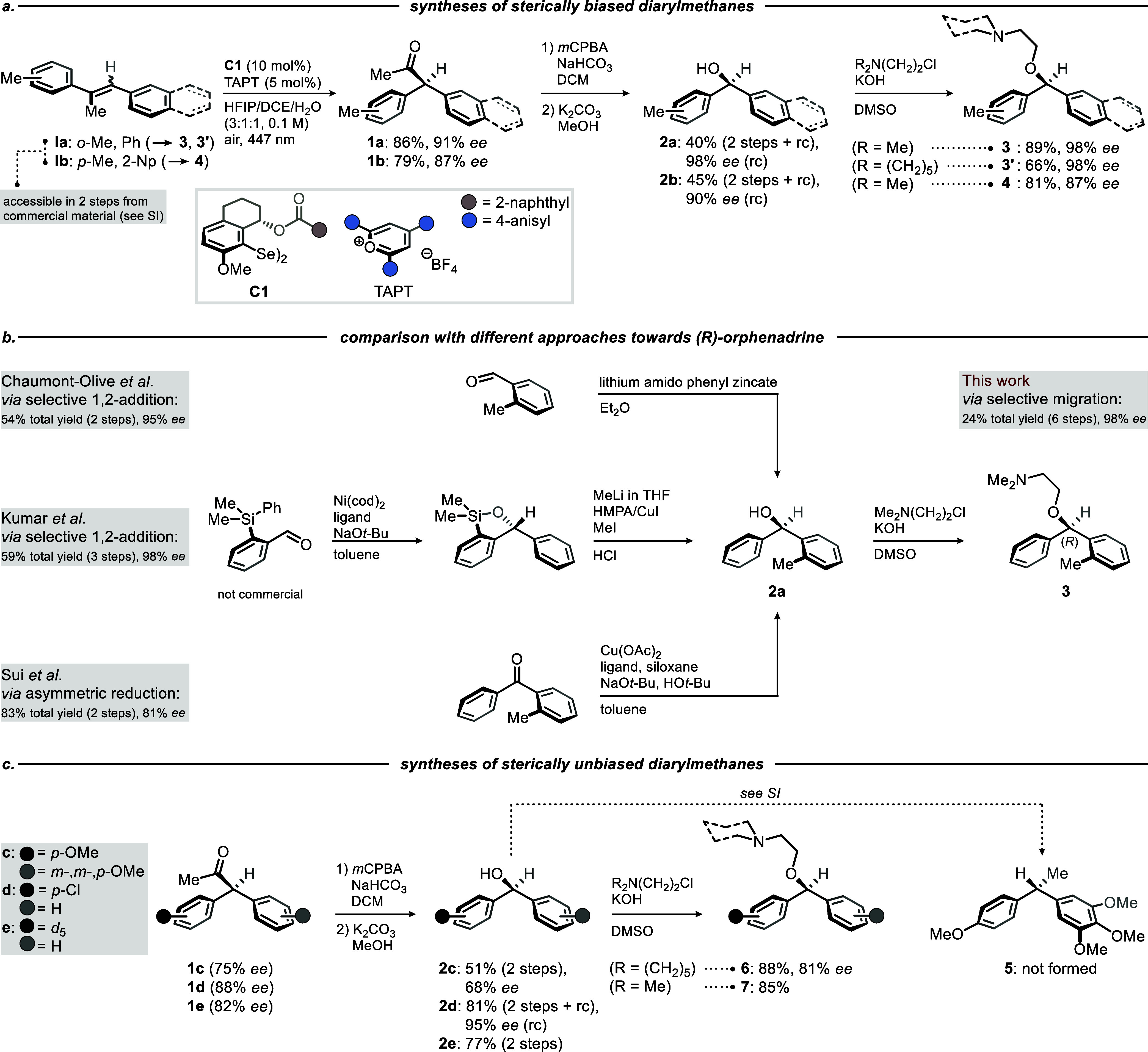
(a) Expedited Total Syntheses of Sterically Biased Diarylmethanes (i.e., Arene Groups Bearing *o*-Substituents) from Stilbenes **Ia** and **Ib**: The Latter Two Compounds Were Accessed in Two Steps from Commercial Precursors: (b) Comparison of Our (*R*)-Orphenadrine Synthesis with Different Approaches Available in Literature: (c) Challenging Total Syntheses of Sterically Unbiased Diarylmethanes (i.e., Arene Groups Bearing *m*- and *p*-Substituents): HFIP = 1,1,1,3,3,3-Hexafluoropropan-2-ol; DCE = 1,2-Dichloroethane; *m*CPBA = *meta*-Chloroperoxybenzoic Acid; HMPA = Hexamethylphosphorotriamide; rc = Recrystallization

Next, we turned our attention to diarylmethanes exhibiting *m*- and *p*-substitution patterns (i.e., sterically unbiased diarylmethanes), as those are more challenging to transform into target structures with reasonably high *ee* values (Scheme 1c). Electron-rich benzhydrol **2c** was obtained from ketone **1c** in a respectable total yield of 25% over five steps from commercial substrates with an *ee* of 68% (Scheme S1, Supporting Information). Attempts to transform this structure to the N1L protein antagonist **5** via various methylation strategies were unsuccessful (Table S1, Supporting Information). Nevertheless, enantioenriched **2c** is an inhibitor of tubulin polymerization and acts as a cytotoxic compound,^40^ thus leaving us with some therapeutic value.

Another isosteric benzhydrol was obtained in the form of **2d**, which could be converted to (*S*)-cloperastine (**6**) in a total yield of 15% over five steps from commercial precursors with 81% *ee* (Scheme S1, Supporting Information). While both (*S*)-cloperastine and its enantiomer possess antitussive activity (*S*)-cloperastine is associated with a more favorable profile, particularly concerning central nervous system effects.^41^ Following our previous synthesis of pentadeuterated isotopomer **1e**, whose *ee* was determined to be 82%,^37^ we transformed it into privileged (*S*)-benzhydrol **2e** and subsequently into the pentadeuterated analogue of (*S*)-diphendydramine (**7**). It showed an optical rotation of −9.5° leaving us with the assumption that the high *ee* value was conserved during the reaction sequence with a total yield of 47% over five steps (Scheme S1, Supporting Information), as was the case for all previously discussed targets. Such isotopomers gain increasing attention in pharmacokinetics,^42^ and, as the equivalent tritium-labeled compounds, may have potential in theranostics through various imaging techniques.^43^

Since all the previous examples share an oxygen atom attached to the central diarylmethane carbon atom, we focused on the substitution of the oxygen atom in the privileged intermediate instead of *O*-alkylation (Figure 2). Accordingly, we targeted chlorcyclizine (**8**), a first-generation antihistamine and antiemetic that is in use for the treatment of allergies and nausea, respectively. Commercial syntheses usually rely on racemic reduction of 4-chlorobenzophenone (strategy 2). Our approach focused on the stereospecific substitution of the oxygen residue in benzhydrol **2d** to access its chlorinated analogue (Scheme S2, Supporting Information).^44^ Unfortunately, chlorination with thionyl chloride eroded the *ee* value from 95% to only 16%, which is probably due to the doubly benzylic position tending to undergo S_N_1 type reactions upon activation of the hydroxyl residue as leaving group. However, subsequent amination with *N*-methylpiperazine furnished target compound **8** in 79% yield without any further erosion of the *ee* value (i.e., 16% *ee*). We believe the significant erosion of stereoinformation cannot be avoided by using this synthetic route. Nevertheless, our approach serves as a proof of principle for the enantioselective synthesis of isosteric benzhydrylamines like chlorcyclizine (**8**), which was afforded in a total yield of 12% after six steps and 16% *ee*. We suspect that analogues of **8**, such as the previously mentioned eutomer (*R*)-cetirizine (see Figure 1a, center), may also accessible following the same synthetic pathway.

**Figure 2:**
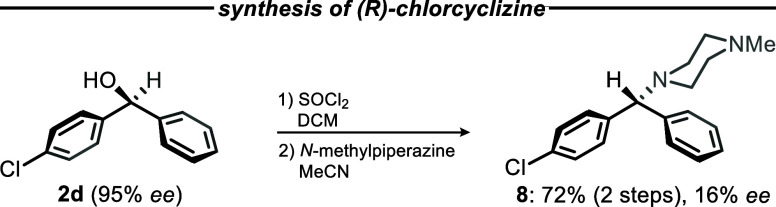
Synthesis of (*R*)-chlorcyclizine starting from the privileged benzhydrol intermediate.

In summary, we have developed a complementary and highly versatile synthetic route toward enantioenriched antihistaminic and neuroactive pharmaceuticals with up to 98% *ee*. By taking advantage of the benzhydrol as a privileged structural motif, which is easily accessible by our established asymmetric MTW oxidation, seven targets with the diphenhydramine or cetirizine scaffolds were synthesized in six or less consecutive steps from commercial precursors with an average total yield of 24% (Scheme S1, Supporting Information). As an additional benefit of our method compared to related work, the assembly of diarylmethanes is independent of any steric and electronic influences within the arene rings and does not require any late-stage modifications such as removal of directing groups or halide-alkyl exchanges. Further, we have demonstrated that the eutomer of the target structures can, in principle, be accessed from just one catalyst enantiomer, as was explicitly showcased for (*S*)-cloperastine (**6**) and chlorcyclizine (**8**).^41,45^ Altogether, our protocol provides access to structurally diverse benzhydrols, which serve as versatile lynchpins for the enantiocontrolled assembly of antihistaminic diarylmethane drugs.

## Supplementary Material



## Data Availability

The data underlying this study are openly available in Zenodo at 10.5281/zenodo.17160137.

## Notes

Experimental procedures, NMR spectra, IR spectra, and HPLC data for all newly synthesized compounds (PDF)
