# Supplementary material for: Enantioconvergent Synthesis of Diarylmethane Drugs via Privileged Benzhydrol Intermediates
Source: J Org Chem. 2025 Dec 26;91(1):793–7. doi: 10.1021/acs.joc.5c02530 (PMC12797289; doi:10.1021/acs.joc.5c02530)
Supplement: Supplementary file 1 [file jo5c02530_si_001.pdf]

Supporting Information for

Enantioconvergent Synthesis of Diarylmethane Drugs *via* Privileged Benzhydrol Intermediates

Eduard Frank,[‡] Jana L. Flügel,[‡] Ludwig d'Heureuse, Sophie Woick, and Alexander Breder*

Institute for Organic Chemistry, University of Regensburg, 93053 Regensburg, Germany

Corresponding author email: alexander.breder@ur.de

[‡]E.F. and J.L.F. contributed equally.

Table of Contents

1. General remarks......S3

2. Overview of antihistaminic and neuroactive diarylmethanes......S5

3. Preparation of photoredox and selenium catalysts S5

4. Synthesis of *O*-alkylated compounds **3**, **3'**, **4**, **6**, and **7** S6

5. Attempted synthesis of N1L protein antagonist **5**......S20

6. Synthesis of *O*-substituted antihistamine **8** S22

7. References S24

8. NMR and IR spectra......S26

9. HPLC data......S62

1. General remarks

Chemicals and solvents: All commercially available chemicals were purchased in high quality and used without further purification. Solvents for column chromatography were distilled prior to use. Moisture and oxygen-sensitive reactions were carried out using dry solvents from a MBraun Solvent Purification System (SPS) in flame-dried glassware under inert atmosphere of cobalt chloride-dried nitrogen. Current concentration of solutions containing organolithium compounds was determined via titration with *N*-(2-tolyl)formamide in dry THF (0.1 M). The evaporation of solvents was carried out in a rotary evaporator at 40 ºC, under reduced pressure. Room temperature (rt) was approximately 23 °C. Reactions at temperatures of 0 °C or below were conducted in a suitable freezing mixture (water/ice, acetone/dry ice) or a thermostat when run overnight. "Brine" refers to a saturated solution of sodium chloride in water. Irradiation setup: Irradiation experiments were performed using custom built temperature-controlled metal blocks and commercially available blue LED lights (Rebel LXML PR01 0500 Royal Blue) operating at a constant current (700 mA) with an intensity maximum in the range of λ_{max} = 447 nm to 465 nm and an output power of 414 mW to 433 mW. The reaction vessel was a standard 100 mL round-bottom flask made of borosilicate glass, which had a distance of 1 cm to the LED. No filters were used. For a detailed picture of the setup, please see the SI (Figure S1) of our previous publication.[1] Column chromatography (CC): Acros Silica 60 (0.035–0.70 mm, 70–230 mesh ASTM) was used as the stationary phase with appropriate solvent mixtures applying forced flow. Purification by automated flash column chromatography was performed on a Advion puriFlash® 5.050 machine using either pre-packed puriFlash® columns or Acros Silica 60 self-packed columns. Thin-layer chromatography (TLC): Reactions were monitored by TLC on silica gel pre-coated aluminium sheets (Machery-Nagel, silica gel 60 G/UV254, 0.2 mm). Visualization was accomplished by exposure to UV light (λ = 254 nm or 365 nm) and by dipping the plates in a *p*-anisaldehyde staining solution (composition: 270 mL EtOH, 7.4 mL *p*-anisaldehyde, 10 mL conc. H_2SO_4), a potassium permanganate staining solution (composition: 3 g potassium permanganate, 20 g potassium carbonate, 5 mL 5% aq. NaOH, 300 mL H_2O), or a cerium molybdate staining solution (composition: 12 g ammonium molybdate, 235 mL H_2O, 0.5 g ceric ammonium molybdate, 15 mL H_2SO_4 conc.) followed by heating. Nuclear magnetic resonance (NMR): NMR spectra were recorded at room temperature using a Bruker Avance 300 NMR spectrometer (300 MHz for ^{1}H, 75 MHz for ^{13}C{^{1}H}), a Bruker Avance 400 or Bruker Avance III HD 400 NMR spectrometer (400 MHz for ^{1}H, 101 MHz for ^{13}C{^{1}H}, 61 MHz for ^{2}H, and 162 MHz for ^{31}P{^{1}H}). Chemical shifts are reported in δ-scale in parts per million (ppm) and referenced to the residual proton signal of the used solvent: $CDCl_3$ (δ = 7.26 ppm, ^{1}H and ^{2}H; δ = 77.2 ppm, ^{13}C), $(CD_3)_2SO$ (δ = 2.50 ppm, ^{1}H; δ = 39.5 ppm, ^{13}C). ^{31}P{^{1}H} spectra are referenced to H_3PO_4. Coupling constants *J* are given in Hertz (Hz) and the multiplicities of the

signals are abbreviated as: s = singlet, d = doublet, t = triplet, q = quartet, p = pentet, m = multiplet (denotes complex pattern), br = broad signal, and combinations of those. Signals are reported as follows: (multiplicity, coupling constant *J*, number of protons). Isomeric ratios (*E*:*Z*) were determined by the ratio of ^{1}H NMR integrals of the isolated products. For ^{1}H NMR yield determination, the solvent of the reaction mixture was removed under reduced pressure after reaction completion. The residue was taken up in $CDCl_3$ (2.5 mL), and an internal standard was added. Infrared spectroscopy (IR): IR spectra were recorded on an Agilent Cary630 FTIR spectrophotometer with the neat substances and are reported in cm^{-1}. High resolution mass spectrometry (HRMS): Mass spectra were obtained from the central analytic mass spectrometry facilities of the Faculty of Chemistry and Pharmacy, University of Regensburg. All mass spectra were recorded on a Finnigan MAT 95, Thermo Quest Finnigan TSQ 7000, Finnigan MATSSQ 710 A or an Agilent Q-TOF 6540 UHD instrument. X-ray structure analysis: Structure determination of compounds by X-ray analysis was performed by the X-ray structure analysis department of the Faculty of Chemistry and Pharmacy, University of Regensburg. Data were collected on Rigaku Synergy DW, Mova / Ag or GV Cu-α / Cu-β single crystal diffractometers. High-performance liquid chromatography (HPLC): Enantiomeric ratios were determined by chiral HPLC measurements on an Agilent 1290 Infinity with the following columns provided by the company DAICEL: Chiralpak® IA-3 (4.6x250 mm, particle size 3 µm), Chiralpak® IB-3 (4.6x250 mm, particle size 3 µm), Chiralpak® IC-3 (4.6x250 mm, particle size 3 µm), Chiralpak® ID-3 (4.6x250 mm, particle size 3 µm), Chiralcel® OD-3 (4.6x250 mm, particle size 3 µm), Chiralcel® OJ-3 (4.6x250 mm, particle size 3 µm). Melting points (m.p.): Melting points were measured on a KRÜSS Opticron melting point meter M5000 without further correction of the values. Optical rotation ($[\alpha]_D^{20}$): Optical rotation of chiral non-racemic compounds was measured in $CHCl_3$ at 20 °C and 589 nm (sodium D-line) with a Jasco P-2000 polarimeter unless indicated otherwise.

2. Overview of antihistaminic and neuroactive diarylmethanes

All diarylmethane targets that were synthesized in this study are summarized in Figure S1. Generally, all compounds were synthesized from benzyl halides or benzylphosphonium salts in five or six steps with average total yields of 24% and average *ee* values of 76%. We differentiate between two classes of targets: *O*-alkylated compounds **3**, **3'**, **4**, **6**, **7** and *O*-substituted antihistamine **8**. Both synthetic strategies are discussed in detail below.

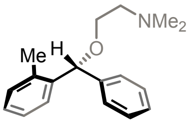

(*R*)-orphenadrine (**3**)

24% yield over 6 steps
(+ 1 recryst.)
98% *ee*

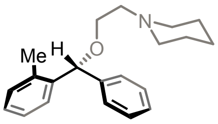

(*R*)-orphenadrine analogue **3'**

18% yield over 6 steps
(+ 1 recryst.)
98% *ee*

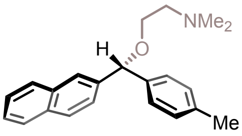

(*R*)-naphthoneobenodine (**4**)

25% yield over 6 steps
(+ 1 recryst.)
87% *ee*

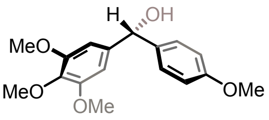

tubulin polymerization inhibitor **2c**

25% yield over 5 steps
(no recryst.)
68% *ee*

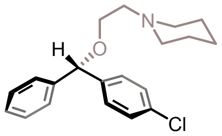

(*S*)-cloperastine (**6**)

15% yield over 5 steps
(+ 1 recryst.)
81% *ee*

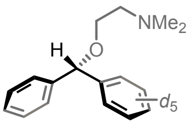

(*S*)-diphenhydramine-d_5 (**7**)

47% yield over 5 steps
(no recryst.)
82% *ee* after MTW

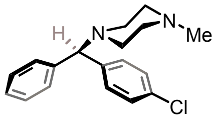

(*R*)-chlorcyclizine (**8**)

12% yield over 6 steps
(+ 1 recryst.)
16% *ee*

Figure S1. Overview of synthesized antihistaminic and neuroactive diarylmethanes.

3. Preparation of photoredox and selenium catalysts

2,4,6-Tris(4-methoxyphenyl)pyrylium tetrafluoroborate (**TAPT**),[2] 1,2-bis(2-methoxyphenyl)diselane (**(2-anisyl-Se)₂**),[3] and (1*S*,1'*S*)-Diselanediylbis(7-methoxy-1,2,3,4-tetrahydronaphthalene-8,1-diyl) bis(2-naphthoate)[4,5] (**C1**) were prepared according to literature procedures. All analytical data was in agreement with published information.

4. Synthesis of *O*-alkylated compounds **3**, **3'**, **4**, **6**, and **7**

Synthesis of *O*-alkylated diarylmethane-based pharmaceuticals was mainly achieved by a linear synthetic route (Scheme S1). As demonstrated in detail in our previous study on migratory Tsuji-Wacker (MTW) oxidations,[1] selection of the migrating arene unit (i.e., the arene ring next to the methyl group) is key for controlling the stereoselective outcome of the reaction. Therefore, a phosphonium salt (**S1–S3**) with the β-arene of interest was synthesized (*General procedure* **A**),[6] which was then reacted with the corresponding methyl aryl ketone in a Wittig olefination reaction (**S4–S8**, *General procedure* **B**).[7] MTW oxidation (*General procedure* **C**) with chiral diselenide **C1** as the co-catalyst sets the absolute configuration in ketones **1** and therefore in the final products. If the inverse enantiomer was required, it could be afforded conveniently by using the constitutional isomer of the stilbene,[1] without a need of synthesizing the opposite enantiomer of catalyst **C1**. A sequence of Baeyer-Villiger oxidation and basic hydrolysis of the crude acetate (*General procedure* **D**)[8] led to benzhydrols **2** usually without any severe erosion of stereoinformation. In addition, these crucial intermediates allowed for recrystallization to further enhance their *ee* values. The total synthesis of amines **3**, **3'**, **4**, **6**, and **7** was completed by Williamson etherification with the desired 2-chloroethane aminium salts (*General procedure* **E**).[9]

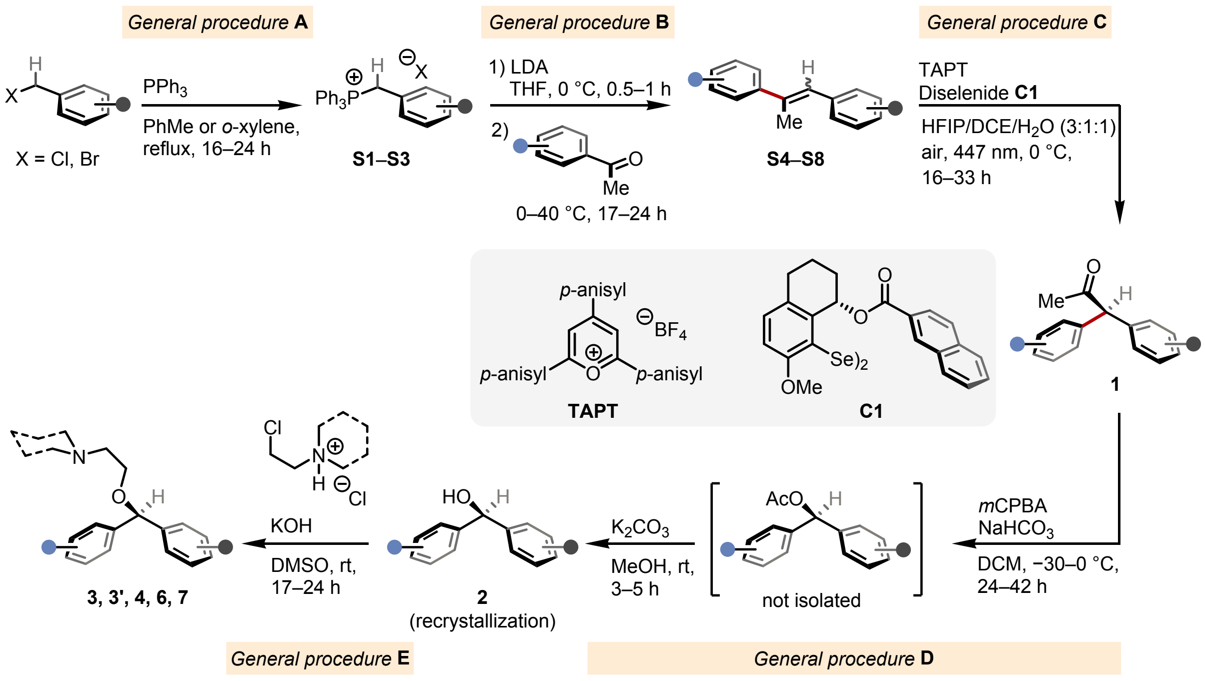

Scheme S1. Overview for synthesis of targets **3**, **3'**, **4**, **6**, and **7**.

General procedure **A** (phosphonium salt synthesis):[6] In a round-bottom flask triphenylphosphine (31.5 mmol, 1.05 equiv.) was dissolved in toluene or *o*-xylene (60 mL, 0.50 M). The respective chloro- or bromomethyl arene (30 mmol, 1.0 equiv.) was added, and the solution was refluxed overnight using a metal heating block. The resulting suspension was cooled slowly to rt, and the precipitate was collected by filtration, washed thoroughly with *n*-hexane, and dried *in vacuo*. The target phosphonium salt was used without further purification for subsequent transformations. Benzyltriphenylphosphonium bromide was commercially available.

(2-Methylbenzyl)triphenylphosphonium bromide (S1)

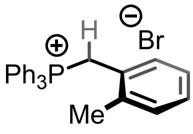

S1

General procedure **A**: Triphenylphosphine (8.26 g, 31.5 mmol, 1.05 equiv.), *o*-xylene (60 mL, 0.50 M), and 2'-methylbenzyl bromide (4.02 mL, 30.0 mmol) were used to afford phosphonium salt **S1** (13.1 g, 29.4 mmol, 98%) as a white solid after 16 h. This compound was fully characterized in our previous publication, where all spectra can be found (see the SI, compound S21).[1]

^{1}H NMR (400 MHz, $CDCl_3$) δ / ppm = 7.71 (t, *J* = 6.3 Hz, 3H), 7.59–7.50 (m, 12H), 7.07 (t, *J* = 6.6 Hz, 1H), 6.96 (d, *J* = 6.9 Hz, 1H), 6.93–6.84 (m, 2H), 5.09 (d, *J* = 14.0 Hz, 2H), 1.58 (s, 3H).

(Naphthalen-2-ylmethyl)triphenylphosphonium bromide (S2)

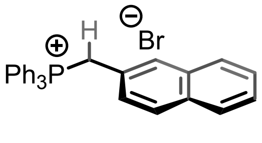

S2

General procedure **A**: Triphenylphosphine (13.9 g, 52.5 mmol, 1.05 equiv.), toluene (50 mL, 1.0 M), and 2-(bromomethyl)naphthalene (11.5 g, 50.0 mmol) were used to afford phosphonium salt **S2** (22.7 g, 47.0 mmol, 94%) as a white solid after 16 h. This compound was fully characterized in our previous publication, where all spectra can be found (see the SI, compound S14).[1]

^{1}H NMR (400 MHz, $CDCl_3$) δ / ppm = 7.72–7.65 (m, 9H), 7.63 (d, *J* = 8.0 Hz, 1H), 7.54 (ddd, *J* = 8.3, 7.2, 3.4 Hz, 6H), 7.50–7.44 (m, 3H), 7.39–7.29 (m, 2H), 7.10 (dt, *J* = 8.4, 1.8 Hz, 1H), 5.47 (d, *J* = 14.5 Hz, 2H).

Triphenyl(3,4,5-trimethoxybenzyl)phosphonium chloride (S3)

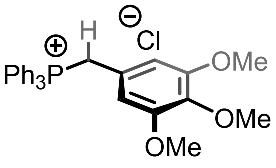

S3

General procedure **A**: Triphenylphosphine (17.4 g, 66.2 mmol, 1.05 equiv.), *o*-xylene (75 mL, 0.80 M), and 5-(chloromethyl)-1,2,3-trimethoxybenzene (13.0 g, 60.1 mmol) were used to afford phosphonium salt **S3** (28.2 g, 58.9 mmol, 98%) as a white solid after 24 h.

m.p. 224.2 °C. **^{1}H NMR** (400 MHz, $CDCl_3$) δ / ppm = 7.92–7.64 (m, 9H), 7.58 (td, *J* = 7.8, 3.3 Hz, 6H), 6.43 (d, *J* = 2.6 Hz, 2H), 5.49 (d, *J* = 14.2 Hz, 2H), 3.73 (s, 3H), 3.47 (s, 6H). **^{13}C{^{1}H} NMR** (101 MHz, $CDCl_3$) δ = 153.1 (d, *J* = 3.8 Hz), 137.7 (d, *J* = 4.8 Hz), 134.8 (d, *J* = 3.0 Hz), 134.7 (d, *J* = 9.7 Hz), 130.1 (d, *J* = 12.5 Hz), 122.9 (d, *J* = 9.1 Hz), 118.1 (d, *J* = 85.6 Hz), 108.9 (d, *J* = 5.6 Hz), 60.9 (d, *J* = 2.4 Hz), 56.2, 30.9 (d, *J* = 46.3 Hz). **^{31}P{^{1}H} NMR** (162 MHz, $CDCl_3$) δ = 24.1. **HRMS** (ESI) calcd. for $[C_{28}H_{28}O_3P{-}Cl]^+$ ($[M{-}Cl]^+$), m/z = 443.1771, found: 443.1773. **IR** (ATR, neat) $\tilde{\nu}$ / cm^{-1} = 3370, 3057, 3004, 2941,1592, 1506, 1462, 1439, 1331, 1249, 1115, 999, 972, 846, 723, 690.

General procedure **B** (Wittig olefination):[7] In a preheated Schlenk flask under nitrogen atmosphere, a phosphonium salt (11 mmol, 1.1 equiv.) was suspended in dry THF (45 mL, 0.20 M), and cooled down to 0 °C using an ice bath. LDA solution (1.5 M in THF/*n*-hexane, 11 mmol, 1.1 equiv.) was added dropwise to the flask, and the resulting mixture was stirred at 0 °C for 30–60 min. The respective ketone (10 mmol, 1.0 equiv., dissolved in 5.0 mL dry THF) was then added slowly to the flask, and the reaction mixture was allowed to warm to rt and subsequently heated to 40 °C overnight using a metal heating block. Upon completion, the reaction was cooled down slowly to rt and quenched with sat. aq. NH_4Cl solution. The phases were separated, and the aqueous layer was extracted with EtOAc. The combined organic layer was washed with water and brine, dried over Na_2SO_4, filtered, and the solvent was removed under reduced pressure. The crude mixture was purified by silica gel column chromatography to afford the target stilbene.

1-Methyl-2-(2-phenylprop-1-en-1-yl)benzene (S4)

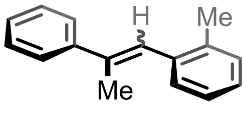

S4

General procedure **B**: Phosphonium salt **S1** (4.92 g, 11.0 mmol, 1.10 equiv.), dry THF (45 mL, 0.20 M), LDA solution (1.5 M, 7.3 mL, 11 mmol, 1.1 equiv.), and acetophenone (1.2 mL, 10 mmol) in 5.0 mL dry THF were used. Purification with silica gel column chromatography

(hexanes:EtOAc = 40:1) afforded an *E*/*Z*-mixture of stilbene **S4** (*E*:*Z* = 42:58, 1.69 g, 8.11 mmol, 81%) as a yellow liquid. This compound was fully characterized in our previous publication, where all spectra can be found (see the SI, compound 1z).[1]

^{1}H NMR (400 MHz, $CDCl_3$) δ / ppm = 7.60–7.55 (m, 2H, *E*), 7.41 (t, *J* = 7.7 Hz, 2H, *E*), 7.35–7.16 (m, 7H, *E*/*Z*), 7.12 (d, *J* = 7.7 Hz, 4H, *E*), 7.03 (t, *J* = 7.4 Hz, 1H, *Z*), 6.91–6.84 (m, 2H, *E*/*Z*), 6.79 (d, *J* = 7.7 Hz, 1H, *Z*), 6.56 (s, 1H, *Z*), 2.33 (s, 3H, *E*), 2.31 (s, 3H, *Z*), 2.28 (d, *J* = 0.9 Hz, 3H, *Z*), 2.15 (d, *J* = 0.8 Hz, 3H, *E*).

2-(2-(*p*-Tolyl)prop-1-en-1-yl)naphthalene (S5)

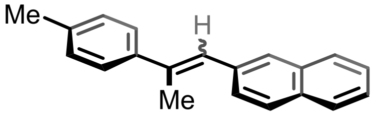

S5

General procedure **B**: Phosphonium salt **S2** (5.32 g, 11.0 mmol, 1.10 equiv.), dry THF (45 mL, 0.20 M), LDA solution (1.9 M, 5.8 mL, 11 mmol, 1.1 equiv.), and 4'-methylacetophenone (1.34 g, 10.0 mmol) in 5.0 mL dry THF were used. Purification with silica gel column chromatography (hexanes:EtOAc = 40:1) afforded an *E*/*Z*-mixture of stilbene **S5** (*E*:*Z* = 51:49, 2.33 g, 9.03 mmol, 90%) as a white solid. This compound was fully characterized in our previous publication, where all spectra can be found (see the SI, compound 1b).[1]

^{1}H NMR (400 MHz, $CDCl_3$) δ / ppm = 7.91–7.84 (m, 4H, *E*), 7.77–7.71 (m, 1H, *Z*), 7.71–7.66 (m, 1H, *Z*), 7.59–7.48 (m, 7H, *E*/*Z*), 7.46–7.39 (m, 2H, *E*/*Z*), 7.29–7.23 (m, 2H, *E*/*Z*), 7.18 (d, *J* = 8.2 Hz, 2H, *E*/*Z*), 7.16–7.11 (m, 2H, *E*/*Z*), 7.10 (dd, *J* = 8.7, 1.6 Hz, 1H, *E*/*Z*), 7.05–7.02 (m, 1H, *E*/*Z*), 6.69–6.66 (m, 1H, *Z*), 2.44 (s, 3H, *E*/*Z*), 2.41 (d, *J* = 1.4 Hz, 3H, *E*), 2.40 (s, 3H, *E*/*Z*), 2.30 (d, *J* = 1.6 Hz, 3H, *Z*).

1,2,3-Trimethoxy-5-(2-(4-methoxyphenyl)prop-1-en-1-yl)benzene (S6)

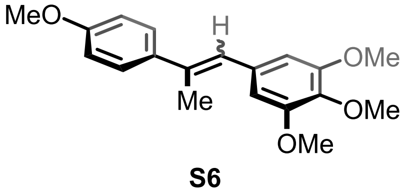

S6

General procedure **B**: Phosphonium salt **S3** (5.27 g, 11.0 mmol, 1.10 equiv.), dry THF (45 mL, 0.20 M), LDA solution (1.7 M, 6.5 mL, 11 mmol, 1.1 equiv.), and 4'-methoxyacetophenone (1.50 g, 10.0 mmol) in 5.0 mL dry THF were used. Purification with silica gel column chromatography (hexanes:MeOH = 19:1, then DCM:EtOAc = 10:1, then hexanes:Et_2O = 3:1) afforded an *E*/*Z*-mixture of stilbene **S6** (*E*:*Z* = 49:51, 2.69 g, 8.55 mmol, 86%) as a white solid.

m.p. 58.1 °C. **TLC** R_f = 0.24 (hexanes:Et_2O = 3:1). **^{1}H NMR** (400 MHz, $CDCl_3$) δ / ppm = 7.49–7.43 (m, 2H, *E*), 7.17–7.11 (m, 2H, *Z*), 6.94–6.88 (m, 2H, *E*), 6.88–6.83 (m, 2H, *Z*), 6.74–6.69

(m, 1H, *E*), 6.58 (s, 2H, *E*), 6.35 (d, *J* = 1.4 Hz, 1H, *Z*), 6.21 (s, 2H, *Z*), 3.88 (d, *J* = 1.3 Hz, 9H, *E*), 3.84 (s, 3H, *E*), 3.79 (d, *J* = 1.8 Hz, 6H, *Z*), 3.58 (s, 6H, *Z*), 2.28 (d, *J* = 1.3 Hz, 3H, *E*), 2.18 (d, *J* = 1.5 Hz, 3H, *Z*). **^{13}C{^{1}H} NMR** (101 MHz, $CDCl_3$) δ = 159.1 (*E*), 158.7 (*Z*), 153.0 (*E*), 152.6 (*Z*), 138.3 (*Z*), 136.8 (*E*), 136.7 (*E*), 136.5 (*Z*), 136.4 (*E*), 134.6 (*Z*), 134.3 (*E*), 133.3 (*Z*), 129.5 (*Z*), 127.1 (*E*), 126.4 (*E*), 126.1 (*Z*), 114.1 (*E*), 113.8 (*Z*), 106.5 (*E*), 106.2 (*Z*), 61.1 (*E*), 61.0 (*Z*), 56.3 (*E*), 55.8 (*Z*), 55.5 (*E*/*Z*), 55.4 (*E*/*Z*), 27.3 (*Z*), 17.8 (*E*). **HRMS** (ESI) calcd. for $[C_{19}H_{22}O_4+H]^+$ ($[M+H]^+$), m/z = 315.1591, found: 315.1593. **IR** (ATR, neat) $\tilde{\nu}$ / cm^{-1} = 2933, 2837, 1607, 1580, 1506, 1461, 1416, 1331, 1286, 1238, 1178, 1126, 1029, 872, 831.

1-Chloro-4-(1-phenylprop-1-en-2-yl)benzene (S7)

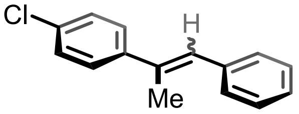

S7

General procedure **B**: Benzyltriphenylphosphonium bromide (4.77 g, 11.0 mmol, 1.10 equiv.), dry THF (45 mL, 0.20 M), LDA solution (0.83 M, 13 mL, 11 mmol, 1.1 equiv.), and 4’-chloroacetophenone (1.30 mL, 10.0 mmol) in 5.0 mL dry THF were used. Purification with silica gel column chromatography (hexanes:EtOAc = 40:1) afforded an *E*/*Z*-mixture of stilbene **S7** (*E*:*Z* = 57:43, 2.11 g, 9.23 mmol, 92%) as a white semi-solid.

TLC R_f = 0.58 (hexanes:EtOAc = 19:1). **^{1}H NMR** (400 MHz, $CDCl_3$) δ / ppm = 7.61–7.55 (m, 2H, *E*), 7.55–7.44 (m, 7H, *E*/*Z*), 7.43–7.34 (m, 3H, *E*/*Z*), 7.29–7.20 (m, 4H, *E*/*Z*), 7.13–7.07 (m, 2H, *Z*), 6.96 (s, 1H, *E*), 6.63 (s, 1H, *Z*), 2.39 (d, *J* = 1.3 Hz, 3H, *E*), 2.32 (d, *J* = 1.5 Hz, 3H, *Z*). **^{13}C{^{1}H} NMR** (101 MHz, $CDCl_3$) δ = 142.4, 140.5, 138.1, 137.4, 137.4, 136.3, 133.0, 132.8, 129.8, 129.3, 129.1, 128.8, 128.5, 128.4, 128.2, 128.1, 127.4, 126.8, 126.5, 26.9, 17.5. **HRMS** (EI) calcd. for $[C_{15}H_{13}Cl]^{\bullet+}$ ($[M]^{\bullet+}$), m/z = 228.0700, found: 228.0702. **IR** (ATR, neat) $\tilde{\nu}$ / cm^{-1} = 3057, 3023, 2967, 2915, 2855, 1897, 1595, 1491, 1439, 1402, 1178, 1092, 1014, 917, 857, 820, 746, 697.

1-(1-Phenylprop-1-en-2-yl)benzene-2,3,4,5,6-d_5 (S8)

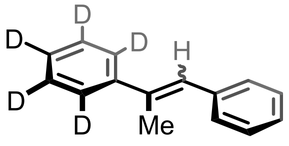

S8

General procedure **B**: Benzyltriphenylphosphonium bromide (4.77 g, 11.0 mmol, 1.10 equiv.), dry THF (45 mL, 0.20 M), LDA solution (1.0 M, 11 mL, 11 mmol, 1.1 equiv.), and 1-(phenyl-d_5)ethan-1-one (1.17 mL, 10.0 mmol) in 5.0 mL dry THF were used. Purification with silica gel column chromatography (hexanes:EtOAc = 30:1) afforded an *E*/*Z*-mixture of stilbene **S8** (*E*:*Z* = 53:47, 1.67 g, 8.38 mmol, 84%) as a white solid. This compound was fully characterized in our previous publication, where all spectra can be found (see the SI, compound 1h’-d_5).[1]

^{1}**H NMR** (400 MHz, $CDCl_3$) δ / ppm = 7.42 (m, 4H, *E*/*Z*), 7.33–7.26 (m, 1H, *Z*), 7.17–7.07 (m, 3H, *E*/*Z*), 7.02–6.97 (m, 2H, *E*/*Z*), 6.90 (d, *J* = 1.2 Hz, 1H, *E*), 6.52 (s, 1H, *Z*), 2.33 (d, *J* = 1.3 Hz, 3H, *E*), 2.25 (d, *J* = 1.5 Hz, 3H, *Z*).

General procedure **C** (asymmetric migratory Tsuji-Wacker oxidation):[1] A 100 mL round-bottom flask equipped with a cross-shaped stirring bar, was charged with a stilbene (indicated *E*/*Z*-ratio, 2 mmol, 1 equiv.), selenium catalyst **C1** (200 µmol, 10.0 mol%), and TAPT (0.10 mmol, 5.0 mol%). A 3:1:1 volumetric ratio of HFIP, DCE, and H_2O (20 mL in total, 0.10 M) was added. The flask was sealed with a rubber septum and equipped with needles for air supply. The solution was stirred with 550 rpm under irradiation of blue light (447 nm) at 0 °C in a metal cooling block for a certain amount of time to achieve full consumption of the stilbene. For a detailed description of the irradiation setup, see the General Remarks section and the SI of our previous publication (Figure S1 therein).[1] While not being fully dissolved at the beginning, the stilbene was consumed over time during irradiation. The solvent of the crude mixture was removed under reduced pressure, and the residue was purified by silica gel column chromatography to afford the target ketone. Determination of the ketone's absolute configuration is based on analogy to our previous publication,[1] namely the crystal structure of ketone 2a therein, which was determined to be (*R*).

For determination of retention times for chiral HPLC analysis, the respective stilbene (0.5 mmol, 1 equiv.), (2-anisyl-Se)$_2$ (50 µmol, 10 mol%), and TAPT (25 µmol, 5.0 mol%) in a 3:1:1 volumetric ratio of HFIP, DCE, and H_2O (5 mL in total, 0.1 M) were used under otherwise identical conditions.

(*R*)-1-Phenyl-1-(*o*-tolyl)propan-2-one (1a)

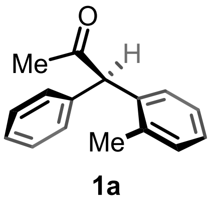

1a

General procedure **C**: Stilbene **S4** (*E*:*Z* = 42:58, 417 mg, 2.00 mmol), selenium catalyst **C1** (164 mg, 200 µmol, 10.0 mol%), TAPT (49 mg, 0.10 mmol, 5.0 mol%), and HFIP, DCE, and H_2O (3:1:1, 20 mL in total, 0.10 M) were used. Reaction time was 28 h. Purification with silica gel column chromatography (hexanes:EtOAc = 19:1) afforded ketone **1a** (384 mg, 1.71 mmol, 86%, 91% *ee*) as a colorless oil. This compound was fully characterized in our previous publication, where all spectra can be found (see the SI, compound 2z).[1]

^{1}**H NMR** (400 MHz, $CDCl_3$) δ / ppm = 7.36–7.30 (m, 2H), 7.30–7.24 (m, 1H), 7.23–7.12 (m, 5H), 7.09–7.03 (m, 1H), 5.28 (s, 1H), 2.30 (s, 3H), 2.25 (s, 3H). **HPLC** (IC-3,

n-hexane:*i*-PrOH 99:1, flow rate 0.6 mL/min, 220 nm, 25 °C) t_R = 32.200 min (4.4%), 41.384 min (95.6%). **Optical Rotation** $[\alpha]_D^{20}$ = −85.6 (*c* = 1.0, $CHCl_3$).

(*R*)-1-(Naphthalen-2-yl)-1-(*p*-tolyl)propan-2-one (1b)

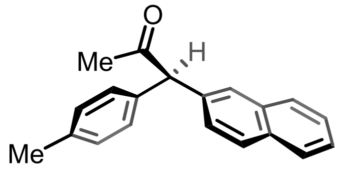

1b

General procedure **C**: Stilbene **S5** (*E*:*Z* = 51:49, 517 mg, 2.00 mmol), selenium catalyst **C1** (164 mg, 200 µmol, 10.0 mol%), TAPT (49 mg, 0.10 mmol, 5.0 mol%), and HFIP, DCE, and H_2O (3:1:1, 20 mL in total, 0.10 M) were used. Reaction time was 16 h. Purification with silica gel column chromatography (hexanes:EtOAc = 20:1) afforded ketone **1b** (433 mg, 1.58 mmol, 79%, 87% *ee*) as a yellow oil. This compound was fully characterized in our previous publication, where all spectra can be found (see the SI, compound 2b).[1]

^{1}H NMR (400 MHz, $CDCl_3$) δ / ppm = 7.81 (tt, *J* = 7.8, 7.0, 2.8 Hz, 3H), 7.67 (dd, *J* = 1.8, 0.8 Hz, 1H), 7.50–7.44 (m, 2H), 7.36 (dd, *J* = 8.5, 1.9 Hz, 1H), 7.17 (s, 4H), 5.27 (s, 1H), 2.35 (s, 3H), 2.30 (s, 3H). **HPLC** (ID-3, *n*-hexane:*i*-PrOH 98:2, flow rate 0.8 mL/min, 220 nm, 25 °C) t_R = 13.123 min (93.5%), 14.288 min (6.5%). **Optical Rotation** $[\alpha]_D^{20}$ = −13.5 (*c* = 1.0, $CHCl_3$).

(*R*)-1-Phenyl-1-(*p*-tolyl)propan-2-one (1c)

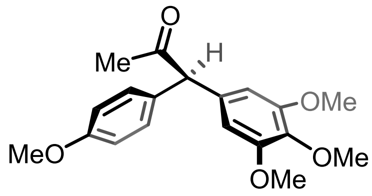

1c

General procedure **C**: Stilbene **S6** (*E*:*Z* = 49:51, 315 mg, 1.00 mmol), selenium catalyst **C1** (82 mg, 0.10 mmol, 10 mol%), TAPT (24 mg, 50 µmol, 5.0 mol%), and HFIP, DCE, and H_2O (3:1:1, 10 mL in total, 0.10 M) were used. Reaction time was 25 h. Purification with silica gel column chromatography (hexanes:EtOAc = 4:1 to 3:2) afforded ketone **1c** (191 mg, 579 µmol, 58%, 75% *ee*) as a yellow oil.

TLC R_f = 0.49 (hexanes:EtOAc = 4:1). **^{1}H NMR** (400 MHz, $CDCl_3$) δ / ppm = 7.18–7.08 (m, 2H), 6.91–6.82 (m, 2H), 6.42 (s, 2H), 4.99 (s, 1H), 3.83 (s, 3H), 3.81 (s, 6H), 3.79 (s, 3H), 2.25 (s, 3H). **^{13}C{^{1}H} NMR** (101 MHz, $CDCl_3$) δ = 206.9, 158.9, 153.5, 137.3, 134.1, 130.3, 130.1, 114.3, 106.2, 64.3, 61.0, 56.3, 55.4, 30.1. **HRMS** (ESI) calcd. for $[C_{19}H_{22}O_5+H]^+$ ($[M+H]^+$), m/z = 331.1540, found: 331.1544. **IR** (ATR, neat) $\tilde{\nu}$ / cm^{-1} = 2997, 2937, 2840, 1715, 1588, 1510, 1461, 1424, 1331, 1249, 1182, 1126, 1033, 828, 731. **HPLC** (IA-3, *n*-hexane:*i*-PrOH 95:5, flow rate 0.8 mL/min, 220 nm, 25 °C) t_R = 42.838 min (87.7%), 46.162 min (12.3%). **Optical Rotation** $[\alpha]_D^{20}$ = −18.9 (*c* = 1.0, $CHCl_3$).

(*S*)-1-(4-Chlorophenyl)-1-phenylpropan-2-one (1d)

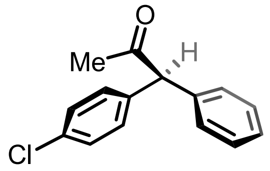

1d

General procedure **C**: Stilbene **S7** (*E*:*Z* = 57:43, 457 mg, 2.00 mmol), selenium catalyst **C1** (164 mg, 200 μmol, 10.0 mol%), TAPT (49 mg, 0.10 mmol, 5.0 mol%), and HFIP, DCE, and H_2O (3:1:1, 20 mL in total, 0.10 M) were used. Reaction time was 33 h. Purification with silica gel column chromatography (hexanes:EtOAc = 20:1) afforded ketone **1d** (220 mg, 900 μmol, 45%, 88% *ee*) as a colorless oil.

TLC R_f = 0.15 (hexanes:EtOAc = 19:1). **^{1}H NMR** (400 MHz, $CDCl_3$) δ / ppm = 7.35 (tt, *J* = 8.2, 1.8 Hz, 2H), 7.32–7.27 (m, 3H), 7.21 (dd, *J* = 7.1, 1.6 Hz, 2H), 7.17–7.13 (m, 2H), 5.08 (s, 1H), 2.24 (s, 3H). **^{13}C{^{1}H} NMR** (101 MHz, $CDCl_3$) δ = 206.1, 137.9, 137.0, 133.3, 130.4, 129.1, 129.0, 128.9, 127.7, 64.4, 30.2. **HRMS** (EI) calcd. for $[C_{15}H_{13}ClO]^{\bullet+}$ ($[M]^{\bullet+}$), m/z = 244.0649, found: 244.0645. **IR** (ATR, neat) $\tilde{\nu}$ / cm^{-1} = 3064, 3030, 1715, 1599, 1491, 1409, 1357, 1156, 1135, 1092, 1014, 813, 753, 701. **HPLC** (ID-3, *n*-hexane:*i*-PrOH 98:2, flow rate 0.8 mL/min, 220 nm, 25 °C) t_R = 8.963 min (94.0%), 9.342 min (6.0%). **Optical Rotation** $[\alpha]_D^{20}$ = −40.0 (*c* = 1.0, $CHCl_3$).

(*S*)-1-Phenyl-1-(phenyl-*d*$_5$)propan-2-one (1e)

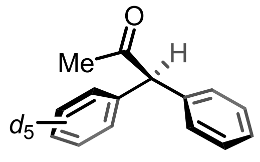

1e

General procedure **C**: Stilbene **S8** (*E*:*Z* = 53:47, 399 mg, 2.00 mmol), selenium catalyst **C1** (164 mg, 200 μmol, 10.0 mol%), TAPT (49 mg, 0.10 mmol, 5.0 mol%), and HFIP, DCE, and H_2O (3:1:1, 20 mL in total, 0.10 M) were used. Reaction time was 17 h. Purification with silica gel column chromatography (hexanes:EtOAc = 20:1) afforded ketone **1e** (370 mg, 1.72 mmol, 86%, 82% *ee*) as a colorless oil. This compound was fully characterized in our previous publication, where all spectra can be found (see the SI, compound 2h'-*d*$_5$).[1]

^{1}H NMR (400 MHz, $CDCl_3$) δ / ppm = 7.37–7.32 (m, 2H), 7.30–7.27 (m, 1H), 7.25–7.22 (m, 2H), 5.13 (s, 1H), 2.25 (s, 3H).

General procedure **D** (Baeyer-Villiger oxidation and subsequent basic hydrolysis):[8] In a preheated Schlenk flask under nitrogen atmosphere, the respective ketone **1** (1.6 mmol, 1.0 equiv.) was dissolved in dry DCM (4.5 mL, 0.36 M). $NaHCO_3$ (4.8 mmol, 3.0 equiv.) was added, and the reaction mixture was cooled to 0 °C or −30 °C using a cryostat. A suspension of *m*CPBA (2.4 mmol, 1.5 equiv. in dry DCM) was added dropwise, and the reaction mixture

was allowed to warm to rt and stirred for a certain amount of time. If the SM was not fully consumed, another 1.0–1.5 equiv. of $NaHCO_3$ and *m*CPBA would be added, respectively. Upon completion, the solution was diluted with DCM, quenched with 10% (*w*/*w*) aq. Na_2SO_3 solution, and washed with sat. aq. $NaHCO_3$ solution. The organic layer was dried over $MgSO_4$, filtered, and the solvent was removed under reduced pressure.

The crude acetate was dissolved in MeOH (4 mL, 0.4 M), and K_2CO_3 (4.7 mmol, 3.0 equiv.) was added. The reaction mixture was stirred at rt for a certain amount of time until full conversion was achieved. The solution was carefully neutralized with 1 M HCl and extracted with EtOAc. The combined organic layers were washed with brine, dried over $MgSO_4$, filtered, and the solvent was removed under reduced pressure. The pure product was obtained from silica gel column chromatography. An additional recrystallization step to enhance the compound's *ee* was carried out by dissolving the respective alcohol in the minimum possible amount of boiling hot *n*-hexane and letting the solution cool down slowly to rt. If no precipitation occurred, the solution was put in the fridge (3 °C) for 2 h. The resulting crystals were collected by filtration and washed carefully with cold *n*-hexane, if not stated otherwise.

(*R*)-Phenyl(*o*-tolyl)methanol (2a)

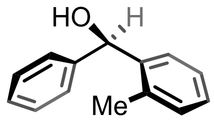

2a

General procedure **D**: Ketone **1a** (358 mg, 1.59 mmol, 91% *ee*) in dry DCM (5 mL, 0.3 M), $NaHCO_3$ (402 mg, 4.78 mmol, 3.00 equiv.), and *m*CPBA (413 mg, 2.39 mmol, 1.50 equiv.) in 4.5 mL dry DCM were used. Reaction time was 25 h. Hydrolysis of the crude acetate with K_2CO_3 (649 mg, 4.69 mmol, 3.00 equiv.) in MeOH (4 mL, 0.4 M) took 4 h. Purification with silica gel column chromatography (*n*-pentane:EtOAc = 19:1) afforded benzhydrol **2a** (250 mg, 1.26 mmol, 79% over two steps, 87% *ee*) as a white solid. Recrystallization from hot *n*-hexane and concentration of the mother liquor afforded **2a** (126 mg, 630 µmol, 50%) with an enhanced *ee* value of 98%.

m.p. = 50.7 °C. **TLC** R_f = 0.15 (*n*-pentane:EtOAc = 19:1). **^{1}H NMR** (400 MHz, $CDCl_3$) δ / ppm = 7.52 (dd, *J* = 7.5, 1.3 Hz, 1H), 7.33 (d, *J* = 4.4 Hz, 4H), 7.30–7.23 (m, 2H), 7.21 (td, *J* = 7.4, 1.7 Hz, 1H), 7.15 (d, *J* = 7.5 Hz, 1H), 6.02 (d, *J* = 3.5 Hz, 1H), 2.26 (s, 3H), 2.10 (d, *J* = 4.1 Hz, 1H). **^{13}C{^{1}H} NMR** (101 MHz, $CDCl_3$) δ / ppm = 143.0, 141.6, 135.5, 130.7, 128.6, 127.7, 127.7, 127.2, 126.4, 126.3, 77.2, 73.5, 19.5. **HRMS** (EI) calcd. for $[C_{14}H_{14}O]^{\bullet+}$ ($[M]^{\bullet+}$), m/z = 198.1039, found: 198.1043. **IR** (ATR, neat) $\tilde{\nu}$ / cm^{-1} = 3336, 3064, 3027, 2922, 2855, 1491, 1454, 1178, 1018, 760. **HPLC** (IC-3, *n*-hexane:*i*-PrOH 95:5, flow rate 0.8 mL/min, 220 nm, 25 °C) t_R = 9.334 min (1.2%), 10.026 min (98.8%). **Optical Rotation** $[\alpha]_D^{20}$ = −6.8 (*c* = 1.0, $CHCl_3$).

(*R*)-Naphthalen-2-yl(*p*-tolyl)methanol (2b)

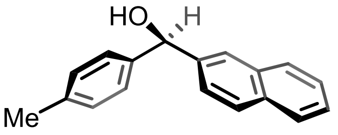

2b

General procedure **D**: Ketone **1b** (370 mg, 1.35 mmol, 87% *ee*) in dry DCM (4.5 mL, 0.2 M), $NaHCO_3$ (340 mg, 4.05 mmol, 3.00 equiv.), and *m*CPBA (349 mg, 2.02 mmol, 1.50 equiv.) in 4.5 mL dry DCM were used. Re-addition of $NaHCO_3$ (113 mg, 1.35 mmol, 1.00 equiv.) and *m*CPBA (233 mg, 1.35 mmol, 1.00 equiv.) in 3 mL dry DCM after 24 h. Reaction time was 42 h in total. Hydrolysis of the crude acetate with K_2CO_3 (559 mg, 4.05 mmol, 3.00 equiv.) in MeOH (4 mL, 0.3 M) took 5 h. Purification with silica gel column chromatography (hexanes:EtOAc = 10:1) afforded benzhydrol **2b** (171 mg, 689 µmol, 51% over two steps, 77% *ee*) as a white solid. Recrystallization from hot *n*-hexane afforded **2b** (151 mg, 606 µmol, 88%) with an enhanced *ee* value of 90%.

m.p. = 99.8 °C. **TLC** R_f = 0.13 (hexanes:EtOAc = 9:1). **^{1}H NMR** (300 MHz, $CDCl_3$) δ / ppm = 7.91 (s, 1H), 7.87–7.76 (m, 3H), 7.53–7.39 (m, 3H), 7.31 (d, *J* = 8.1 Hz, 2H), 7.15 (d, *J* = 7.9 Hz, 2H), 5.98 (s, 1H), 2.34 (s, 3H), 2.29 (br, 1H). **^{13}C{^{1}H} NMR** (75 MHz, $CDCl_3$) δ / ppm = 141.4, 140.9, 137.5, 133.4, 132.9, 129.4, 128.4, 128.2, 127.8, 126.8, 126.3, 126.0, 125.0, 124.9, 76.3, 21.3. **HRMS** (EI) calcd. for $[C_{18}H_{16}O]^{\bullet+}$ ($[M]^{\bullet+}$), m/z = 248.1196, found: 248.1200. **IR** (ATR, neat) $\tilde{\nu}$ / cm^{-1} = 3314, 3049, 2922, 2855, 1510, 1122, 1025, 820, 760. **HPLC** (OD-3, *n*-hexane:*i*-PrOH 95:5, flow rate 0.8 mL/min, 250 nm, 25 °C) t_R = 27.963 min (5.2%), 31.405 min (94.8%). **Optical Rotation** $[\alpha]_D^{20}$ = −13.1 (*c* = 1.0, $CHCl_3$).

(*R*)-(4-Methoxyphenyl)(3,4,5-trimethoxyphenyl)methanol (2c)

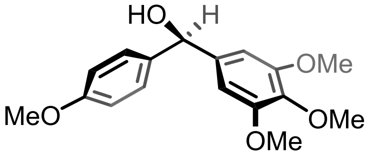

2c

General procedure **D**: Ketone **1c** (173 mg, 523 µmol, 75% *ee*) in dry DCM (3.5 mL, 0.15 M), $NaHCO_3$ (66 mg, 0.79 mmol, 1.5 equiv.), and *m*CPBA (129 mg, 525 µmol, 1.00 equiv.) in 3 mL dry DCM were used at −30 °C. Re-addition of $NaHCO_3$ (66 mg, 0.79 mmol, 1.5 equiv.) and *m*CPBA (198 mg, 803 µmol, 1.50 equiv.) in 2 mL dry DCM after 7 h afforded crude acetate **S9** after a total reaction time of 24 h.

^{1}H NMR (400 MHz, $CDCl_3$) δ / ppm = 7.14 (d, *J* = 8.6 Hz, 2H), 6.88 (d, *J* = 8.7 Hz, 2H), 6.42 (s, 2H), 4.99 (s, 1H), 3.83 (s, 3H), 3.81 (s, 6H), 3.79 (s, 6H), 2.25 (s, 3H).

Hydrolysis of acetate **S9** with K_2CO_3 (225 mg, 1.63 mmol, 3.00 equiv.) in MeOH (3 mL, 0.2 M) took 3 h. Purification with silica gel column chromatography (hexanes:DCM:EtOAc = 4:2:1) afforded benzhydrol **2c** (81.1 mg, 267 µmol, 51% over two steps, 68% *ee*) as an orange solid.

m.p. = 91.7 °C. **TLC** R_f = 0.20 (hexanes:DCM:EtOAc = 40:20:15). **^{1}H NMR** (400 MHz, $CDCl_3$) δ / ppm = 7.28 (d, *J* = 8.6 Hz, 2H), 6.87 (d, *J* = 8.6 Hz, 2H), 6.60 (s, 2H), 5.73 (s, 1H), 3.82 (s,

9H), 3.79 (s, 3H), 2.27 (br, 1H). **^{13}C{^{1}H} NMR** (101 MHz, $CDCl_3$) δ / ppm = 159.2, 153.3, 139.8, 137.2, 136.1, 128.0, 114.0, 103.5, 76.0, 61.0, 56.2, 55.4. **HRMS** (ESI) calcd. for $[C_{17}H_{20}O_5+H]^+$ ($[M+H]^+$), m/z = 305.1372, found: 305.1372. **IR** (ATR, neat) $\tilde{\nu}$ / cm^{-1} = 3463, 3001, 2937, 2840, 1737, 1592, 1510, 1461, 1420, 1327, 1238, 1178, 1126, 1033, 835. **HPLC** (IB-3, *n*-hexane:*i*-PrOH 90:10 to 80:20, flow rate 0.8 mL/min, 220 nm, 25 °C) t_R = 20.737 min (16.0%), 23.710 min (84.0%). **Optical Rotation** $[\alpha]_D^{20}$ = −12.8 (*c* = 1.0, $CHCl_3$).

(*S*)-(4-Chlorophenyl)(phenyl)methanol (2d)

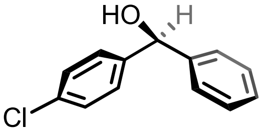

2d

General procedure **D**: Ketone **1d** (295 mg, 1.21 mmol, 88% *ee*) in dry DCM (5.0 mL, 0.24 M), $NaHCO_3$ (304 mg, 3.62 mmol, 3.00 equiv.), and *m*CPBA (446 mg, 1.81 mmol, 1.50 equiv.) in 3 mL dry DCM were used. Re-addition of $NaHCO_3$ (101 mg, 1.20 mmol, 1.00 equiv.) and *m*CPBA (297 mg, 1.21 mmol, 1.00 equiv.) in 3 mL dry DCM after 28 h. Reaction time was 31 h in total. Hydrolysis of the crude acetate with K_2CO_3 (524 mg, 3.79 mmol, 3.00 equiv.) in MeOH (5 mL, 0.2 M) took 3 h. Purification with silica gel column chromatography (hexanes:DCM:EtOAc = 40:20:1) afforded benzhydrol **2d** (215 mg, 980 µmol, 81% over two steps, 80% *ee*) as a white solid. Recrystallization from hot *n*-hexane afforded **2d** (109 mg, 500 µmol, 51%) with an enhanced *ee* value of 95%.

m.p. = 60.9 °C. **TLC** R_f = 0.19 (hexanes:DCM:EtOAc = 40:20:1). **^{1}H NMR** (400 MHz, $CDCl_3$) δ / ppm = 7.35 (d, *J* = 4.4 Hz, 4H), 7.31 (s, 4H), 7.29 (d, *J* = 4.6 Hz, 1H), 5.81 (d, *J* = 2.7 Hz, 1H), 2.15 (br, 1H). **^{13}C{^{1}H} NMR** (101 MHz, $CDCl_3$) δ / ppm = 143.6, 142.3, 133.4, 128.8, 128.7, 128.0, 126.7, 75.8. **HRMS** (EI) calcd. for $[C_{13}H_{11}ClO]^{\bullet+}$ ($[M]^{\bullet+}$), m/z = 218.0493, found: 218.0488. **IR** (ATR, neat) $\tilde{\nu}$ / cm^{-1} = 3567, 3262, 3064, 3030, 2878, 1599, 1491, 1405, 1185, 1088, 1014, 850, 798, 757, 701. **HPLC** (IA-3, *n*-hexane:*i*-PrOH 98:2, flow rate 0.8 mL/min, 220 nm, 25 °C) t_R = 30.351 min (2.5%), 32.690 min (97.5%). **Optical Rotation** $[\alpha]_D^{20}$ = +20.2 (*c* = 1.0, $CHCl_3$).

(*S*)-Phenyl(phenyl-d_5)methanol (2e)

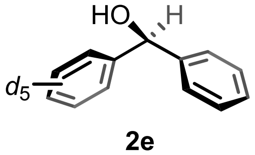

2e

General procedure **D**: Ketone **1e** (343 mg, 1.59 mmol, 82% *ee*) in dry DCM (5 mL, 0.8 M), $NaHCO_3$ (401 mg, 4.78 mmol, 3.00 equiv.), and *m*CPBA (412 mg, 2.39 mmol, 1.50 equiv.) in 3 mL dry DCM were used. Reaction time was 24 h. Hydrolysis of the crude acetate with K_2CO_3 (647 mg, 4.68 mmol, 3.00 equiv.) in MeOH (5 mL, 0.3 M) took 4 h. Purification with silica gel column chromatography (hexanes:EtOAc = 9:1) afforded benzhydrol **2e** (227 mg, 1.20 mmol,

77% over two steps) as a white solid. Determination of *ee* value with chiral HPLC is impossible due to a lack of differentiation of the two enantiomers (estimation: 68% < *ee* < 82%).

m.p. = 67.4 °C. **TLC** R_f = 0.15 (hexanes:EtOAc = 9:1). **^{1}H NMR** (400 MHz, $CDCl_3$) δ / ppm = 7.39 (dd, *J* = 8.3, 1.4 Hz, 2H), 7.37–7.32 (m, 2H), 7.30–7.24 (m, 1H), 5.86 (s, 1H), 2.42–1.83 (br, 1H). **^{2}H NMR** (92 MHz, $CHCl_3$) δ / ppm = 7.47–7.31 (m, 5H). **^{13}C{^{1}H} NMR** (126 MHz, $CDCl_3$) δ / ppm = 143.9, 143.8, 128.6, 128.1 (t, *J* = 24.3 Hz), 127.7, 127.2 (t, *J* = 25.1 Hz), 126.7, 126.2 (t, *J* = 24.2 Hz), 76.4. **HRMS** (EI) calcd. for $[C_{13}H_7D_5O]^{\bullet+}$ ($[M]^{\bullet+}$), m/z = 189.1200, found: 189.1202. **IR** (ATR, neat) $\tilde{\nu}$ / cm^{-1} = 3545, 3362, 3064, 3030, 2878, 2274, 1495, 1454, 1137, 1047, 1014, 760, 731, 701.

General procedure **E** (Williamson etherification):[9] A preheated Schlenk flask was charged with the respective benzhydrol **2** (0.33 mmol, 1.0 equiv.), KOH (3.3 mmol, 10 equiv.) and an aminium chloride (0.66 mmol, 2 equiv.) under nitrogen atmosphere. This mixture was suspended in dry DMSO (1.5 mL, 0.22 M) and stirred overnight at rt. If the SM was not fully consumed, another 1 equiv. of the aminium chloride would be added. Upon completion, the reaction was quenched with 1 M aq. NaOH solution, diluted with Et_2O and stirred for 45 min. The layers were separated, and the aqueous layer was extracted with Et_2O. The combined organic layer was washed with 1 M aq. NaOH solution, dried over Na_2SO_4, filtered, and the solvent was removed under reduced pressure. The target compound was afforded by silica gel column chromatography.

(*R*)-*N,N*-Dimethyl-2-(phenyl(*o*-tolyl)methoxy)ethan-1-amine (3)

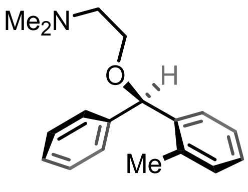

3

General procedure **E**: Benzhydrol **2a** (64.7 mg, 326 μmol, 98% *ee*), KOH (183 mg, 3.26 mmol, 10.0 equiv.) and 2-chloro-*N,N*-dimethylethan-1-aminium chloride (94.2 mg, 654 μmol, 2.00 equiv.) in dry DMSO (1.5 mL, 0.22 M) were used. Reaction time was 17 h. Purification with silica gel column chromatography (hexanes:EtOAc = 7:3 + 1% (*v*/*v*) NEt_3 to 3:2 + 1% (*v*/*v*) NEt_3) afforded (*R*)-orphenadrine (**3**) (78.5 mg, 291 μmol, 89%, 98% *ee*) as a yellow oil.

TLC R_f = 0.18 (hexanes:EtOAc = 7:3 + 1% (*v*/*v*) NEt_3). **^{1}H NMR** (400 MHz, $CDCl_3$) δ / ppm = 7.43 (dd, *J* = 7.3, 1.7 Hz, 1H), 7.34–7.27 (m, 4H), 7.25–7.15 (m, 3H), 7.15–7.09 (m, 1H), 5.54 (s, 1H), 3.57 (td, *J* = 6.1, 2.4 Hz, 2H), 2.59 (t, *J* = 6.1 Hz, 2H), 2.26 (s, 9H). **^{13}C{^{1}H} NMR** (101 MHz, $CDCl_3$) δ / ppm = 141.3, 140.0, 136.0, 130.6, 128.4, 127.7, 127.5, 127.5, 127.2, 126.1, 81.5, 67.7, 59.2, 46.2, 19.6. **HRMS** (ESI) calcd. for $[C_{18}H_{23}NO+H]^+$ ($[M+H]^+$), m/z =

270.1852, found: 270.1858. **IR** (ATR, neat) $\tilde{\nu}$ / cm^{-1} = 3064, 3027, 2941, 2863, 2818, 2769, 1491, 1454, 1305, 1178, 1096, 753. **HPLC** (IB-3, *n*-hexane:(*i*-PrOH:EtOH = 1:1 + 0.5% (*v*/*v*) Et_2NH) 95:5, flow rate 0.8 mL/min, 220 nm, 20 °C) t_R = 6.051 min (98.9%), 6.885 min (1.1%). **Optical Rotation** $[\alpha]_D^{20}$ = +8.9 (*c* = 1.0, THF).

(*R*)-1-(2-(Phenyl(*o*-tolyl)methoxy)ethyl)piperidine (3')

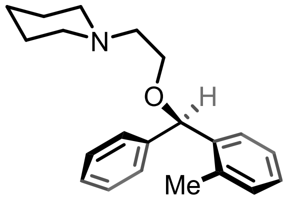

3'

General procedure **E**: Benzhydrol **2a** (35.0 mg, 177 µmol, 98% *ee*), KOH (99.0 mg, 1.77 mmol, 10.0 equiv.) and 1-(2-chloroethyl)-piperidin-1-ium chloride (65.0 mg, 353 µmol, 2.00 equiv.) in dry DMSO (2 mL, 0.1 M) were used. Reaction time was 17 h. Purification with silica gel column chromatography (hexanes:EtOAc = 9:1 + 1% (*v*/*v*) NEt_3) afforded (*R*)-orphenadrine analogue **3'** (36.1 mg, 117 µmol, 66%, 98% *ee*) as a yellow oil.

TLC R_f = 0.21 (hexanes:EtOAc = 9:1 + 1% (*v*/*v*) NEt_3). **^{1}H NMR** (400 MHz, $CDCl_3$) δ / ppm = 7.41 (dd, *J* = 7.3, 1.7 Hz, 1H), 7.30 (d, *J* = 4.4 Hz, 4H), 7.25–7.15 (m, 3H), 7.14–7.10 (m, 1H), 5.55 (s, 1H), 3.60 (tt, *J* = 6.1, 3.1 Hz, 2H), 2.64 (t, *J* = 6.3 Hz, 2H), 2.44 (s, 4H), 2.26 (s, 3H), 1.57 (p, *J* = 5.6 Hz, 4H), 1.41 (p, *J* = 6.2 Hz, 2H). **^{13}C{^{1}H} NMR** (101 MHz, $CDCl_3$) δ / ppm = 141.3, 140.0, 136.1, 130.6, 128.4, 127.7, 127.5, 127.5, 127.3, 126.1, 81.4, 67.2, 58.8, 55.2, 26.1, 24.4, 19.6. **HRMS** (APCI) calcd. for $[C_{21}H_{27}NO+H]^+$ ($[M+H]^+$), m/z = 310.2165, found: 310.2165. **IR** (ATR, neat) $\tilde{\nu}$ / cm^{-1} = 3064, 3027, 2930, 2855, 2784, 2751, 1737, 1603, 1491, 1454, 1305, 1178, 1081, 1040, 753. **HPLC** (OJ-3, *n*-hexane:(*i*-PrOH + 1% (*v*/*v*) Et_2NH) 99:1, flow rate 0.8 mL/min, 220 nm, 25 °C) t_R = 6.520 min (99.1%), 7.066 min (0.9%). **Optical Rotation** $[\alpha]_D^{20}$ = +8.7 (*c* = 1.0, $CHCl_3$).

(*R*)-*N*,*N*-Dimethyl-2-(naphthalen-2-yl(*p*-tolyl)methoxy)ethan-1-amine (4)

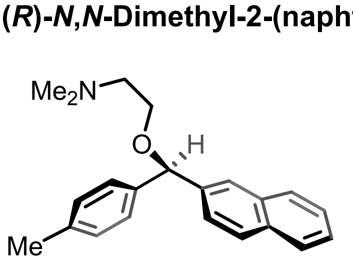

4

General procedure **E**: Benzhydrol **2b** (100 mg, 403 µmol, 90% *ee*), KOH (226 mg, 4.03 mmol, 10.0 equiv.) and 2-chloro-*N,N*-dimethylethan-1-aminium chloride (116 mg, 805 µmol, 2.00 equiv.) in dry DMSO (1.5 mL, 0.27 M) were used. Reaction time was 24 h. Purification with silica gel column chromatography (hexanes:EtOAc = 2:1 + 1% (*v*/*v*) NEt_3) afforded (*R*)-naphthoneobenodine (**4**) (104 mg, 326 µmol, 81%, 87% *ee*) as a yellow oil.

TLC R_f = 0.50 (hexanes:EtOAc = 2:1 + 1% (*v*/*v*) NEt_3). **^{1}H NMR** (300 MHz, $CDCl_3$) δ / ppm = 7.85–7.74 (m, 4H), 7.50–7.40 (m, 3H), 7.29 (s, 2H), 7.16–7.09 (m, 2H), 5.50 (s, 1H), 3.65–3.57

(m, 2H), 2.63 (t, *J* = 6.0 Hz, 2H), 2.31 (s, 3H), 2.28 (s, 6H). **^{13}C{^{1}H} NMR** (101 MHz, $CDCl_3$) δ / ppm = 139.9, 139.2, 137.2, 133.3, 133.0, 129.1, 128.3, 128.1, 127.7, 127.1, 126.1, 125.9, 125.7, 125.2, 84.1, 67.5, 59.0, 46.0, 21.2. **HRMS** (ESI) calcd. for $[C_{22}H_{25}NO+H]^+$ ($[M+H]^+$), m/z = 320.2009, found: 320.2013. **IR** (ATR, neat) $\tilde{\nu}$ / cm^{-1} = 3422, 3056, 3022, 2941, 2863, 2818, 2769, 1677, 1602, 1510, 1461, 1308, 1103, 1040, 816, 764. **HPLC** (OD-3, *n*-hexane:(*i*-PrOH:EtOH = 1:1 + 1% (*v*/*v*) Et_2NH) 99:1, flow rate 0.8 mL/min, 220 nm, 25 °C) t_R = 13.184 min (6.4%), 20.355 min (93.6%). **Optical Rotation** $[\alpha]_D^{20}$ = −9.6 (*c* = 1.0, $CHCl_3$).

(*S*)-1-(2-((4-Chlorophenyl)(phenyl)methoxy)ethyl)piperidine (6)

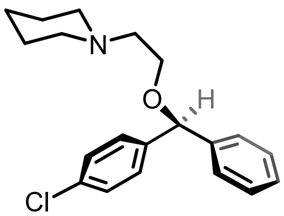

6

General procedure **E**: Benzhydrol **2d** (50.1 mg, 229 µmol, 95% *ee*), KOH (129 mg, 2.29 mmol, 10.0 equiv.) and 1-(2-chloroethyl)piperidin-1-ium chloride (84.4 mg, 459 µmol, 2.00 equiv.) in dry DMSO (2 mL, 0.1 M) were used. Reaction time was 17 h. Purification with silica gel column chromatography (hexanes:EtOAc = 9:1 + 1% (*v*/*v*) NEt_3) afforded (*S*)-cloperastine (**6**) (66.7 mg, 202 µmol, 88%, 81% *ee*) as a yellow oil.

TLC R_f = 0.24 (hexanes:EtOAc = 9:1 + 1% (*v*/*v*) NEt_3). **^{1}H NMR** (300 MHz, $CDCl_3$) δ / ppm = 7.30 (d, *J* = 4.0 Hz, 4H), 7.25 (s, 5H), 5.33 (s, 1H), 3.57 (t, *J* = 6.2 Hz, 2H), 2.63 (t, *J* = 6.2 Hz, 2H), 2.43 (t, *J* = 5.4 Hz, 4H), 1.56 (p, *J* = 5.5 Hz, 4H), 1.41 (q, *J* = 6.0 Hz, 2H). **^{13}C{^{1}H} NMR** (75 MHz, $CDCl_3$) δ / ppm = 141.9, 141.1, 133.2, 128.6, 128.6, 128.5, 127.8, 127.1, 83.3, 67.2, 58.7, 55.1, 26.1, 24.3. **HRMS** (ESI) calcd. for $[C_{20}H_{24}ClNO+H]^+$ ($[M+H]^+$), m/z = 330.1619, found: 330.1626. **IR** (ATR, neat) $\tilde{\nu}$ / cm^{-1} = 2933, 2855, 2788, 1491, 1454, 1405, 1305, 1088, 1040, 798, 757, 701. **HPLC** (OD-3, *n*-hexane:(*i*-PrOH + 1% (*v*/*v*) Et_2NH) 99:1, flow rate 0.6 mL/min, 220 nm, 25 °C) t_R = 6.823 min (90.4%), 8.137 min (9.6%). **Optical Rotation** $[\alpha]_D^{20}$ = −13.2 (*c* = 1.0, $CHCl_3$).

(*S*)-*N,N*-Dimethyl-2-(phenyl(phenyl-*d*$_5$)methoxy)ethan-1-amine (7)

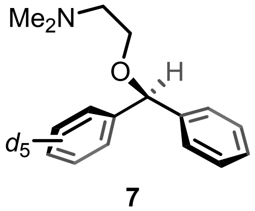

7

General procedure **E**: Benzhydrol **2e** (90.6 mg, 479 µmol), KOH (269 mg, 4.79 mmol, 10.0 equiv.) and 2-chloro-*N,N*-dimethylethan-1-aminium chloride (138 mg, 957 µmol, 2.00 equiv.) in dry DMSO (2.5 mL, 0.20 M) were used. Reaction time was 18 h. Purification with silica gel column chromatography (hexanes:EtOAc = 7:3 + 1% (*v*/*v*) NEt_3) afforded (*S*)-diphenhydramine-*d*$_5$ (**7**) (106 mg, 410 µmol, 85%) as a colorless oil. Determination of *ee*

value with chiral HPLC is impossible due to a lack of differentiation of the two enantiomers (estimation: 68% < *ee* < 82%).

TLC R_f = 0.13 (hexanes:EtOAc = 7:3 + 1% (*v*/*v*) NEt_3). **^{1}H NMR** (400 MHz, $CDCl_3$) δ / ppm = 7.37–7.28 (m, 4H), 7.26–7.21 (m, 1H), 5.37 (s, 1H), 3.57 (t, *J* = 6.0 Hz, 2H), 2.60 (t, *J* = 6.0 Hz, 2H), 2.27 (s, 6H). **^{2}H NMR** (61 MHz, $CHCl_3$) δ / ppm = 7.47–7.28 (m, 5H). **^{13}C{^{1}H} NMR** (126 MHz, $CDCl_3$) δ / ppm = 142.4, 142.3, 128.5, 128.0 (t, *J* = 23.8 Hz), 127.5, 127.1, 127.0 (t, *J* = 24.7 Hz), 126.7 (t, *J* = 23.8 Hz), 84.1, 67.7, 59.1, 46.2. **HRMS** (APCI) calcd. for $[C_{17}H_{16}D_5NO+H]^+$ ($[M+H]^+$), m/z = 261.2025, found: 261.2013. **IR** (ATR, neat) $\tilde{\nu}$ / cm^{-1} = 2941, 2863, 2818, 2769, 2273, 1495, 1454, 1349, 1309, 1141, 1100, 734, 701. **Optical Rotation** $[\alpha]_D^{20}$ = −9.5 (*c* = 1.0, $CHCl_3$).

5. Attempted synthesis of N1L protein antagonist 5

For the synthesis of N1L protein antagonist **5** we envisioned a substitution reaction of the acetate **S9**, which was obtained from Baeyer-Villiger oxidation of ketone **1c** (see above). Since purification of **S9** with silica gel column chromatography was not possible due to unintended hydrolysis, a methylation approach towards **5** was done with the crude mixture. Literature comparison suggested mild cuprates as methylation reagents as they have the highest chance of successful substitution reaction without hydrolysis. Besides, cuprates are known to react in a S_N2-type reaction,[10,11] which would avoid racemization at the benzhydryl carbon. Unfortunately, neither direct reaction (Table S1, entry 1), nor stepwise addition at lower temperatures (entry 2) led to satisfactory results. In an attempt to improve the leaving group ability of our substrate, we performed a tosylation of alcohol **2c** according to Burton *et al.*,[12] but silica gel column chromatography of **S10** again resulted in hydrolysis, only allowing for the use of the crude mixture. Methylation of **S10** with a second addition of reagents after 24 h (entry 3) led to a complex mixture without any traces of methylation product **5** but very little amounts of hydrolysis product **2c**, as was the case in all entries. These results confirm that target molecule **5** cannot be achieved under these conditions.

Table S1. Conditions for methylation attempts towards target **5** by Grieco *et al.*[10]

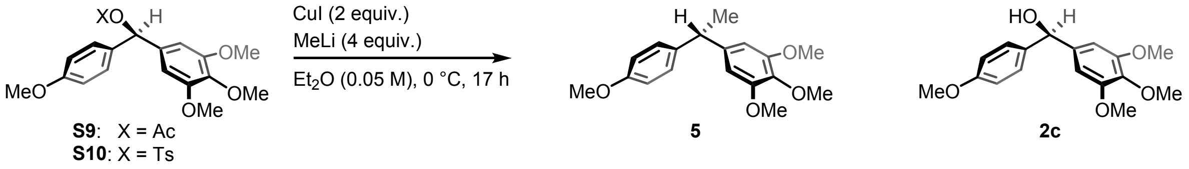

entry	X[a]	deviation from conditions stated above	results
**1**	Ac	none	0% of **5**, 8% of **2c**
**2**	Ac	2+2 equiv. CuI, 4+3 equiv. MeLi, −30–0 °C, 72 h	0% of **5**, traces of **2c**
**3**	Ts	10+10 equiv. CuI, 20+20 equiv. MeLi, −10 °C, 72 h	0% of **5**, traces of **2c**

[a] crude mixtures of **S9** and **S10** were utilized

(*R*)-(4-Methoxyphenyl)(3,4,5-trimethoxyphenyl)methyl 4-methylbenzenesulfonate (S10)

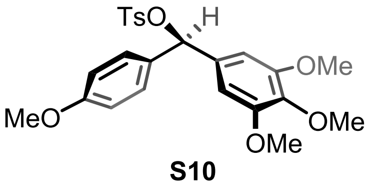

S10

Benzhydrol **2c** (81.0 mg, 260 µmol) was dissolved in DCM (5 mL, 0.05 M) and cooled to 0 °C. NEt_3 (0.30 mL, 2.2 mmol, 10 equiv.), DMAP (48 mg, 0.40 mmol, 1.5 equiv.) and TsCl (76 mg, 0.40 mmol, 1.5 equiv.) were added and the reaction mixture was stirred for 19 h at 0 °C. Another portion of TsCl (50 mg, 0.26 mmol, 1.0 equiv.) was added and the solution was stirred for another 3.5 h. Upon completion, it was diluted with DCM (10 mL), quenched with sat. aq. $NaHCO_3$ solution (10 mL) and extracted with DCM (3x 10 mL). The combined organic layer was washed with 1 M HCl (3x 5 mL), sat. aq. $NaHCO_3$ solution (10 mL), dried over $MgSO_4$, filtered, and the solvent was removed under reduced pressure to afford crude **S10** (133 mg, 160 µmol according to NMR-yield). The crude product was used without further purification.

^{1}H NMR (300 MHz, $CDCl_3$) δ / ppm = 8.17 (d, *J* = 7.8 Hz, 2H), 7.78–7.62 (m, 2H), 7.14–6.95 (m, 5H), 6.91–6.76 (m, 4H), 6.41 (s, 2H), 3.78 (s, 3H), 3.74 (d, *J* = 2.4 Hz, 3H), 3.68 (s, 6H), 3.13 (s, 6H), 2.24 (s, 3H).

Attempt towards (*S*)-1,2,3-Trimethoxy-5-(1-(4-methoxyphenyl)ethyl)benzene (5)

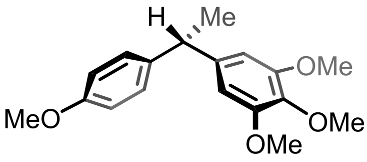

5

In a preheated Schlenk flask CuI (95 mg, 0.50 mmol, 2.0 equiv.) was suspended in dry Et_2O (3.1 mL, 0.16 M) and MeLi (1.2 M in Et_2O, 0.8 mL, 1 mmol, 4 equiv.) was added dropwise at 0 °C. The solution was stirred for 15 min. Crude **S9** (86 mg, 0.25 mmol) dissolved in dry Et_2O (1.7 mL, 0.15 M) was added dropwise, and the reaction mixture was stirred at 0 °C for 17 h. Then, it was quenched with sat. aq. NH_4Cl solution (5 mL), washed with 5% (*v*/*v*) aq. $NaHCO_3$ solution (2x 10 mL) and the phases were separated. The combined aqueous layer was extracted with Et_2O (6x 10 mL). The combined organic layer was dried over $MgSO_4$, filtered, and the solvent was removed under reduced pressure. Purification of the crude mixture by silica gel column chromatography (hexanes:Et_2O = 3:1 to 1:3, then hexanes:DCM:EtOAc = 8:4:3) did not afford the target molecule **5**, but benzhydrol **2c** (16 mg, 19 µmol, 8%) among a complex mixture.

The same procedure was applied when crude **S10** was used as the starting material.

6. Synthesis of *O*-substituted antihistamine 8

Synthesis of the *O*-substituted diarylmethane-based pharmaceutical followed the same linear synthetic route as its *O*-alkylated analogues (Scheme S2). Starting from the privileged benzhydrol intermediate **2d**, chlorination was achieved by using $SOCl_2$ at lower temperatures to maintain a high *ee* value. Unfortunately, the *ee* dropped significantly to only 16% within chloride **S11**. However, the following amination with *N*-methylpiperazine afforded the target compound **8** without any further decrease in *ee*.[13]

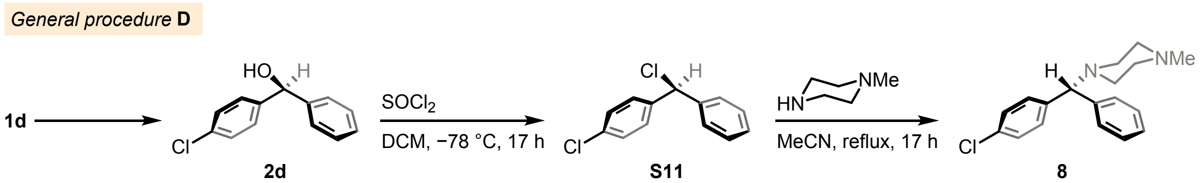

Scheme S2. Overview for synthesis of antihistamine **8**.

(*S*)-1-Chloro-4-(chloro(phenyl)methyl)benzene (S11)

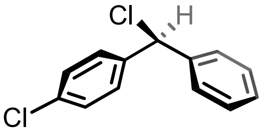

S11

In a preheated Schlenk flask under nitrogen atmosphere, benzhydrol **2d** (79.5 mg, 364 µmol, 95% *ee*) was dissolved in dry DCM (2 mL, 0.2 M) and cooled to −78 °C. $SOCl_2$ (132 µL, 1.82 mmol, 5.00 equiv.) was added dropwise and the resulting mixture was stirred at −78 °C for 17 h. Upon completion, the reaction was slowly warmed to rt and the solvent was removed under reduced pressure. Purification by silica gel column chromatography (hexanes:EtOAc = 20:1) afforded chloride **S11** (78.6 mg, 332 µmol, 91%, 16% *ee*) as a colorless oil.

TLC R_f = 0.63 (hexanes:EtOAc = 9:1). **^{1}H NMR** (400 MHz, $CDCl_3$) δ / ppm = 7.45–7.31 (m, 9H), 6.12 (s, 1H). **^{13}C{^{1}H} NMR** (101 MHz, $CDCl_3$) δ / ppm = 140.7, 139.8, 134.1, 129.3, 128.8, 128.8, 128.4, 127.8, 63.5. **HRMS** (ESI) calcd. for $[C_{13}H_{10}Cl]^+$ ($[M-Cl]^+$), m/z = 201.0471, found: 201.0470. **IR** (ATR, neat) $\tilde{\nu}$ / cm^{-1} = 3064, 3030, 1595, 1491, 1409, 1215, 1088, 1014, 846, 801, 757, 701. **HPLC** (OD-3, *n*-hexane:*i*-PrOH 99.9:0.1, flow rate 0.6 mL/min, 220 nm, 25 °C) t_R = 16.361 min (57.9%), 17.310 min (42.1%). **Optical Rotation** $[\alpha]_D^{20}$ = +3.5 (*c* = 1.0, $CHCl_3$).

(*R*)-1-((4-Chlorophenyl)(phenyl)methyl)-4-methylpiperazine (8)

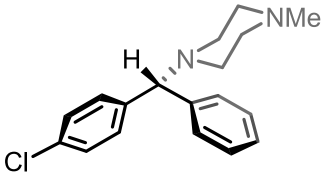

8

In a preheated Schlenk flask under nitrogen atmosphere, chloride **S11** (78.6 mg, 332 µmol, 16% *ee*) was dissolved in dry MeCN (3 mL, 0.1 M). *N*-methylpiperazine (184 µL, 1.66 mmol, 5.00 equiv.) was added to the reaction mixture, which was refluxed at 82 °C for 17 h. Upon completion, the reaction was slowly cooled to rt and the solvent was removed under reduced pressure. The crude residue was dissolved in DCM (10 mL) and washed with 1 M aq. NaOH solution (2x 10 mL). The layers were separated, and the organic layer was dried over $MgSO_4$, filtered, and the solvent was removed under reduced pressure. Purification by silica gel column chromatography (hexanes:EtOAc = 1:3 + 1% (*v*/*v*) NEt_3) afforded (*R*)-chlorcyclizine **(8)** (78.8 mg, 260 µmol, 79%, 16% *ee*) as a colorless oil.

TLC R_f = 0.13 (hexanes:EtOAc = 2:3 + 1% (*v*/*v*) NEt_3). **^{1}H NMR** (400 MHz, $CDCl_3$) δ / ppm = 7.44–7.37 (m, 4H), 7.34–7.25 (m, 4H), 7.24–7.19 (m, 1H), 4.24 (s, 1H), 2.48 (s, 8H), 2.32 (s, 3H). **^{13}C{^{1}H} NMR** (101 MHz, $CDCl_3$) δ / ppm = 142.3, 141.5, 132.6, 129.2, 128.7, 128.7, 127.9, 127.2, 75.6, 55.4, 51.9, 46.0. **HRMS** (ESI) calcd. for $[C_{18}H_{21}ClN_2+H]^+$ ($[M+H]^+$), m/z = 301.1472, found: 301.1469. **IR** (ATR, neat) $\tilde{\nu}$ / cm^{-1} = 3060, 3027, 2963, 2937, 2796, 2691, 1487, 1454, 1290, 1144, 1088, 1010, 854, 805, 760, 701. **HPLC** (OD-3, *n*-hexane:(*i*-PrOH + 1% (*v*/*v*) Et_2NH) 98:2, flow rate 0.8 mL/min, 220 nm, 25 °C) t_R = 5.883 min (57.8%), 6.344 min (42.2%). **Optical Rotation** $[\alpha]_D^{20}$ = −25.3 (*c* = 1.0, $CHCl_3$).

7. References

(1) Frank, E.; Park, S.; Harrer, E.; Flügel, J. L.; Fischer, M.; Nuernberger, P.; Rehbein, J.; Breder, A. Asymmetric Migratory Tsuji-Wacker Oxidation Enables the Enantioselective Synthesis of Hetero- and Isosteric Diarylmethanes. *J. Am. Chem. Soc.* **2024**, *146* (50), 34383–34393. DOI: 10.1021/jacs.4c09405.

(2) Park, S.; Dutta, A. K.; Allacher, C.; Abramov, A.; Dullinger, P.; Kuzmanoska, K.; Fritsch, D.; Hitzfeld, P.; Horinek, D.; Rehbein, J.; Nuernberger, P.; Gschwind, R. M.; Breder, A. Hydrogen-Bond-Modulated Nucleofugality of SeIII Species to Enable Photoredox-Catalytic Semipinacol Manifolds. *Angew. Chem. Int. Ed.* **2022**, *61* (49), e202208611. DOI: 10.1002/anie.202208611.

(3) Wilken, M.; Ortgies, S.; Breder, A.; Siewert, I. Mechanistic Studies on the Anodic Functionalization of Alkenes Catalyzed by Diselenides. *ACS Catal.* **2018**, *8* (11), 10901–10912. DOI: 10.1021/acscatal.8b01236.

(4) Tao, Z.; Gilbert, B. B.; Denmark, S. E. Catalytic, Enantioselective syn-Diamination of Alkenes. *J. Am. Chem. Soc.* **2019**, *141* (48), 19161–19170. DOI: 10.1021/jacs.9b11261.

(5) Mumford, E. M.; Hemric, B. N.; Denmark, S. E. Catalytic, Enantioselective Syn-Oxyamination of Alkenes. *J. Am. Chem. Soc.* **2021**, *143* (33), 13408–13417. DOI: 10.1021/jacs.1c06750.

(6) Satyanarayanajois, S.; Villalba, S.; Jianchao, L.; Lin, G. M. Design, synthesis, and docking studies of peptidomimetics based on HER2-herceptin binding site with potential antiproliferative activity against breast cancer cell lines. *Chem. Bio. Drug Des.* **2009**, *74* (3), 246–257. DOI: 10.1111/j.1747-0285.2009.00855.x.

(7) Dong, D.-J.; Li, H.-H.; Tian, S.-K. A highly tunable stereoselective olefination of semistabilized triphenylphosphonium ylides with N-sulfonyl imines. *J. Am. Chem. Soc.* **2010**, *132* (14), 5018–5020. DOI: 10.1021/ja910238f.

(8) Ravindar, K.; Caron, P.-Y.; Deslongchamps, P. Anionic polycyclization entry to tricycles related to quassinoids and terpenoids: a stereocontrolled total synthesis of (+)-cassaine. *J. Org. Chem.* **2014**, *79* (17), 7979–7999. DOI: 10.1021/jo501122k.

(9) Sälinger, D.; Brückner, R. The first asymmetric halogen/metal-exchange reaction: desymmetrization of alcohols with enantiotopic bromoarene substituents. *Chem. Eur. J.* **2009**, *15* (27), 6688–6703. DOI: 10.1002/chem.200802488.

(10) Grieco, P. A.; Lis, R.; Zelle, R. E.; Finn, J. Convergent, enantiospecific total synthesis of the hypocholesterolemic agent (+)-compactin. *J. Am. Chem. Soc.* **1986**, *108* (19), 5908–5919. DOI: 10.1021/ja00279a041.

(11) Gallina, C.; Ciattini, P. G. Conversion of allylic carbamates into olefins with lithium dimethylcuprate. A new formal SN2' reaction. *J. Am. Chem. Soc.* **1979**, *101* (4), 1035–1036. DOI: 10.1021/ja00498a042.

(12) Burton, J. W.; Clark, J. S.; Derrer, S.; Stork, T. C.; Bendall, J. G.; Holmes, A. B. Synthesis of Medium Ring Ethers. 5. The Synthesis of (+)-Laurencin. *J. Am. Chem. Soc.* **1997**, *119* (32), 7483–7498. DOI: 10.1021/ja9709132.

(13) Ana, G.; Kelly, P. M.; Malebari, A. M.; Noorani, S.; Nathwani, S. M.; Twamley, B.; Fayne, D.; O'Boyle, N. M.; Zisterer, D. M.; Pimentel, E. F.; Endringer, D. C.; Meegan, M. J. Synthesis and Biological Evaluation of 1-(Diarylmethyl)-1H-1,2,4-triazoles and 1-(Diarylmethyl)-1H-imidazoles as a Novel Class of Anti-Mitotic Agent for Activity in Breast Cancer. *Pharmaceuticals* **2021**, *14* (2), 169. DOI: 10.3390/ph14020169.

8. NMR and IR spectra

^{1}H NMR (400 MHz, $CDCl_3$) of **S3**

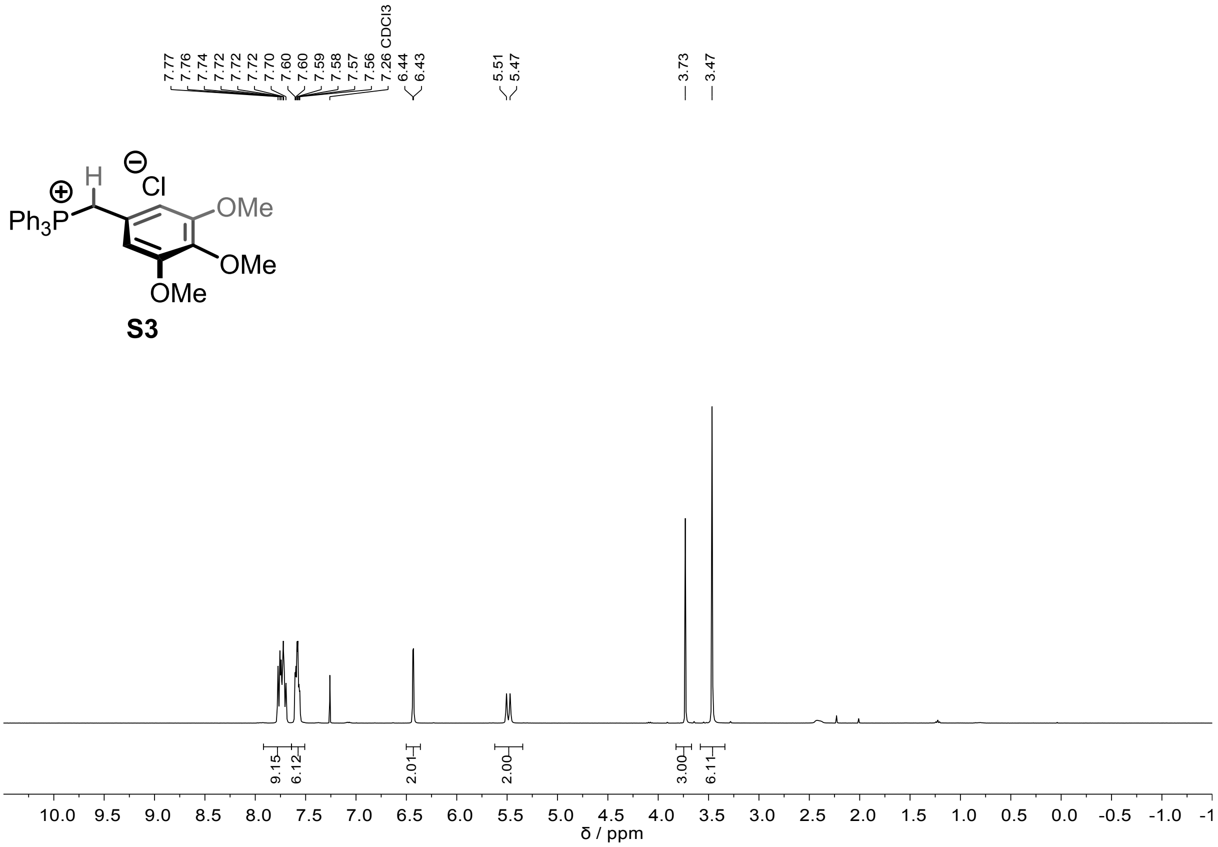

$^{13}C\{^{1}H\}$ NMR (101 MHz, $CDCl_3$) of **S3**

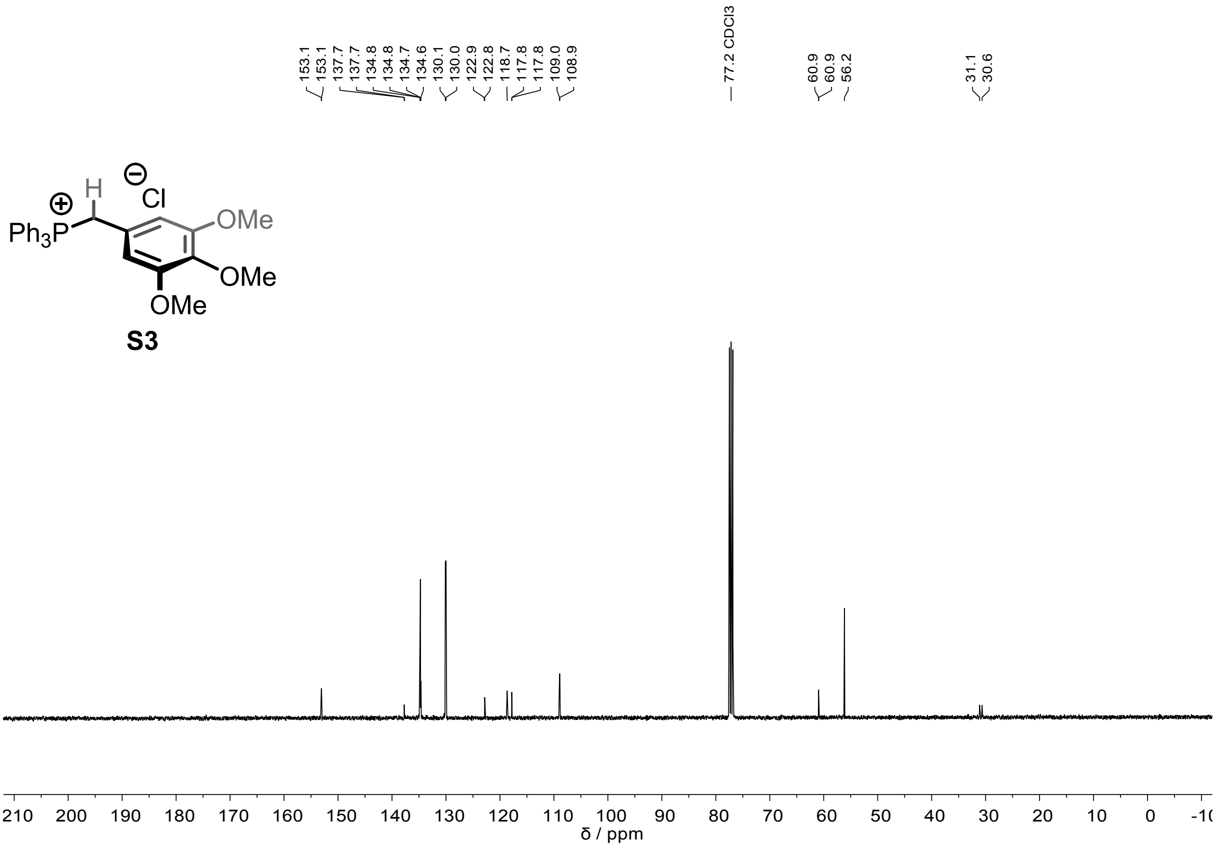

$^{31}P\{^{1}H\}$ NMR (162 MHz, $CDCl_3$) of **S3**

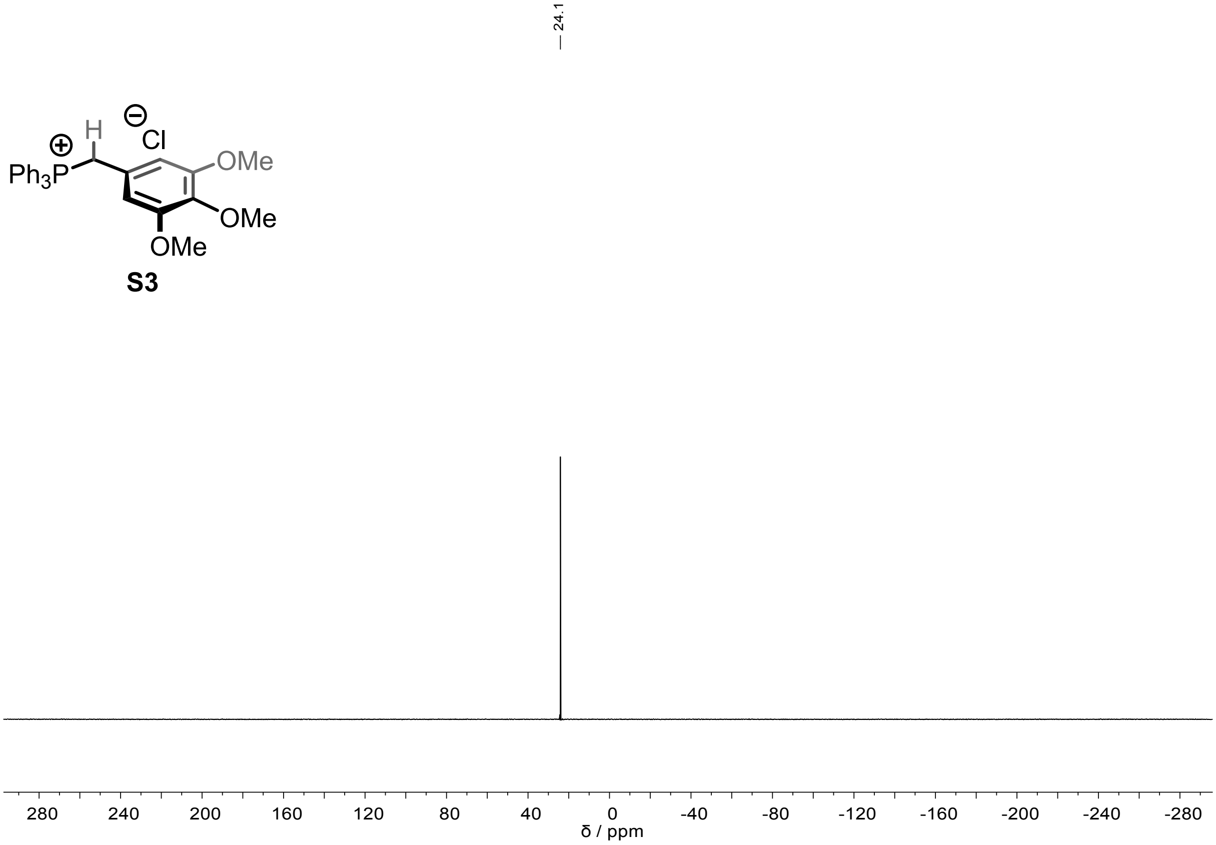

IR (ATR, neat) of **S3**

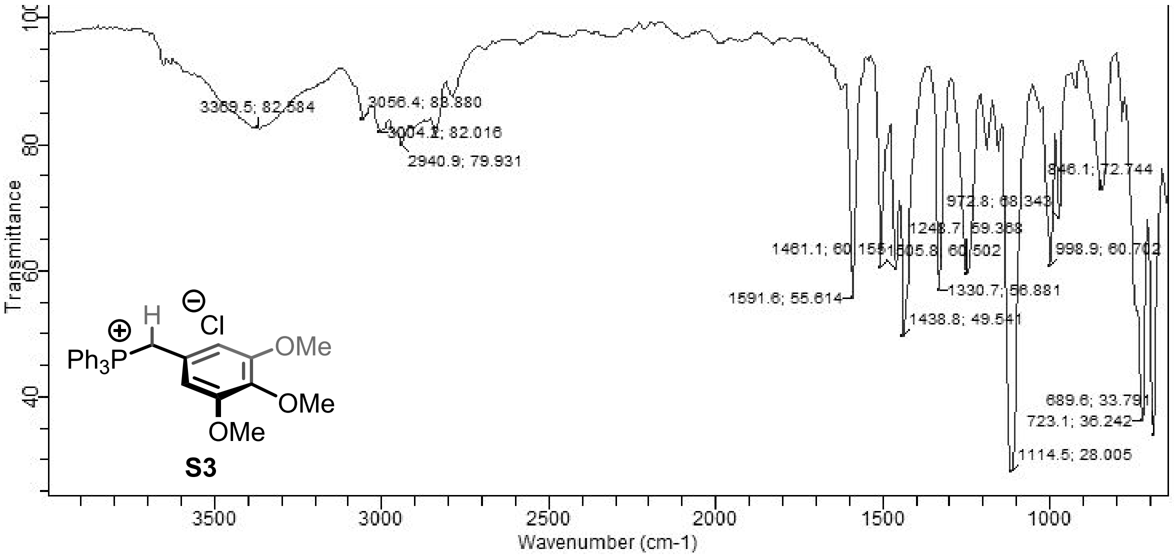

^{1}H NMR (400 MHz, $CDCl_3$) of **S6** (*E*:*Z* = 49:51)

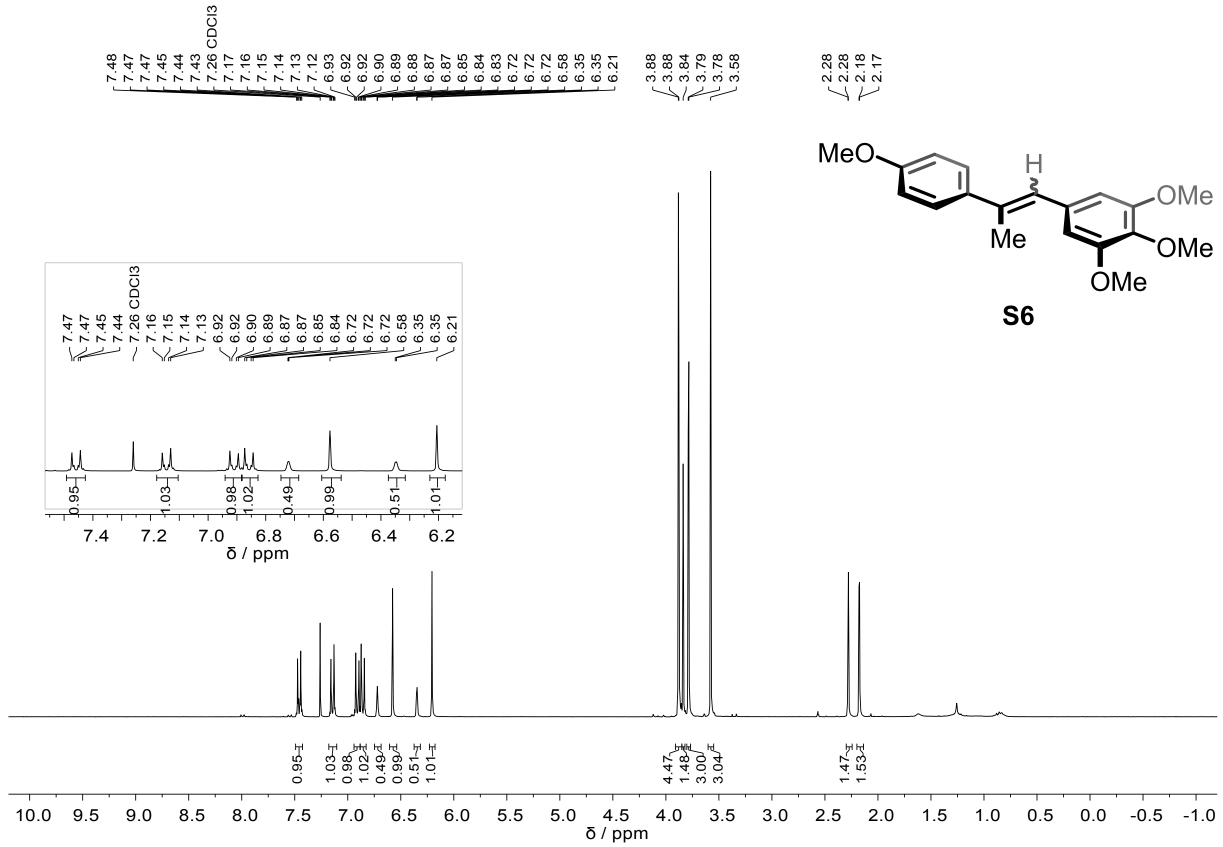

^{1}H NMR (400 MHz, $CDCl_3$) of (*E*)-**S6**

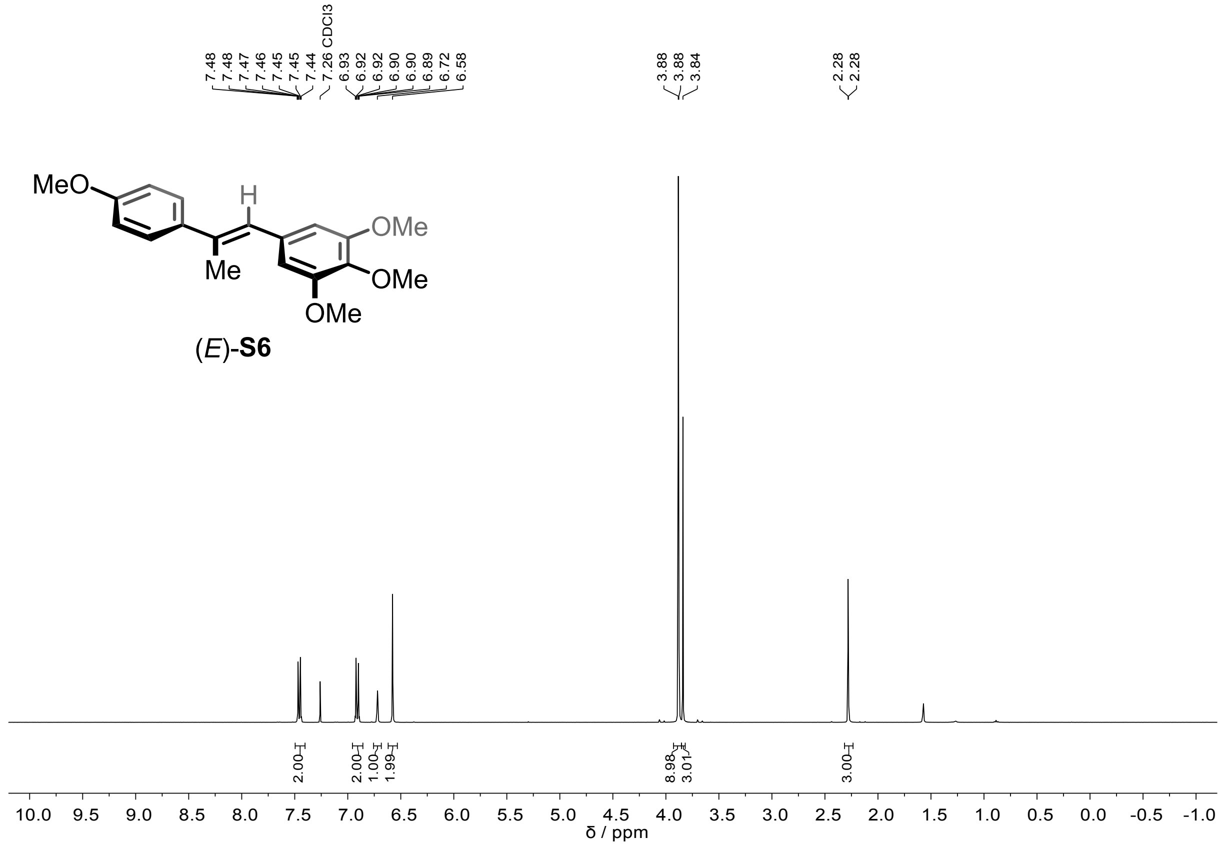

$^{13}C\{^{1}H\}$ NMR (101 MHz, $CDCl_3$) of **S6** (*E*:*Z* = 49:51)

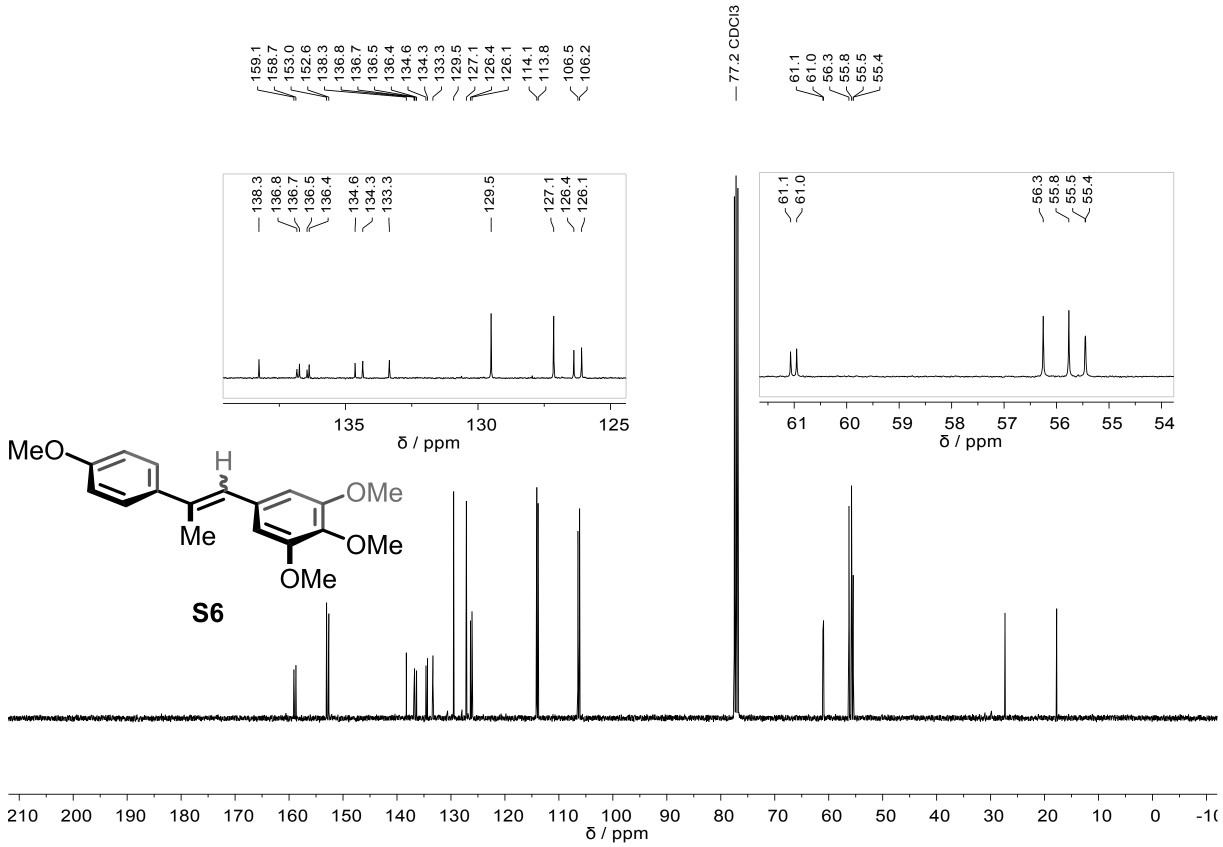

$^{13}C\{^{1}H\}$ NMR (101 MHz, $CDCl_3$) of (*E*)-**S6**

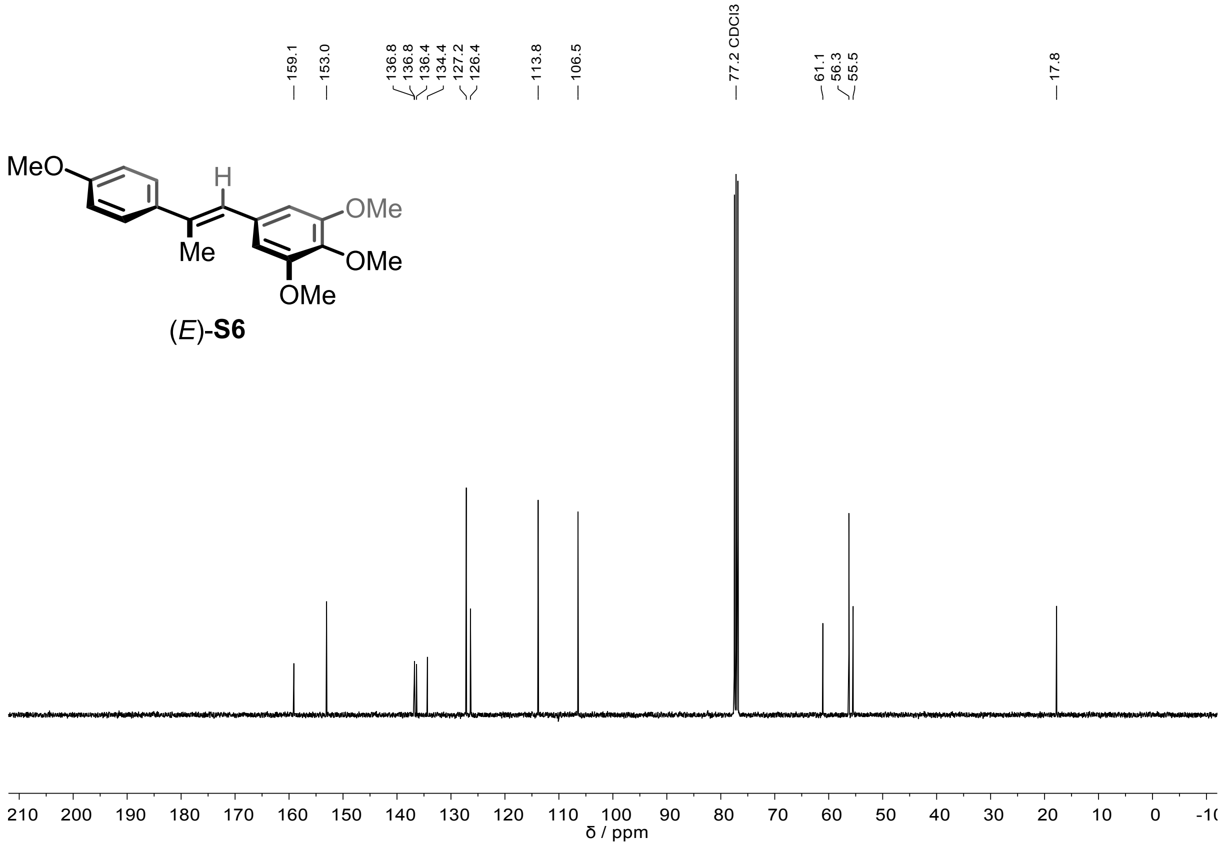

IR (ATR, neat) of **S6**

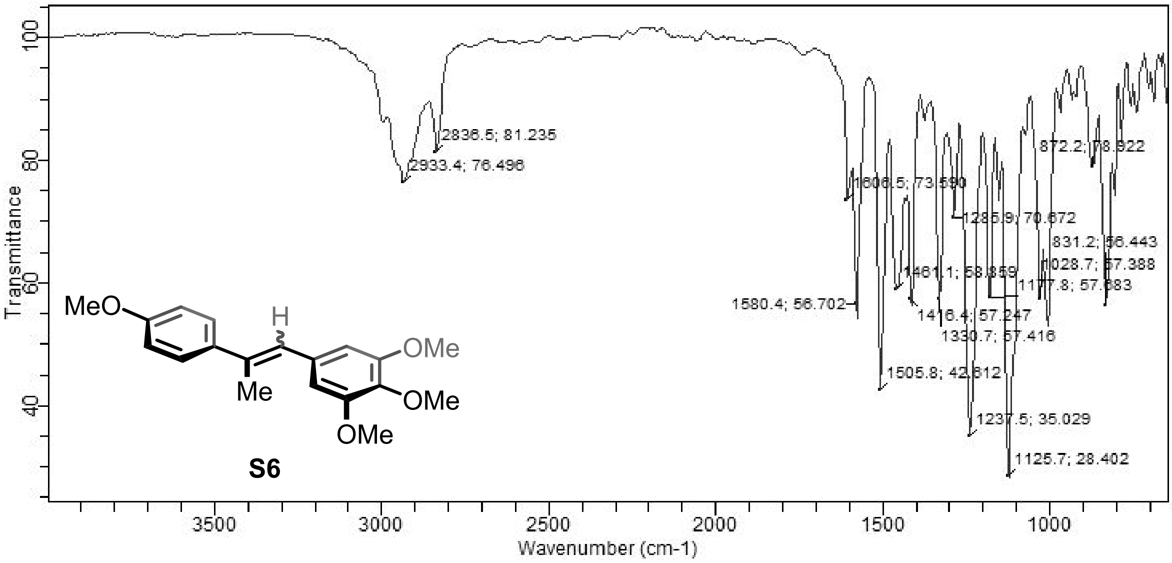

S6

^{1}H NMR (400 MHz, $CDCl_3$) of **S7** (*E*:*Z* = 57:43)

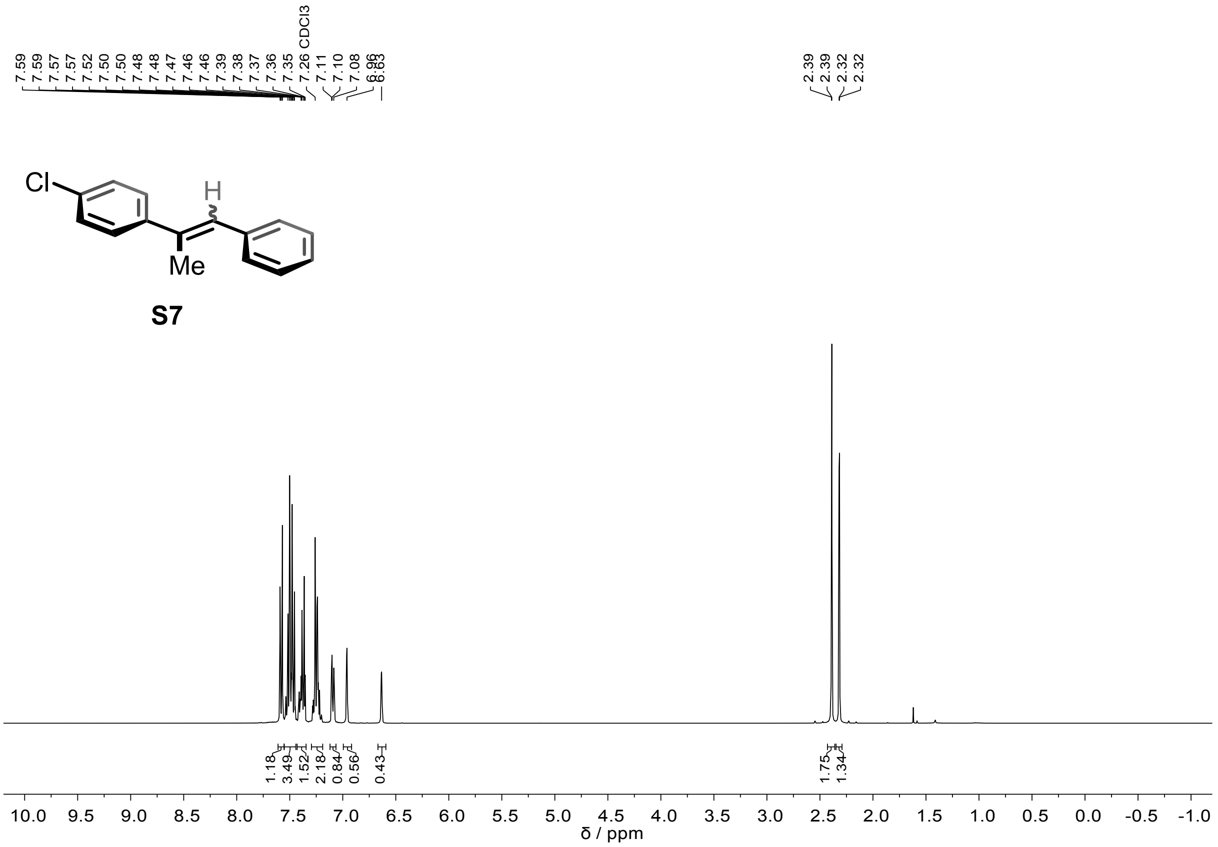

$^{13}C\{^{1}H\}$ NMR (101 MHz, $CDCl_3$) of **S7** (*E*:*Z* = 57:43)

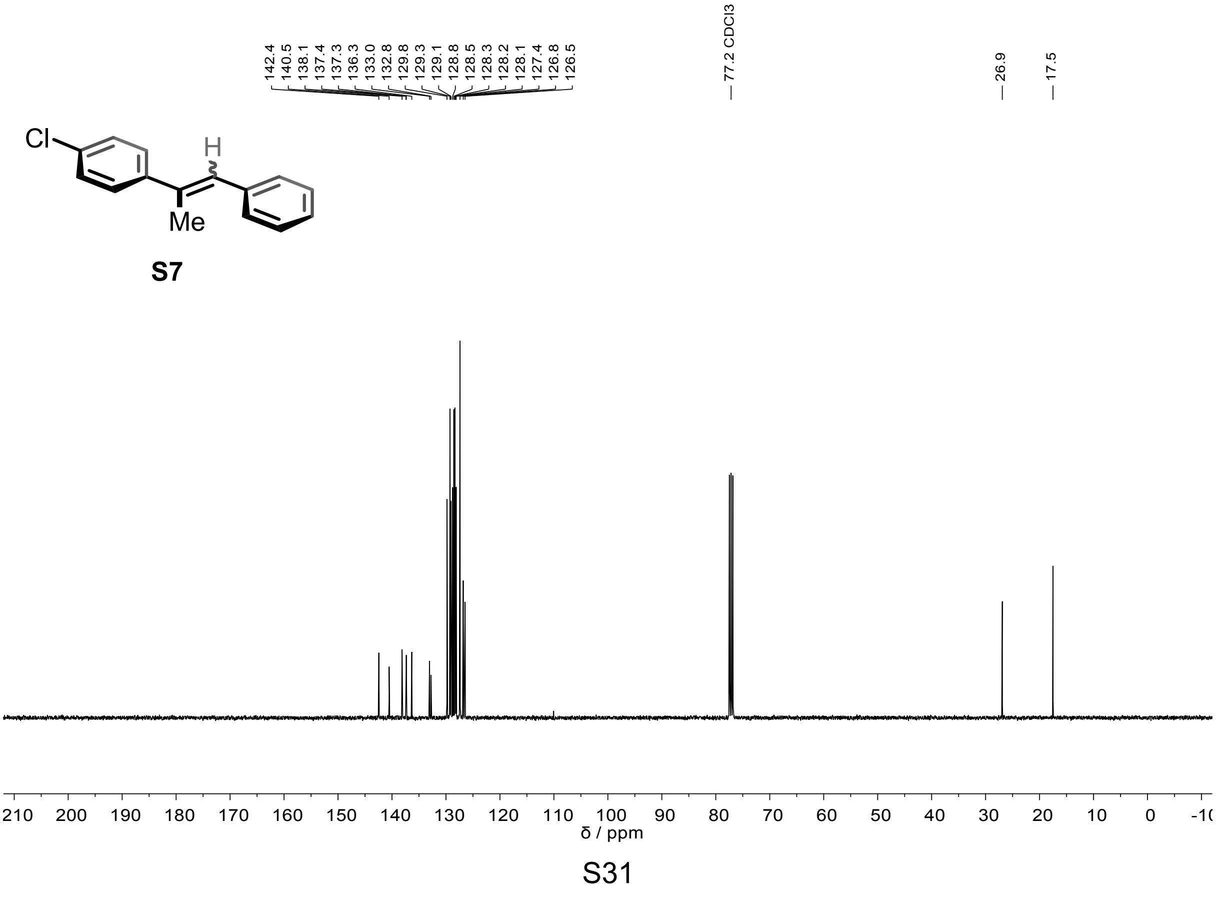

IR (ATR, neat) of **S7**

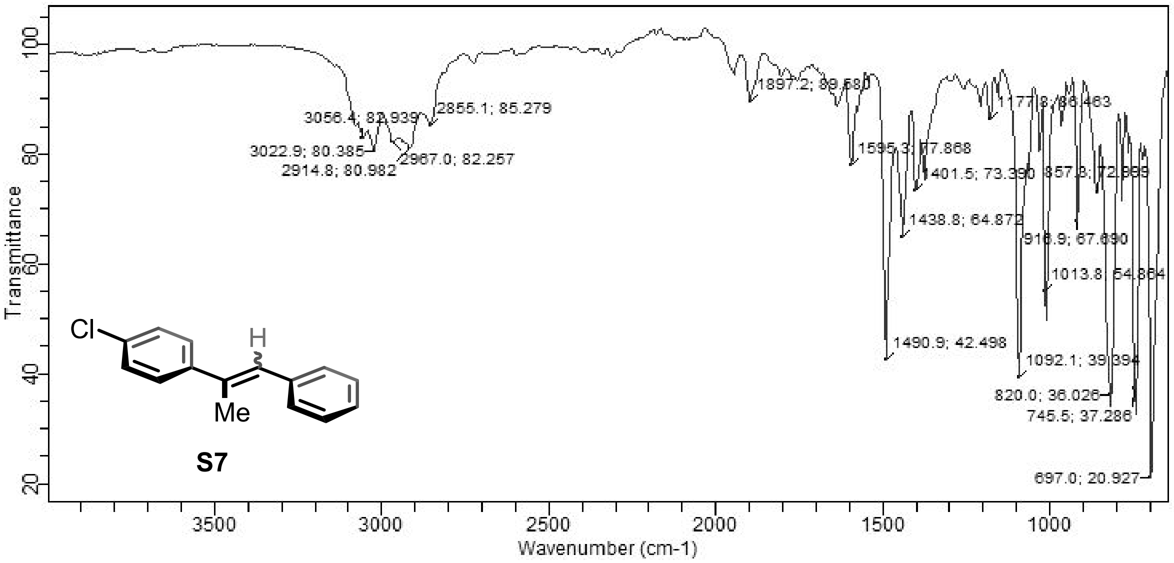

^{1}H NMR (400 MHz, $CDCl_3$) of **1c**

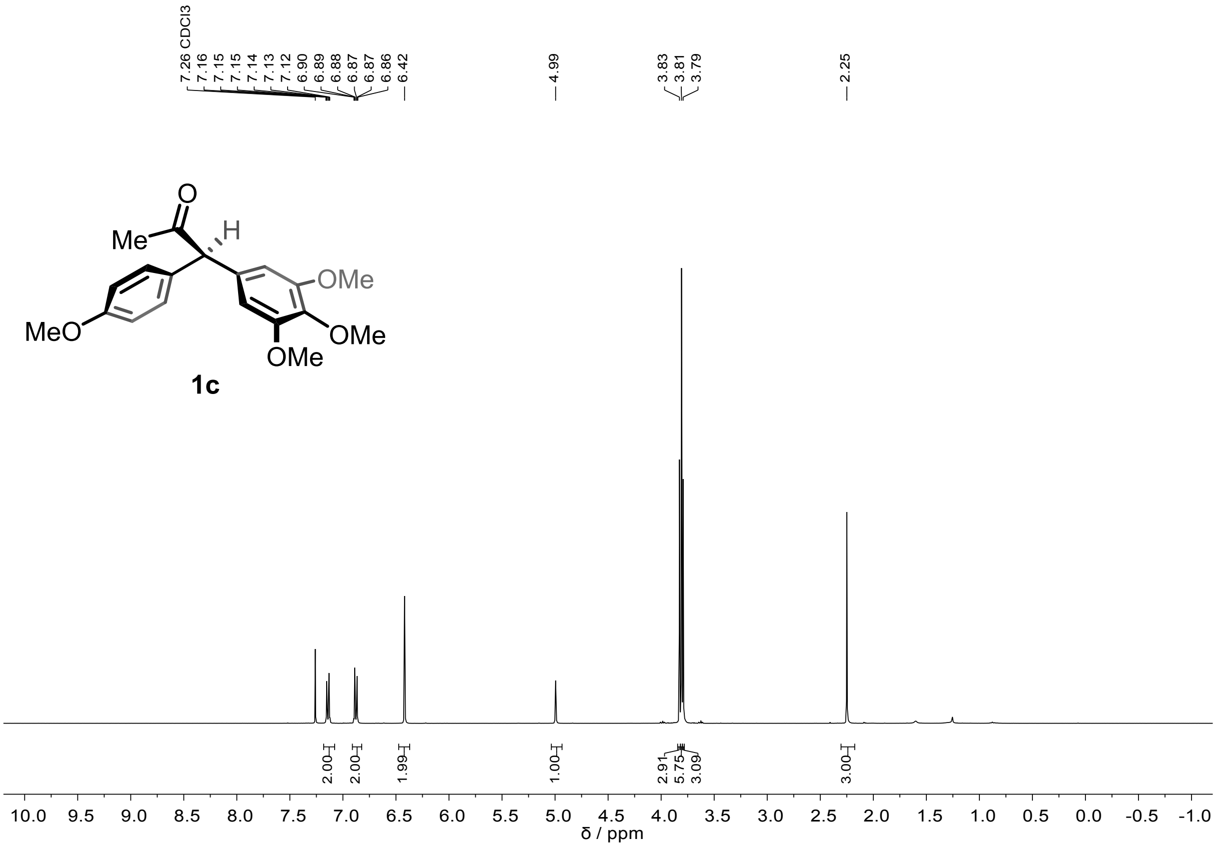

$^{13}C\{^{1}H\}$ NMR (101 MHz, $CDCl_3$) of **1c**

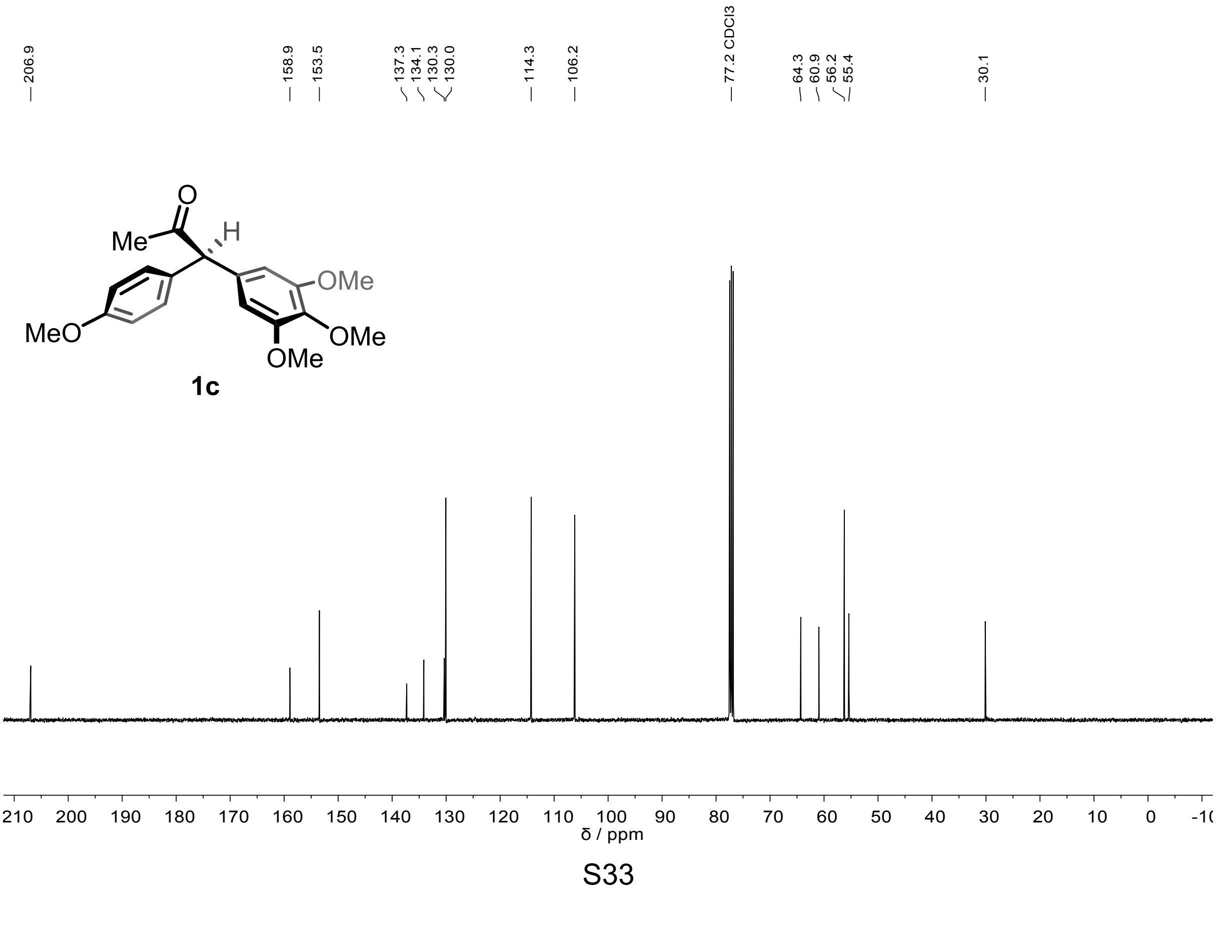

IR (ATR, neat) of **1c**

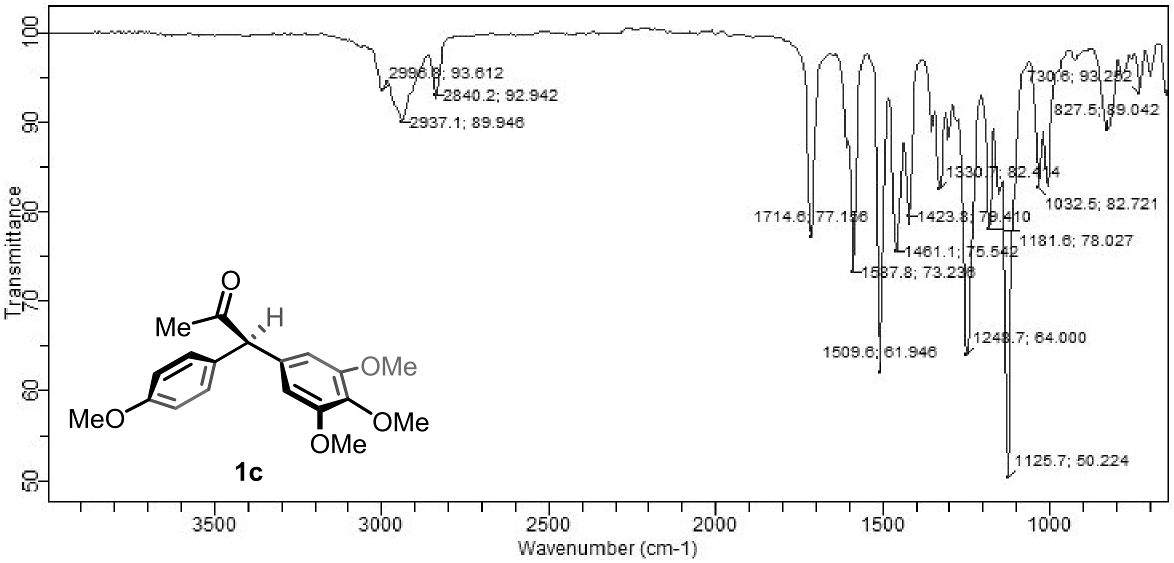

^{1}H NMR (400 MHz, $CDCl_3$) of **1d**

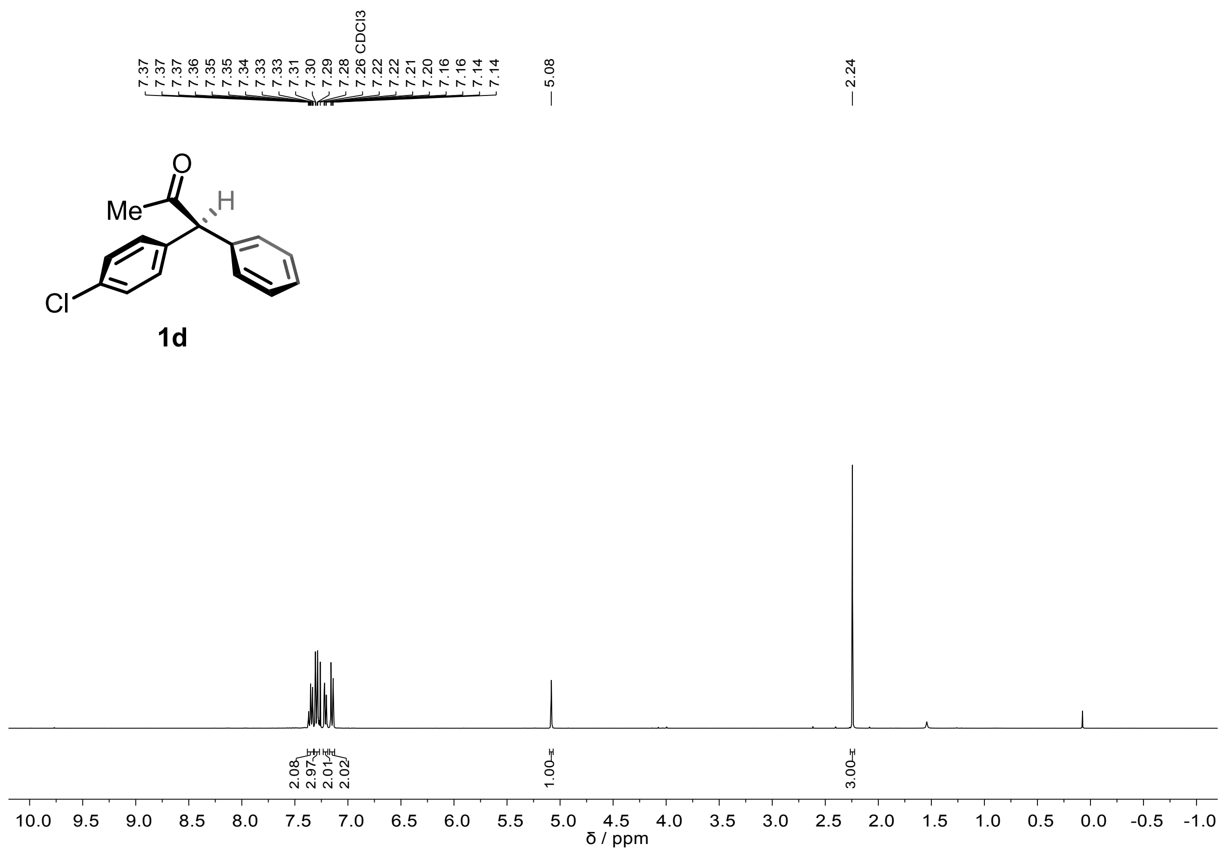

$^{13}C\{^{1}H\}$ NMR (101 MHz, $CDCl_3$) of **1d**

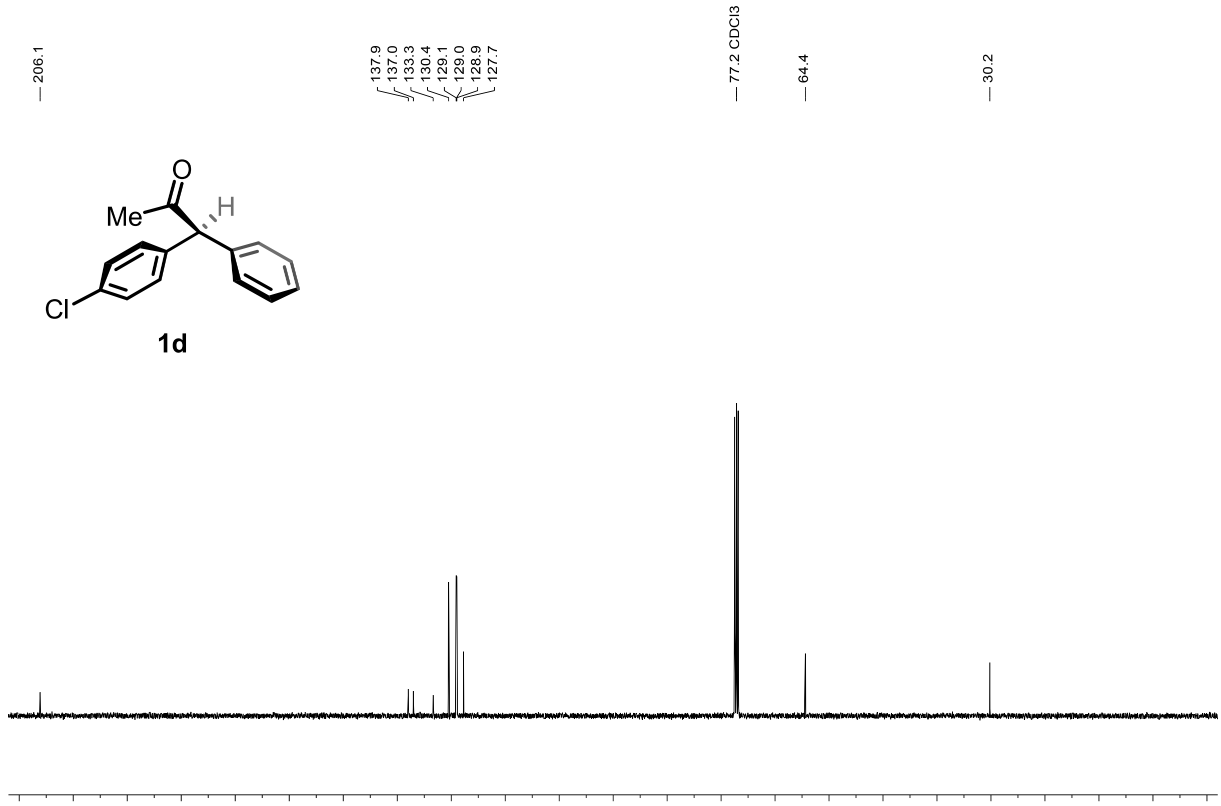

IR (ATR, neat) of **1d**

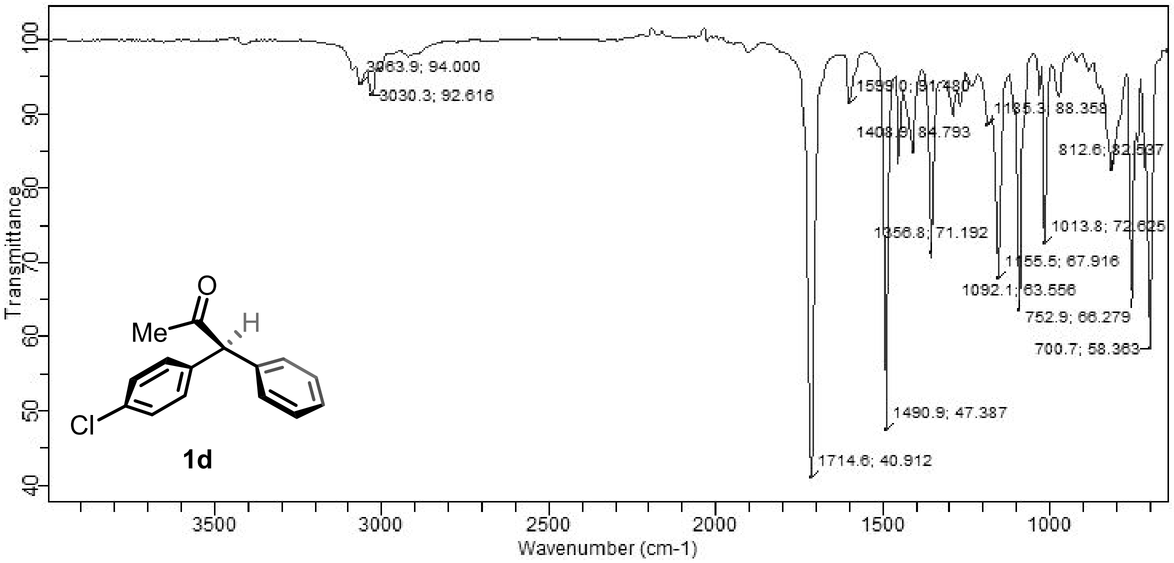

^{1}H NMR (400 MHz, $CDCl_3$) of **2a**

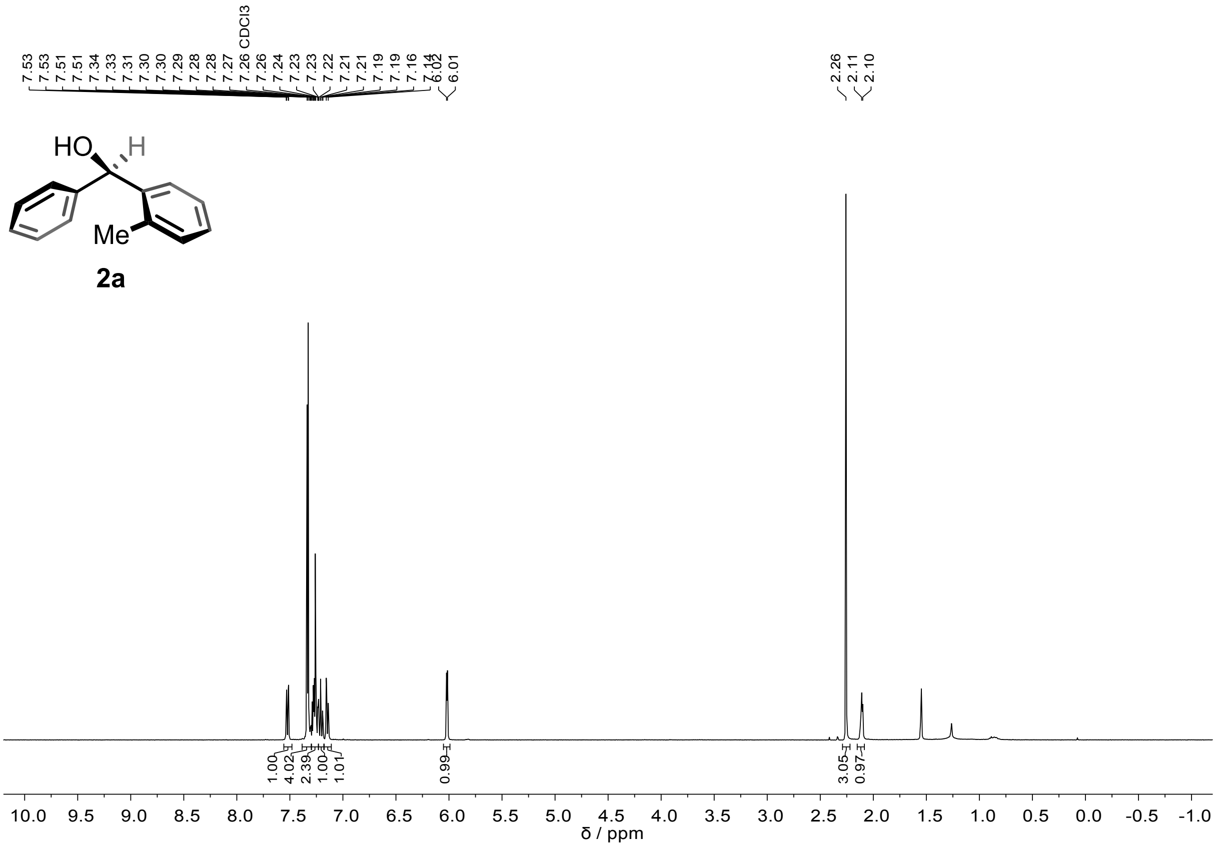

$^{13}C\{^{1}H\}$ NMR (101 MHz, $CDCl_3$) of **2a**

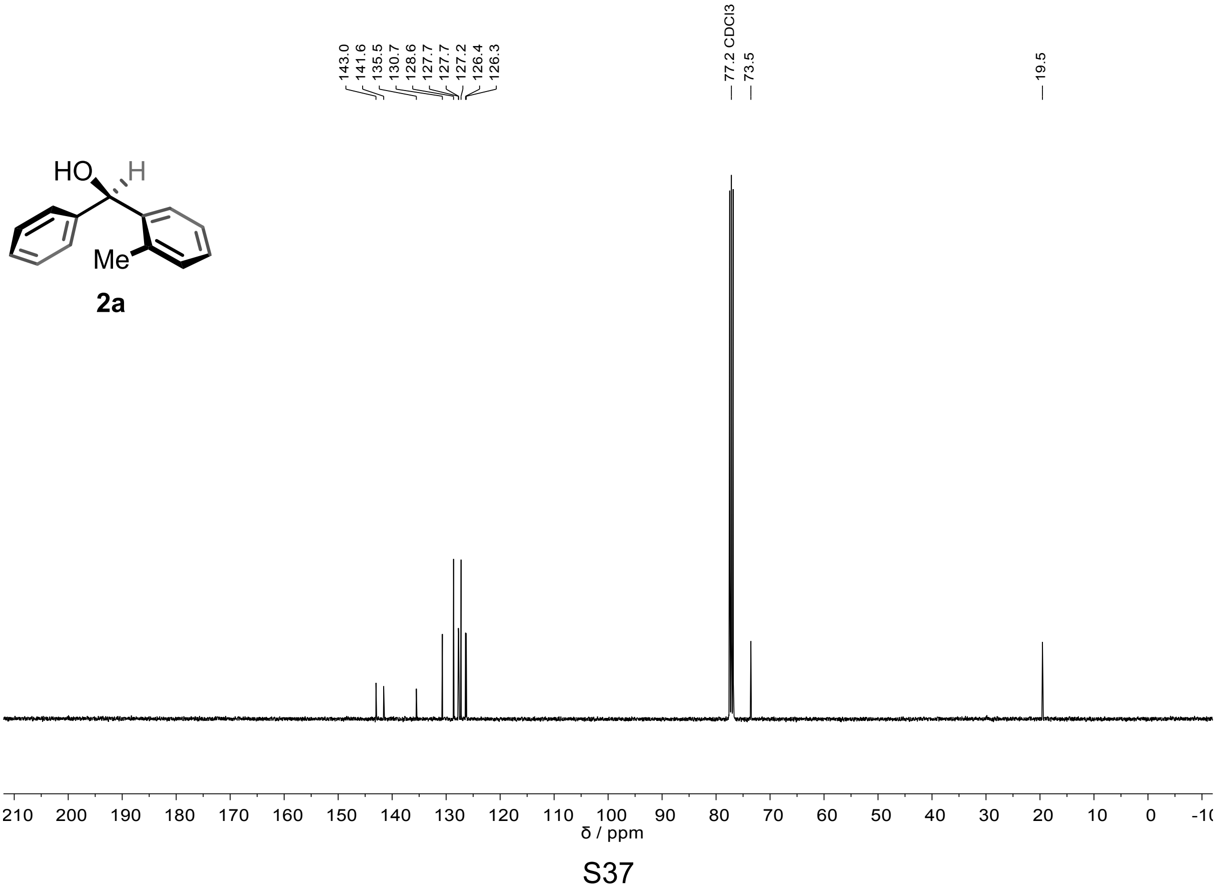

IR (ATR, neat) of **2a**

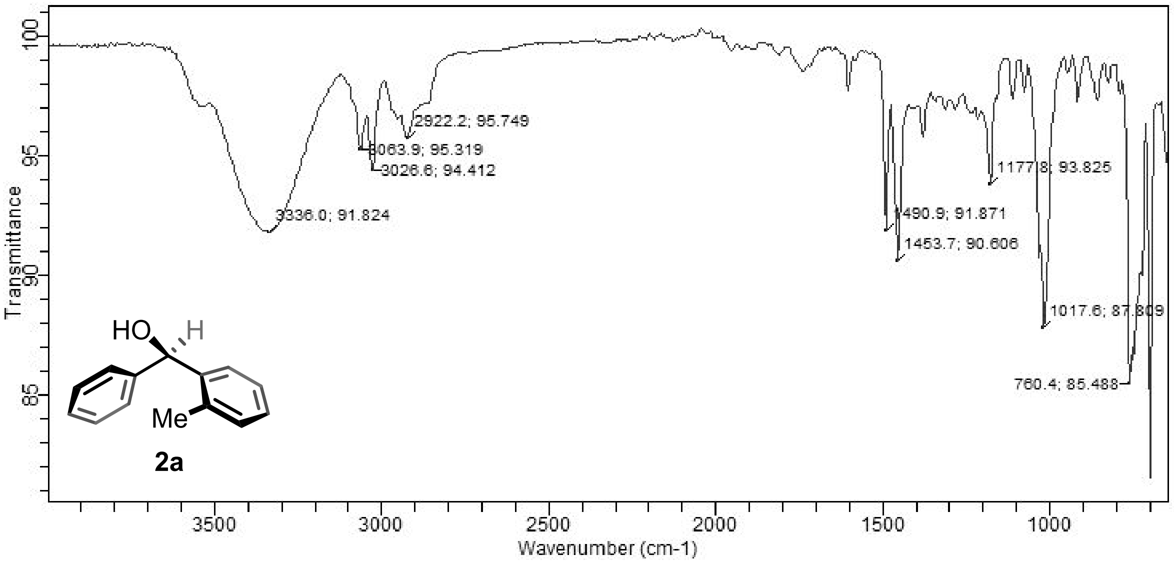

^{1}H NMR (300 MHz, $CDCl_3$) of **2b**

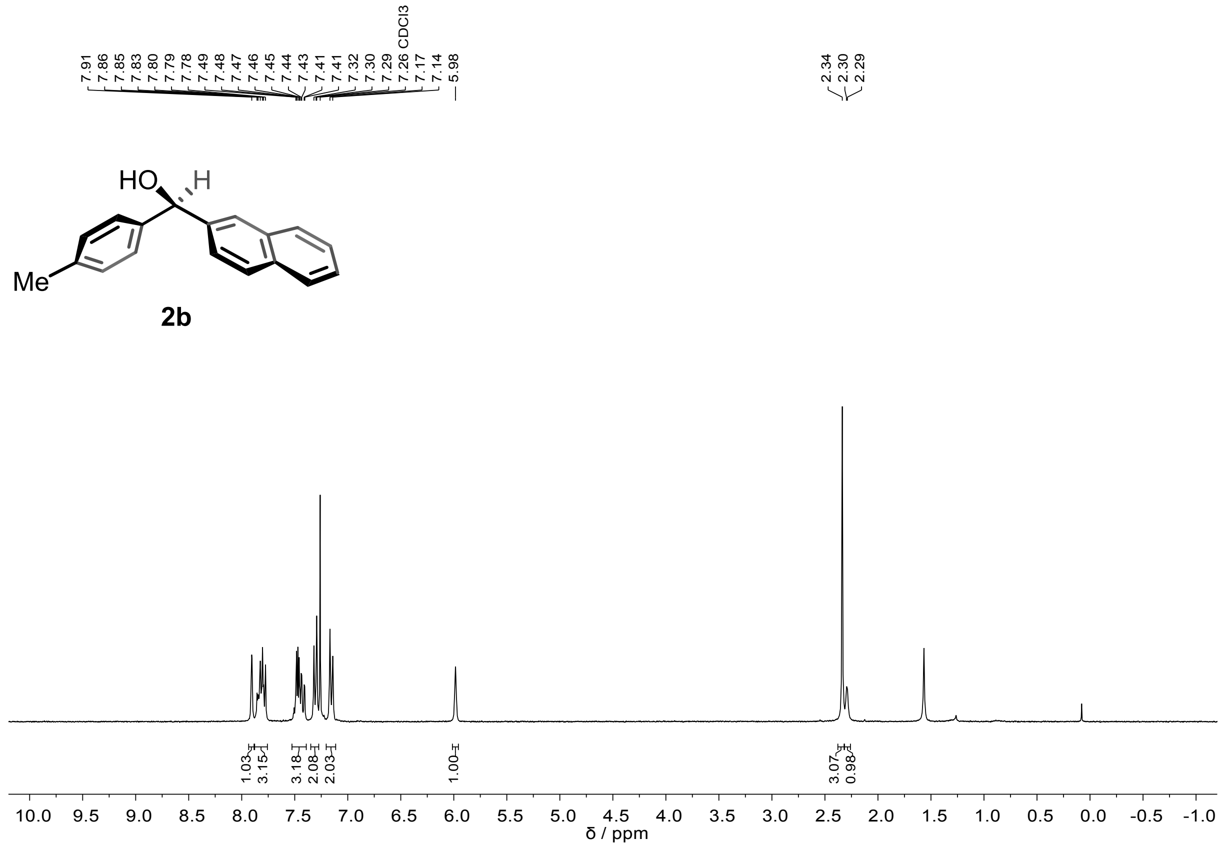

$^{13}C\{^{1}H\}$ NMR (75 MHz, $CDCl_3$) of **2b**

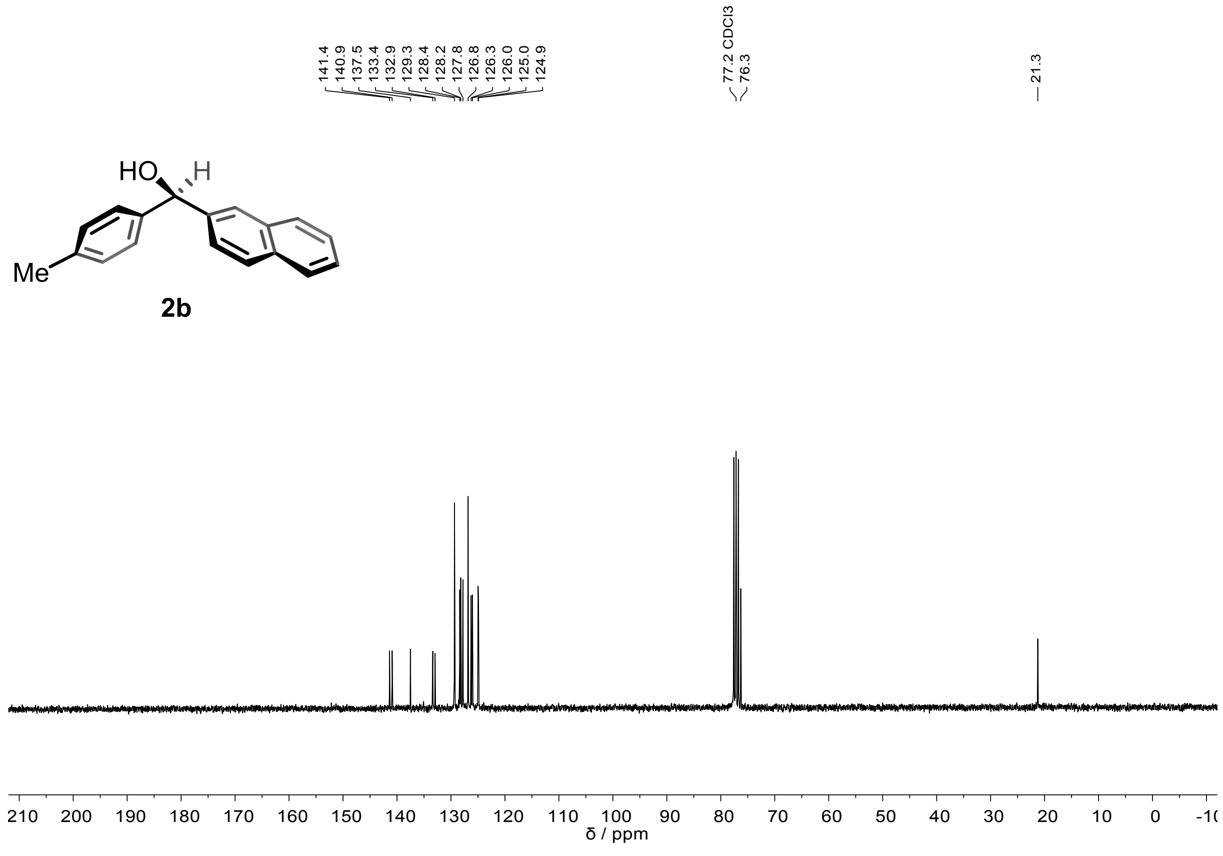

IR (ATR, neat) of **2b**

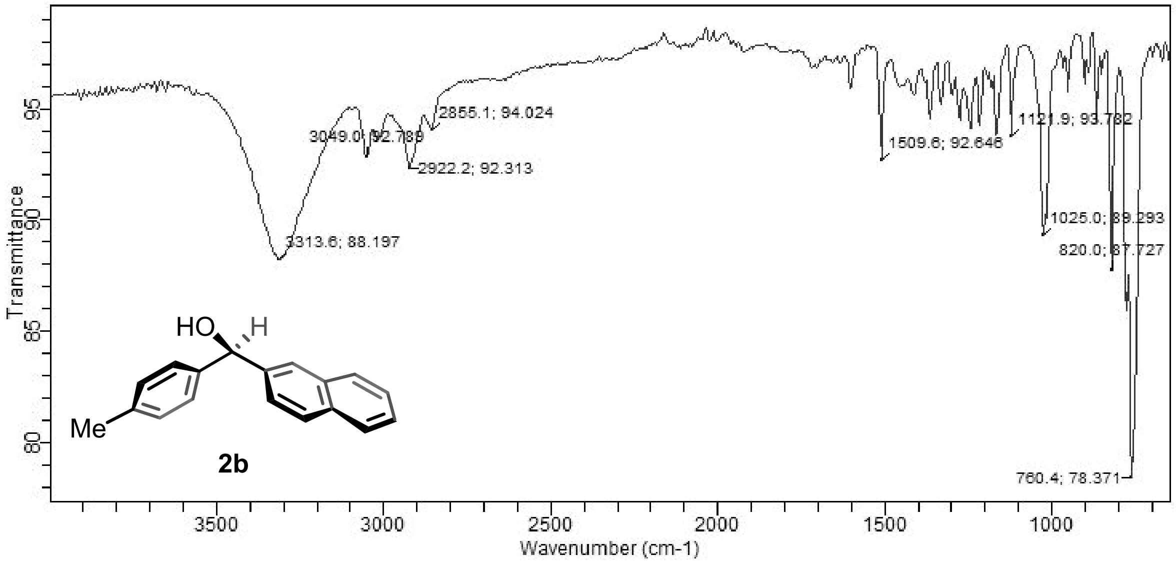

^{1}H NMR (400 MHz, $CDCl_3$) of **2c**

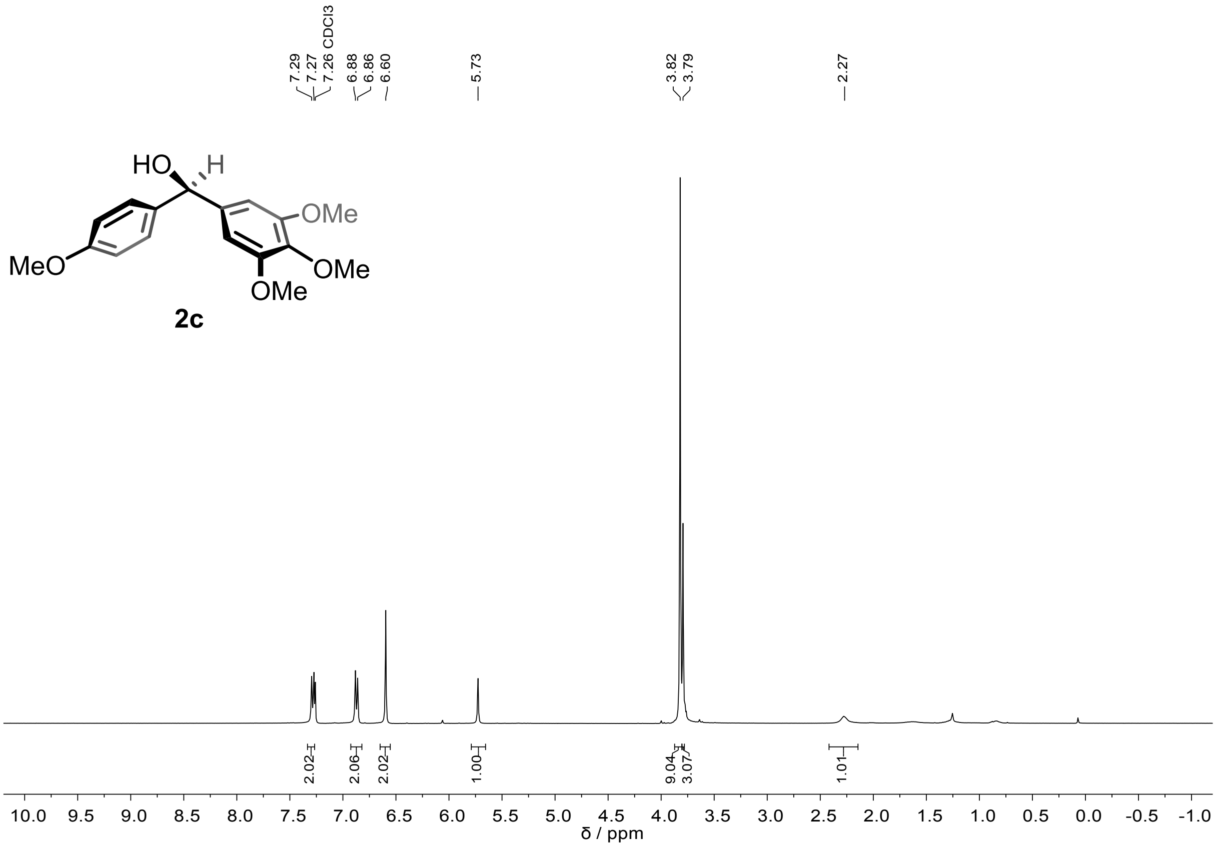

$^{13}C\{^1H\}$ NMR (101 MHz, $CDCl_3$) of **2c**

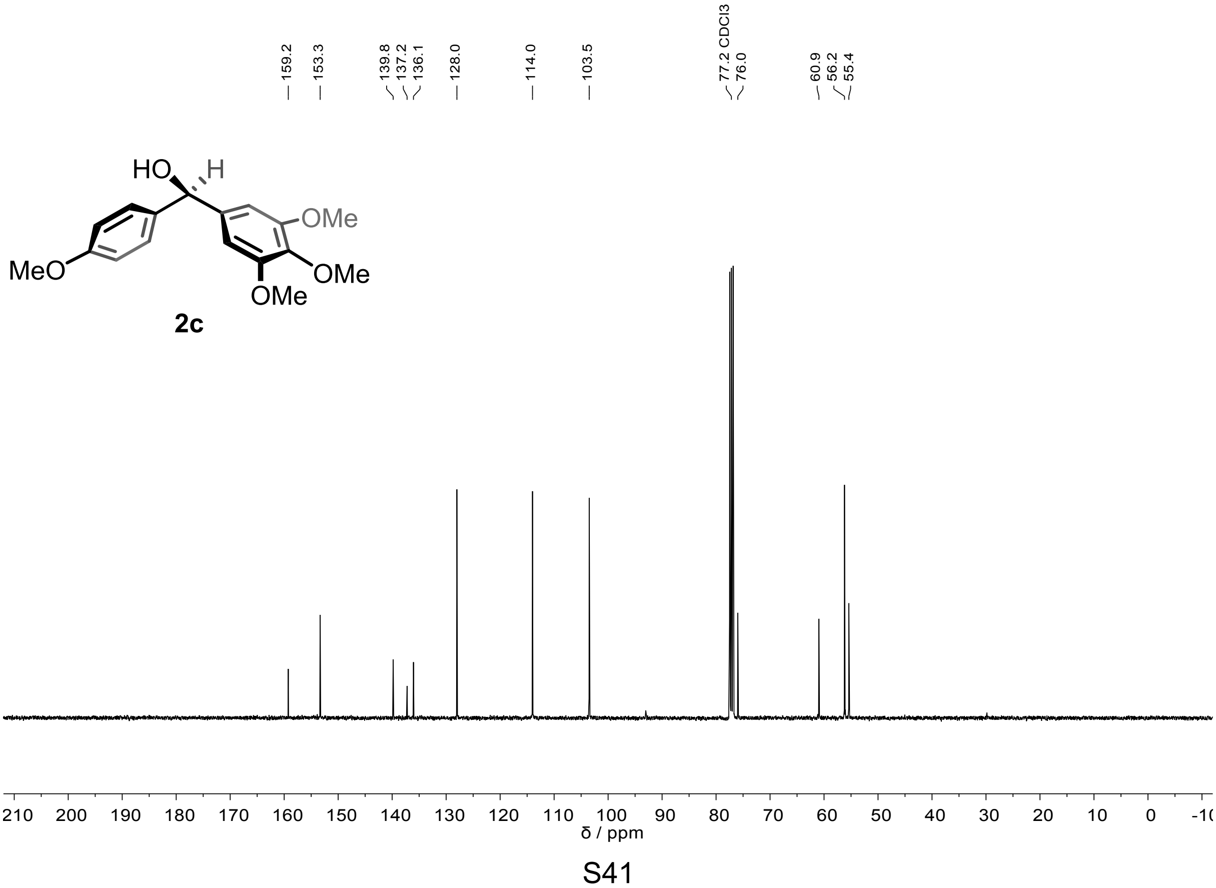

IR (ATR, neat) of **2c**

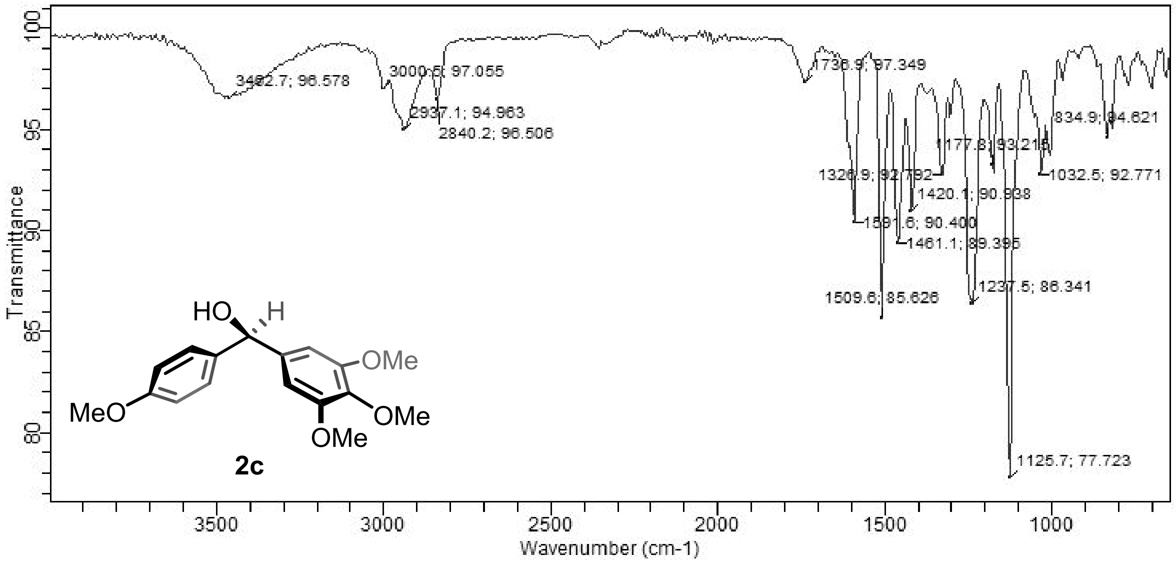

^{1}H NMR (400 MHz, $CDCl_3$) of **2d**

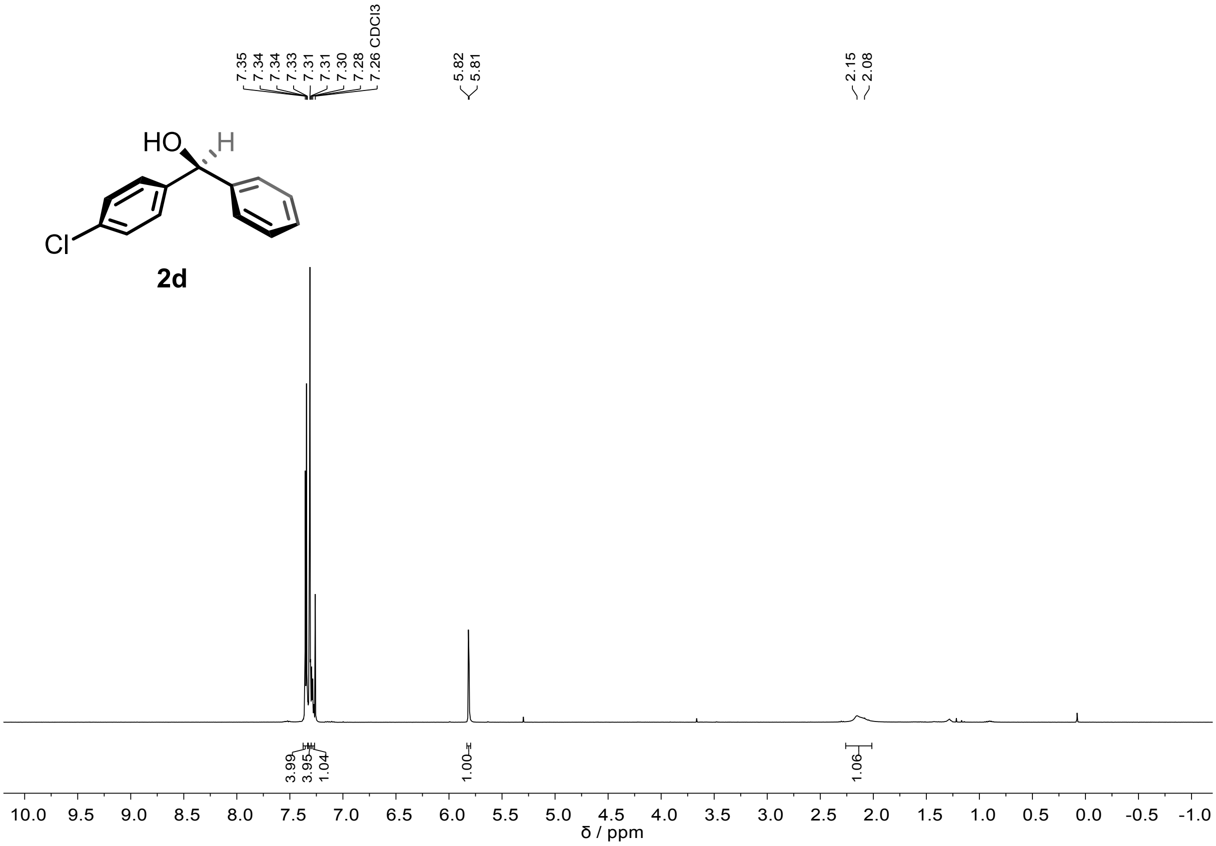

$^{13}C\{^1H\}$ NMR (101 MHz, $CDCl_3$) of **2d**

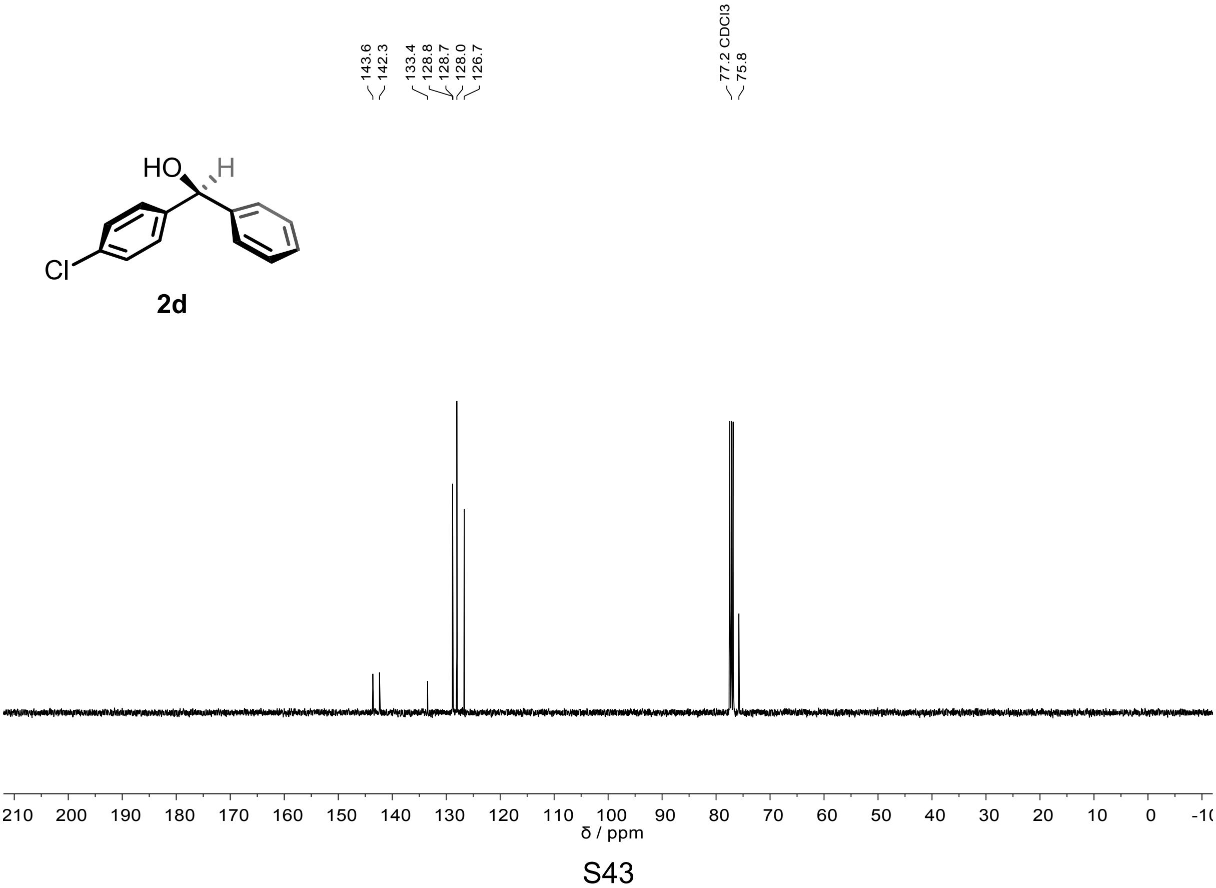

IR (ATR, neat) of **2d**

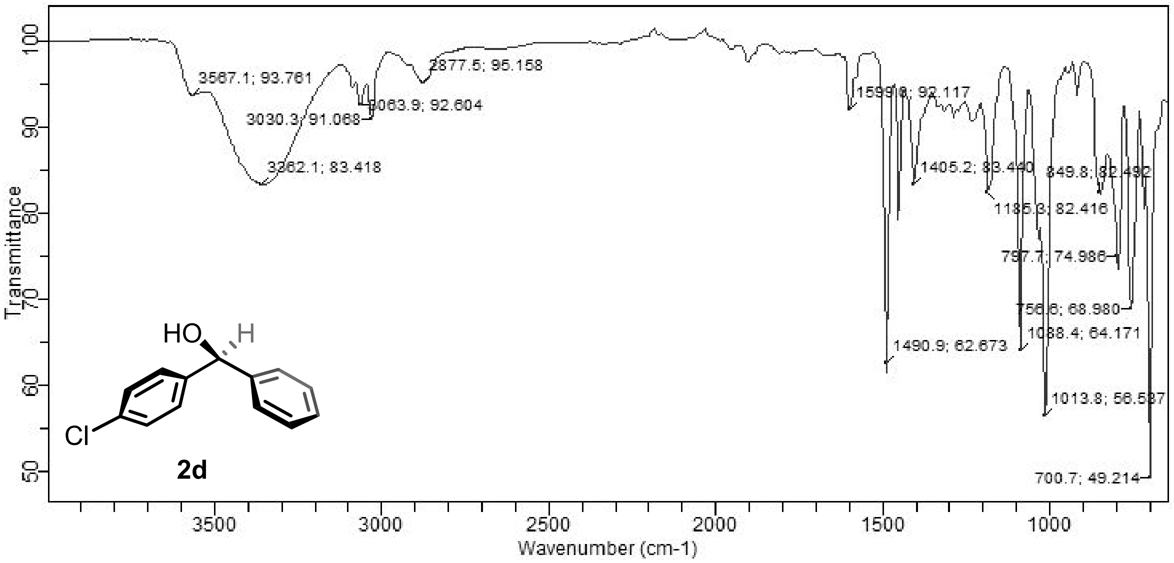

^{1}H NMR (400 MHz, $CDCl_3$) of **2e**

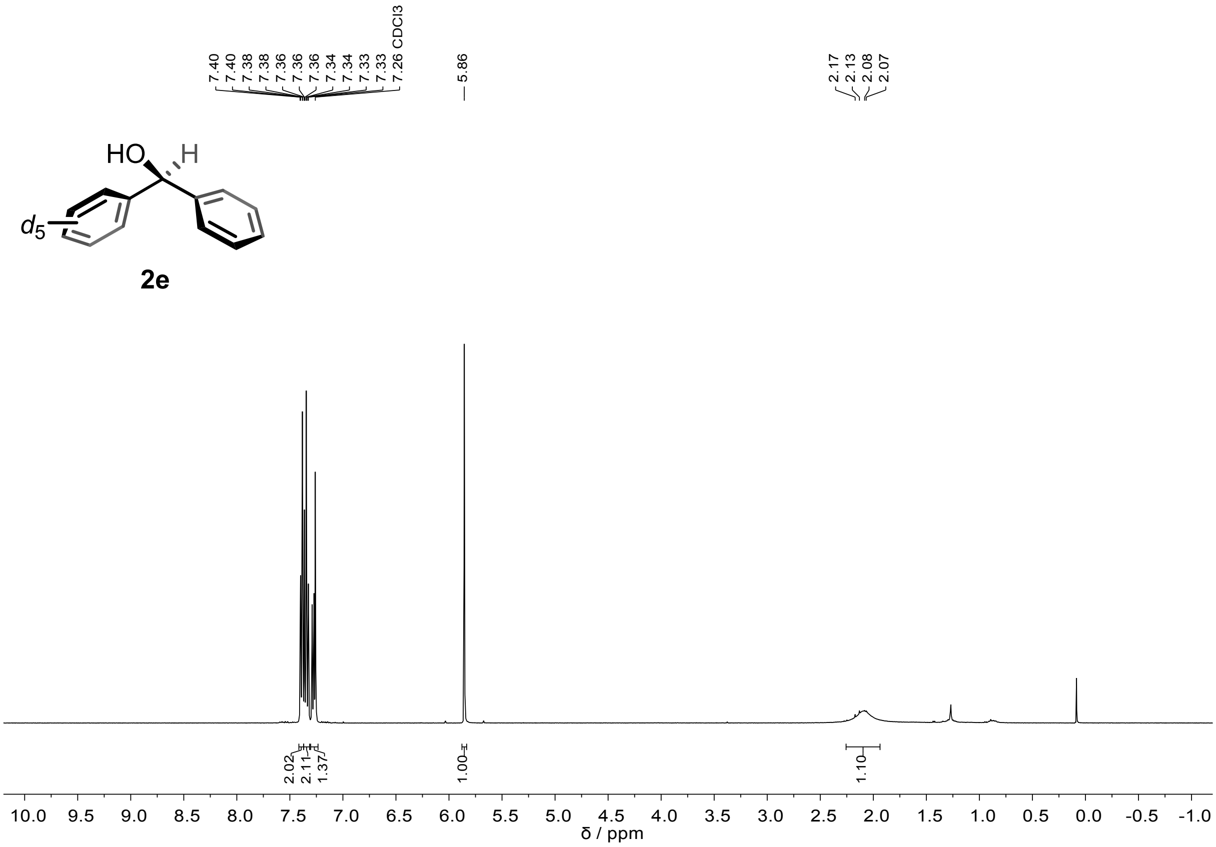

$^{13}C\{^1H\}$ NMR (126 MHz, $CDCl_3$) of **2e**

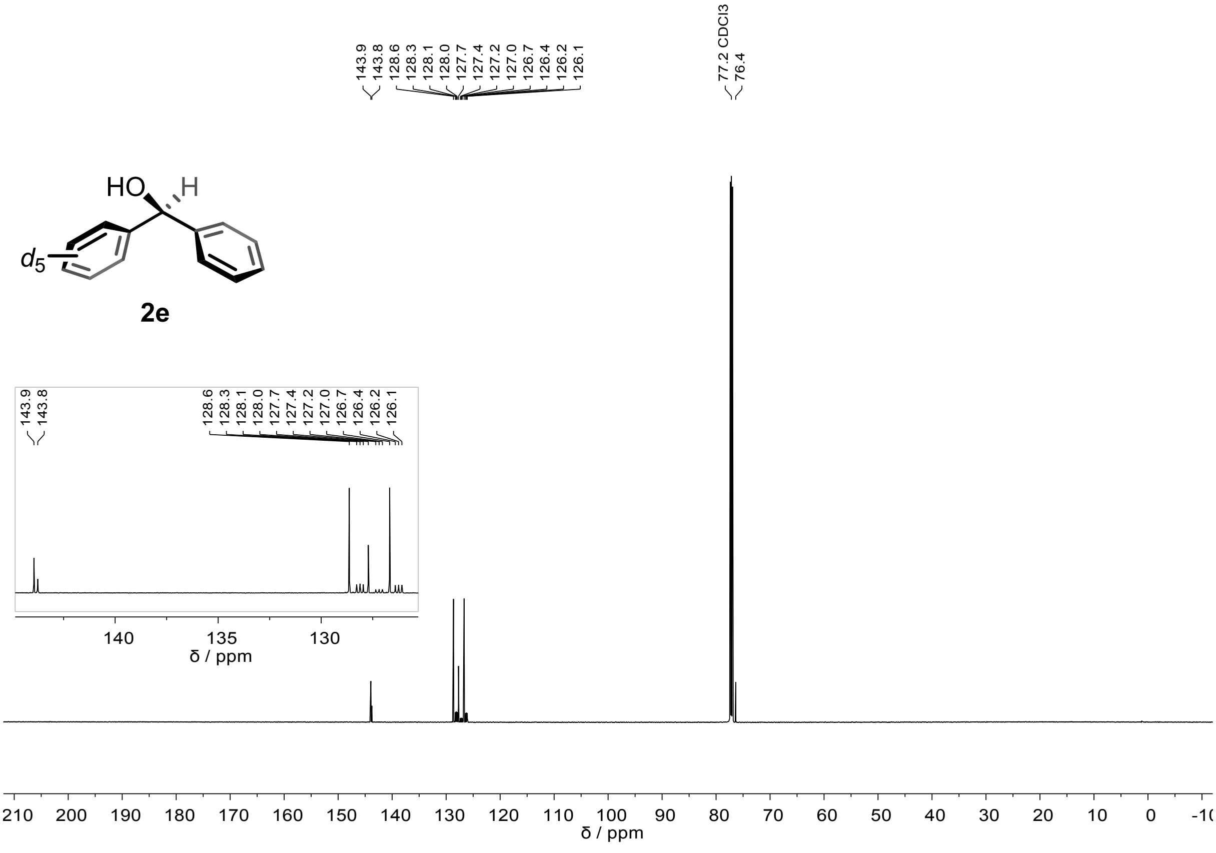

^{2}H NMR (92 MHz, $CHCl_3$) of **2e**

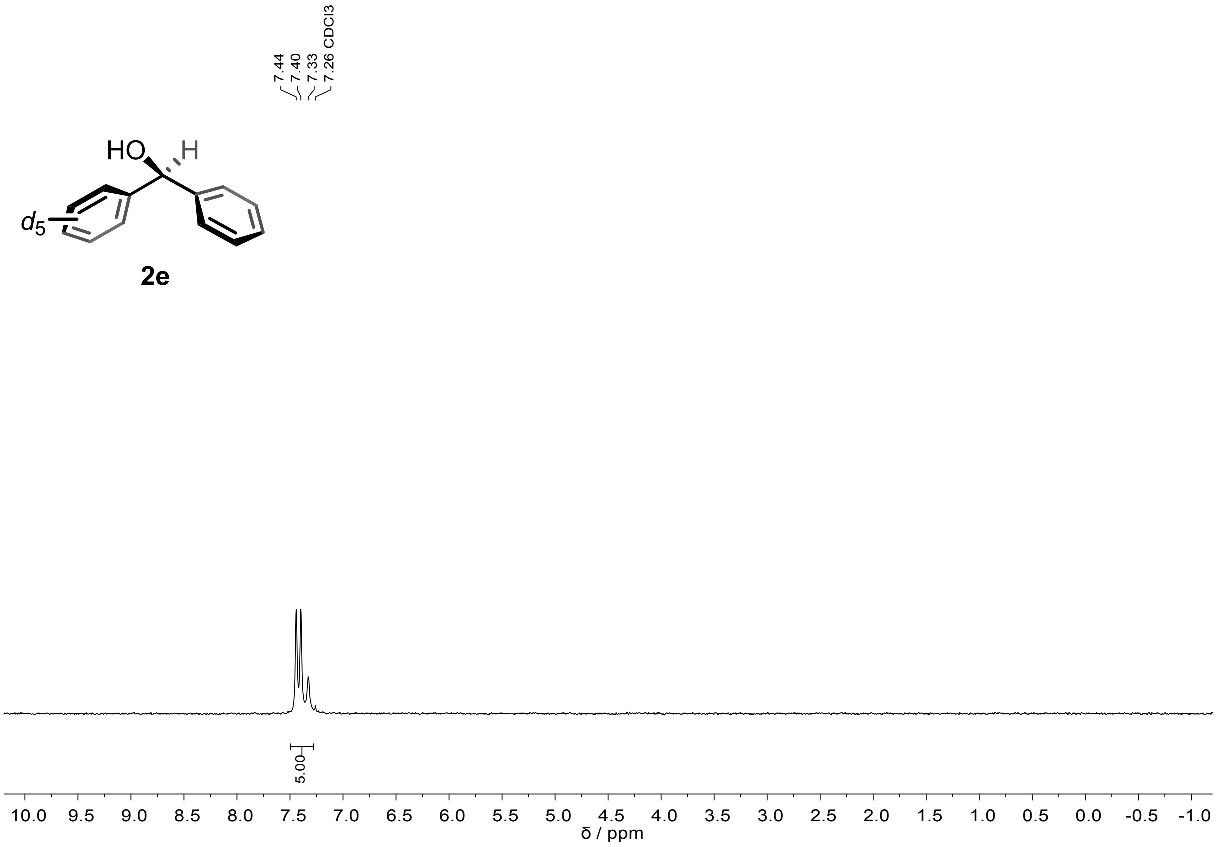

IR (ATR, neat) of **2e**

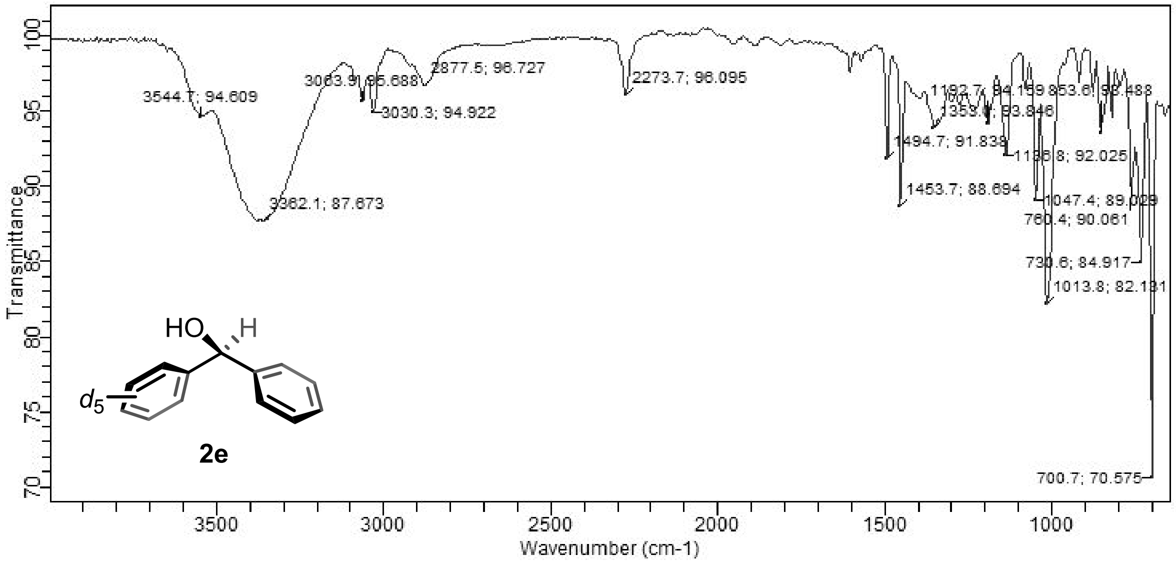

^{1}H NMR (400 MHz, $CDCl_3$) of **3**

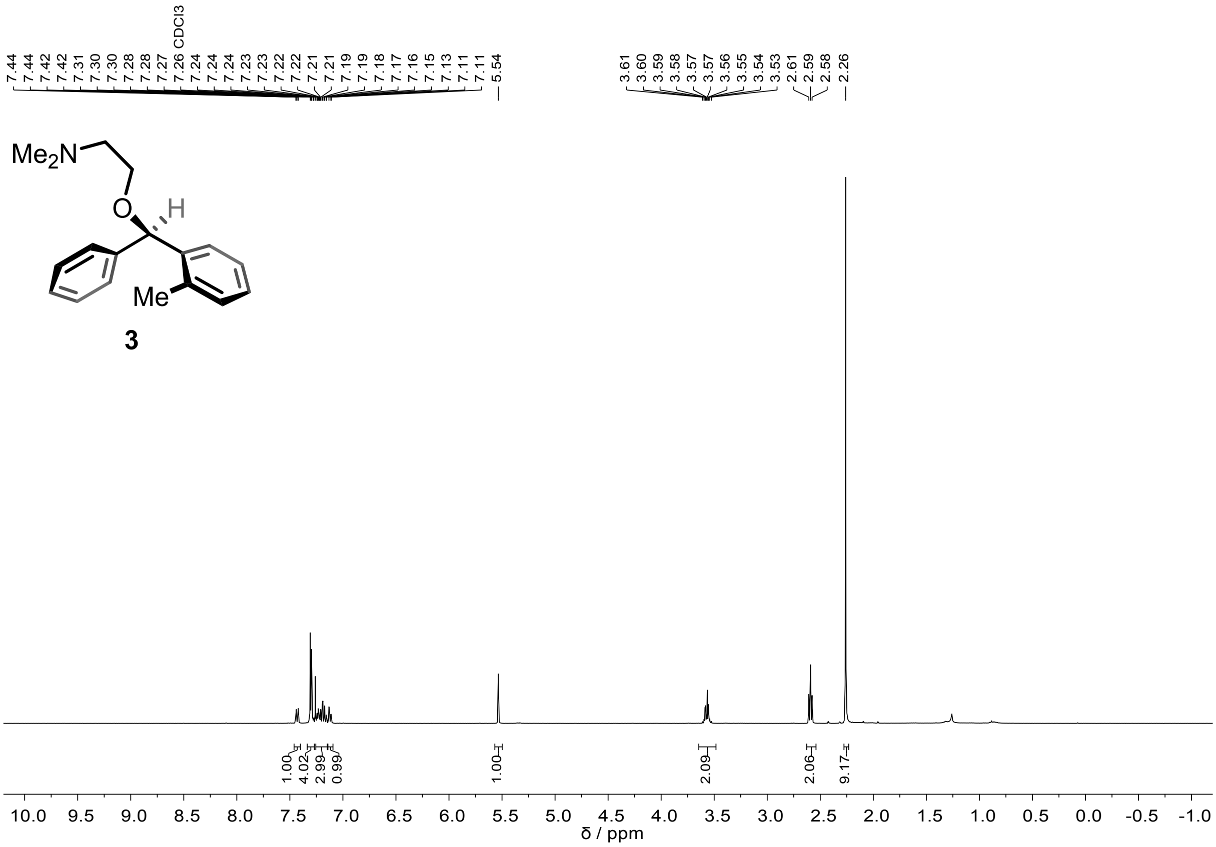

$^{13}C\{^1H\}$ NMR (101 MHz, $CDCl_3$) of **3**

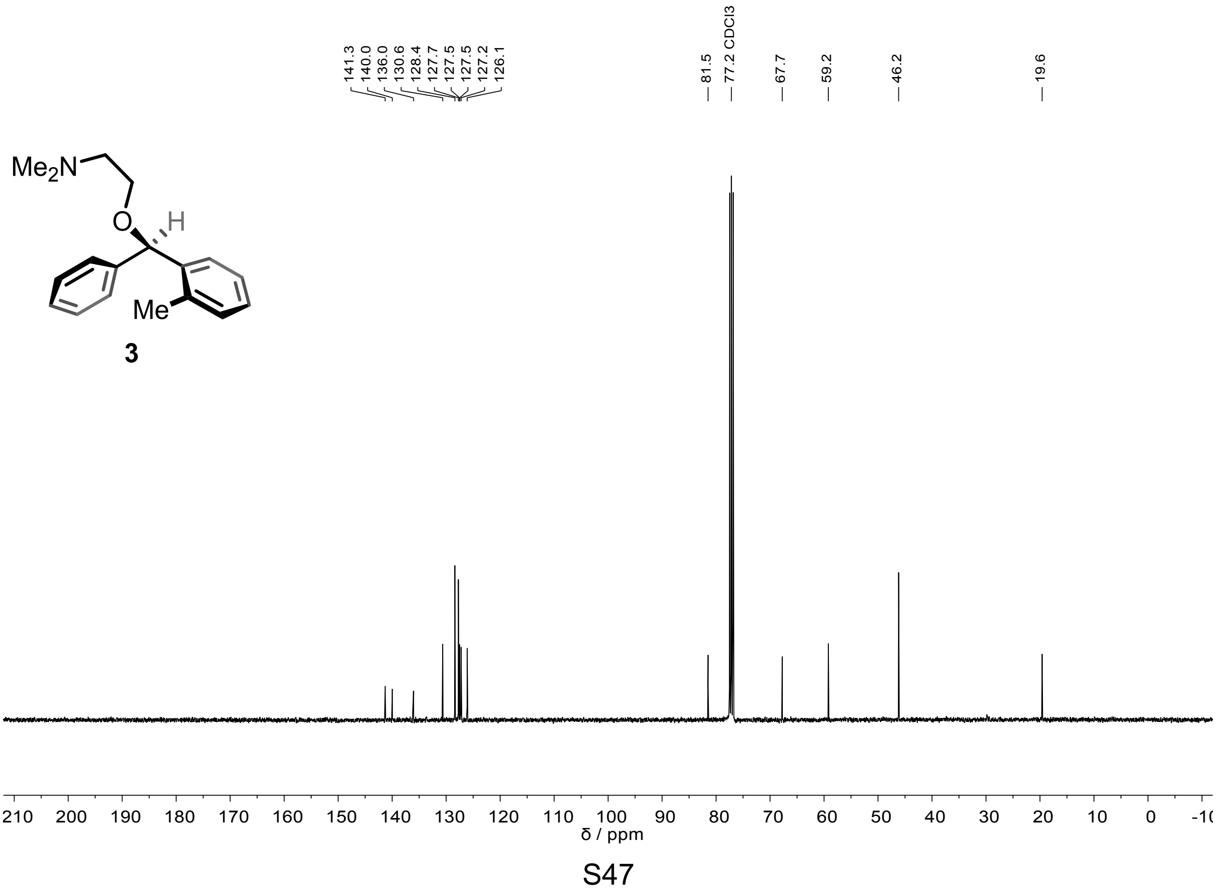

IR (ATR, neat) of **3**

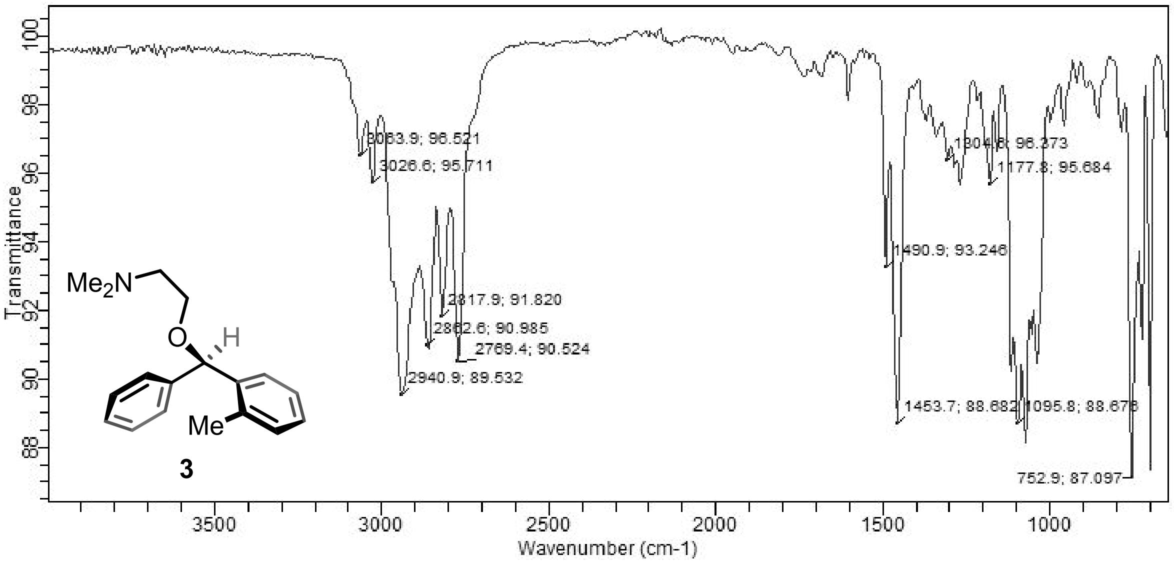

^{1}H NMR (400 MHz, $CDCl_3$) of **3'**

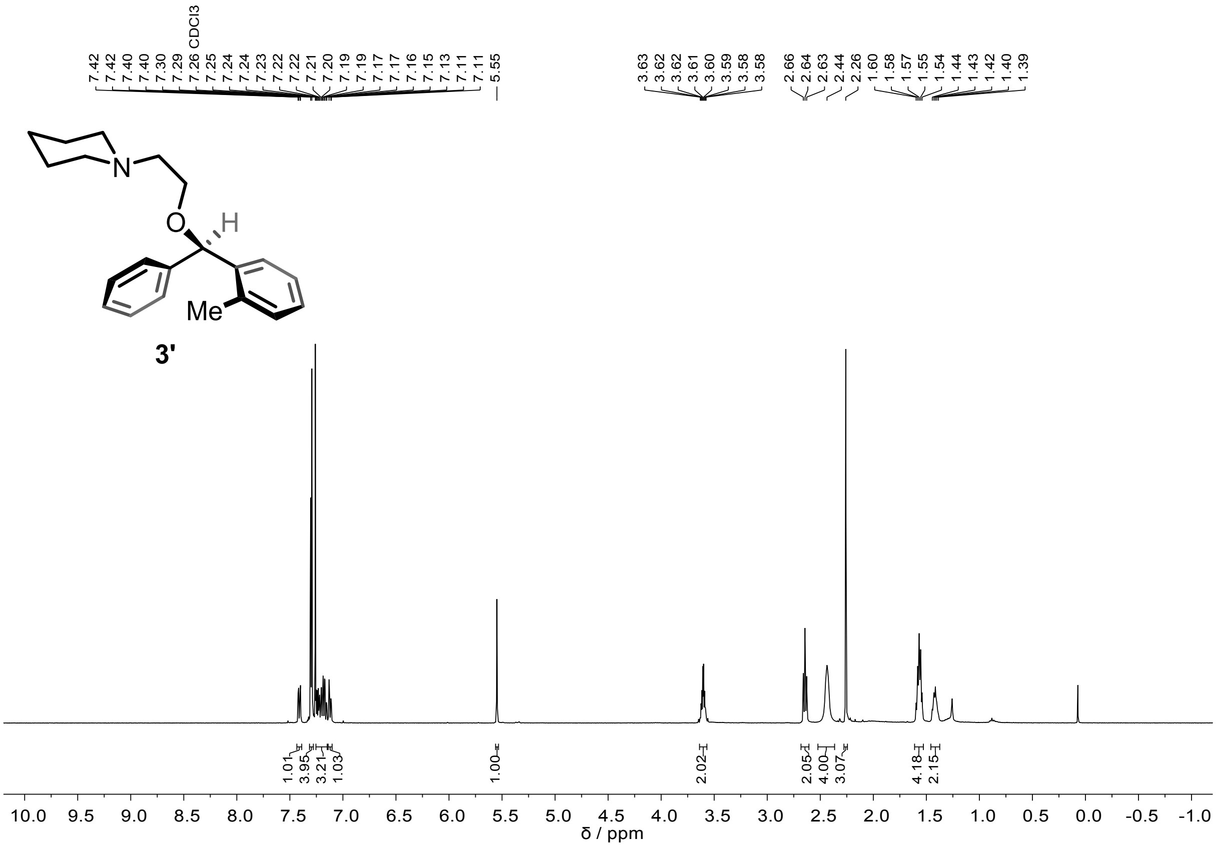

$^{13}C\{^{1}H\}$ NMR (101 MHz, $CDCl_3$) of **3'**

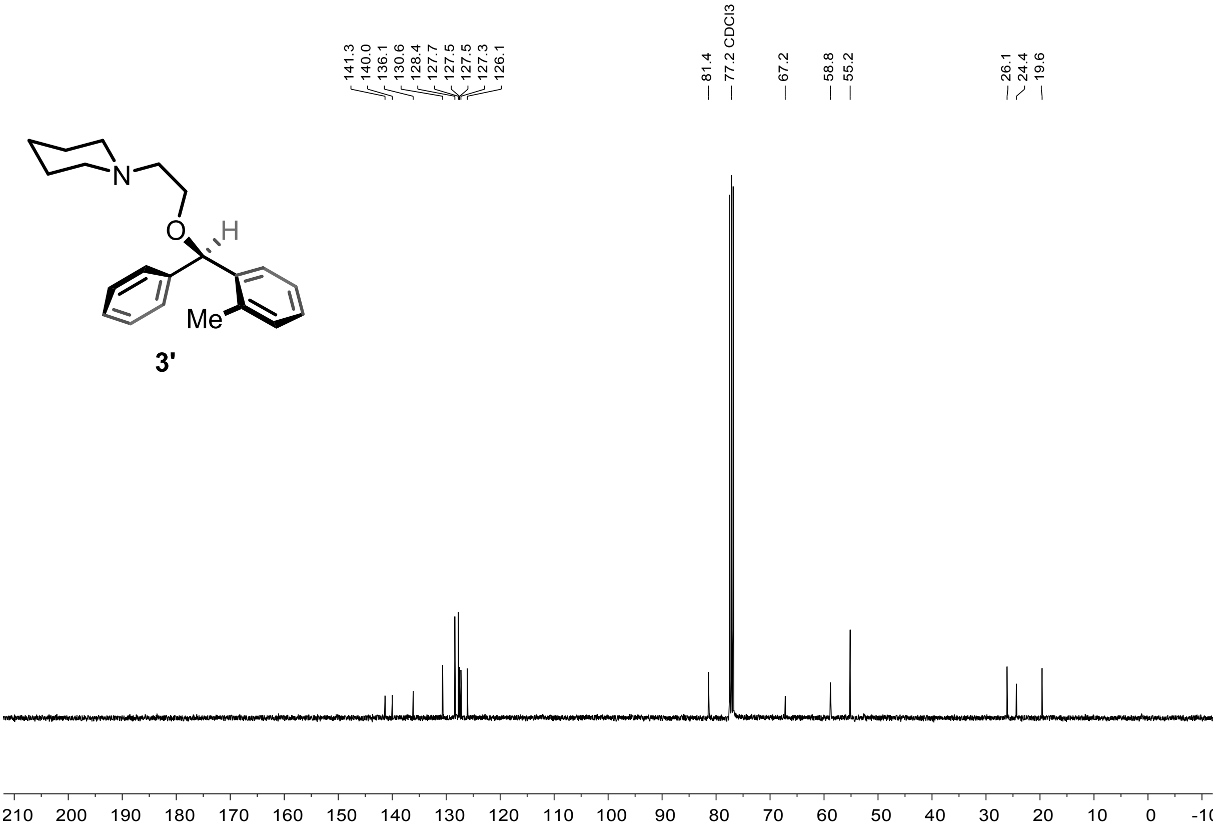

IR (ATR, neat) of **3’**

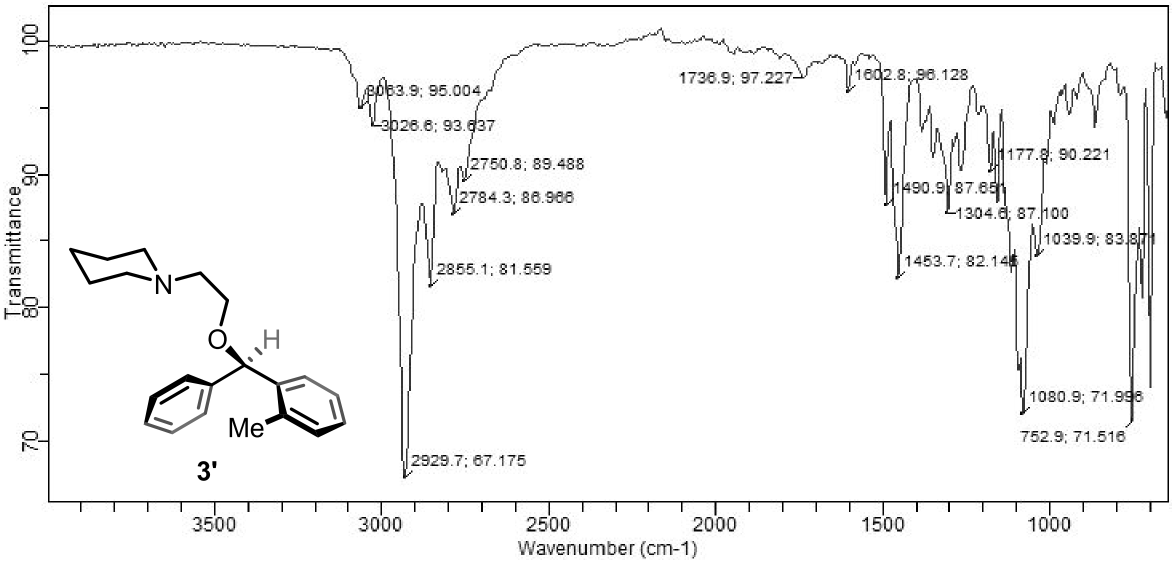

^{1}H NMR (300 MHz, $CDCl_3$) of **4**

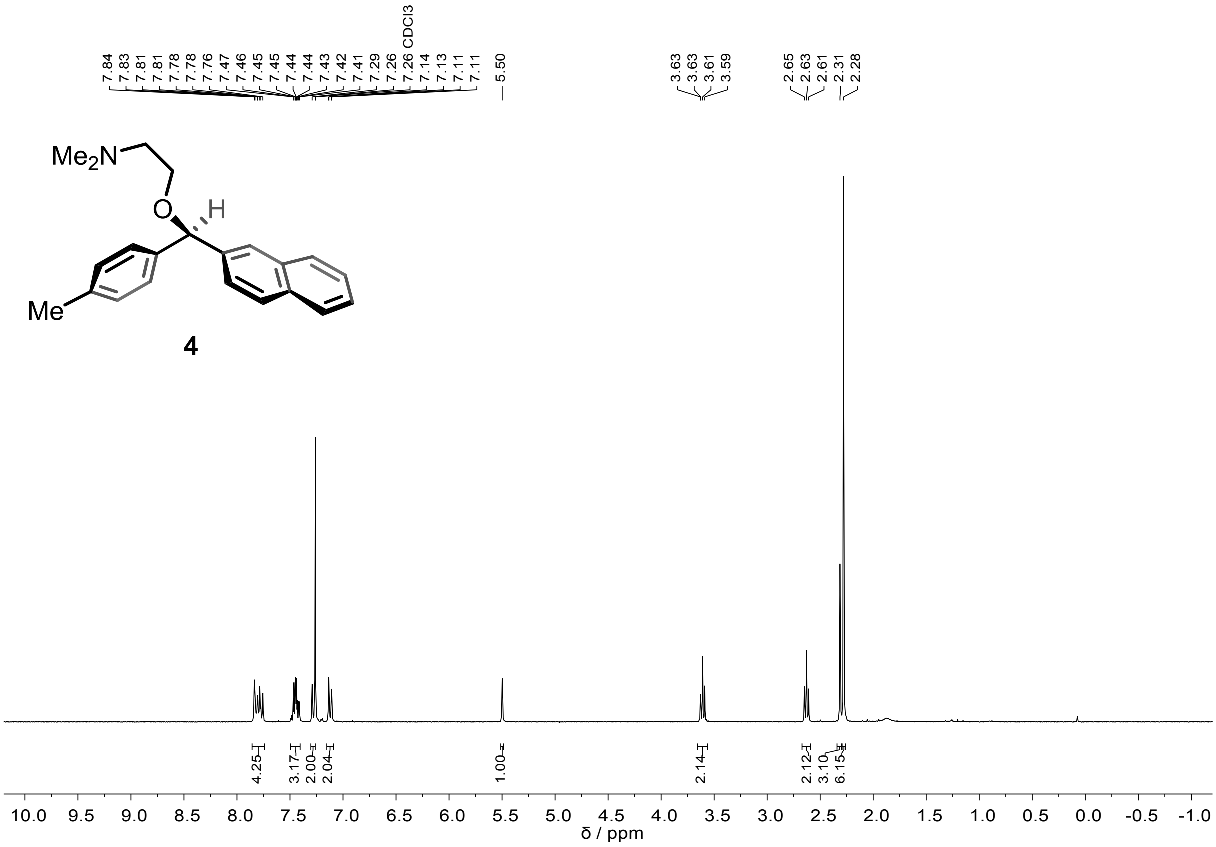

^{13}C{^{1}H} NMR (101 MHz, $CDCl_3$) of **4**

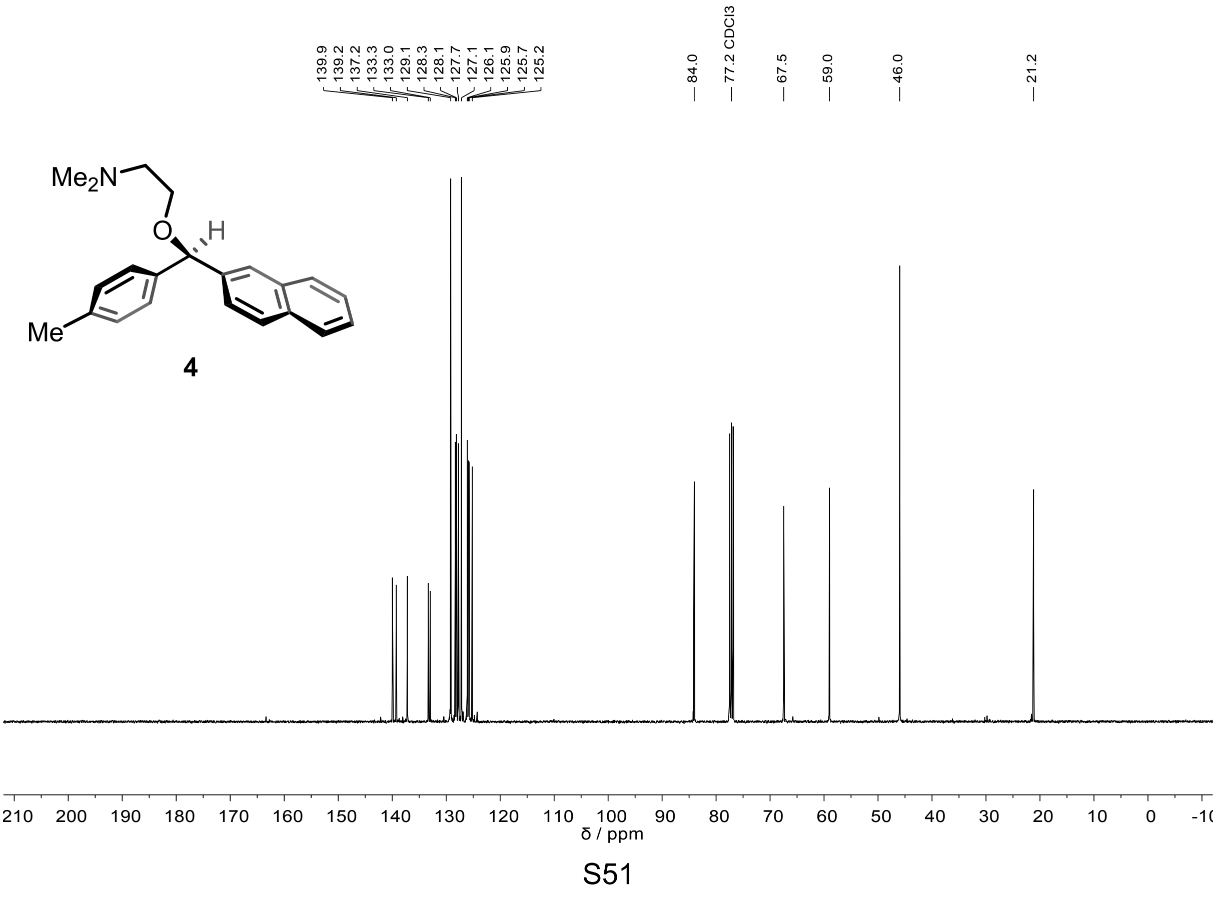

IR (ATR, neat) of **4**

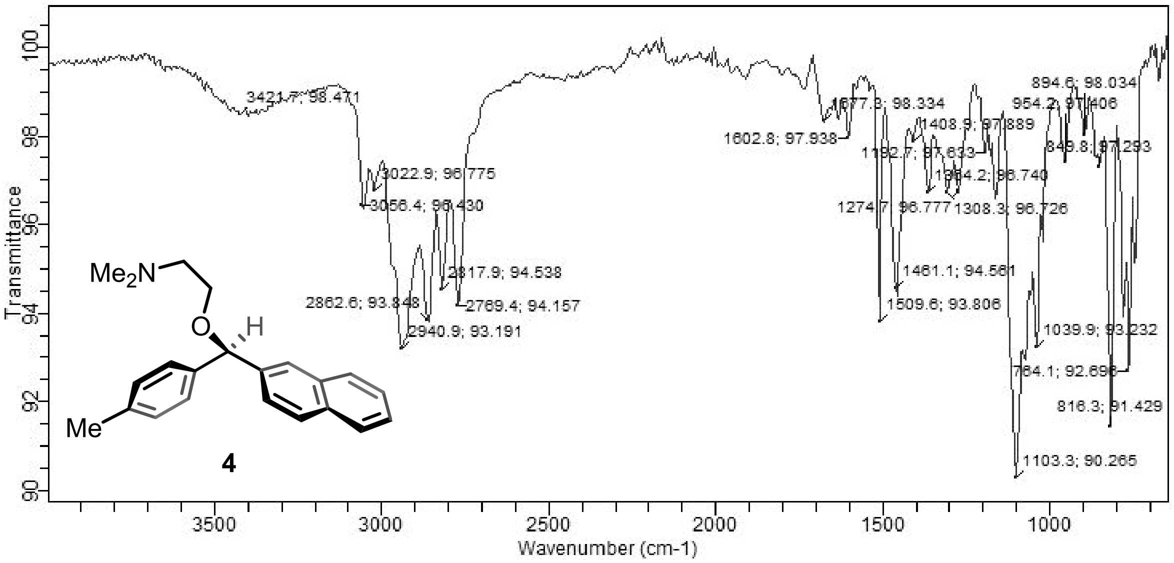

^{1}H NMR (300 MHz, $CDCl_3$) of **6**

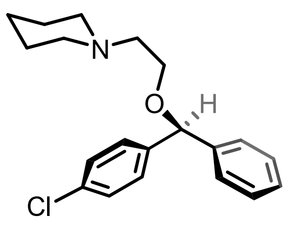

6

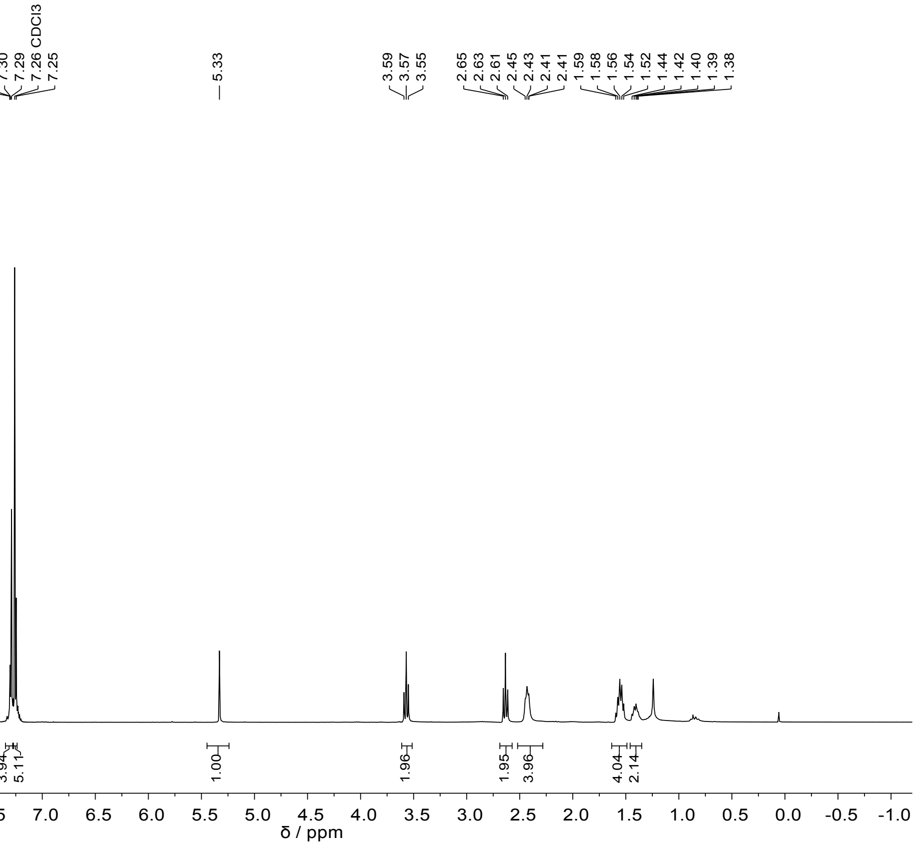

^{13}C{^{1}H} NMR (75 MHz, $CDCl_3$) of **6**

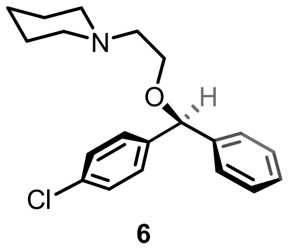

6

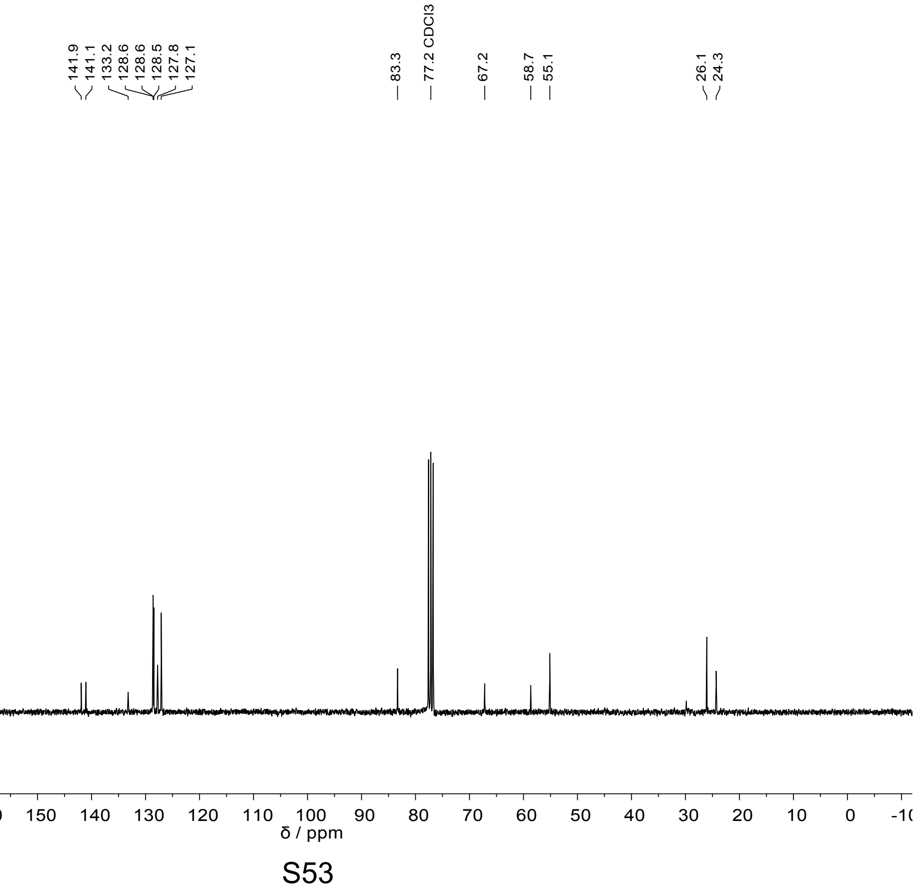

IR (ATR, neat) of **6**

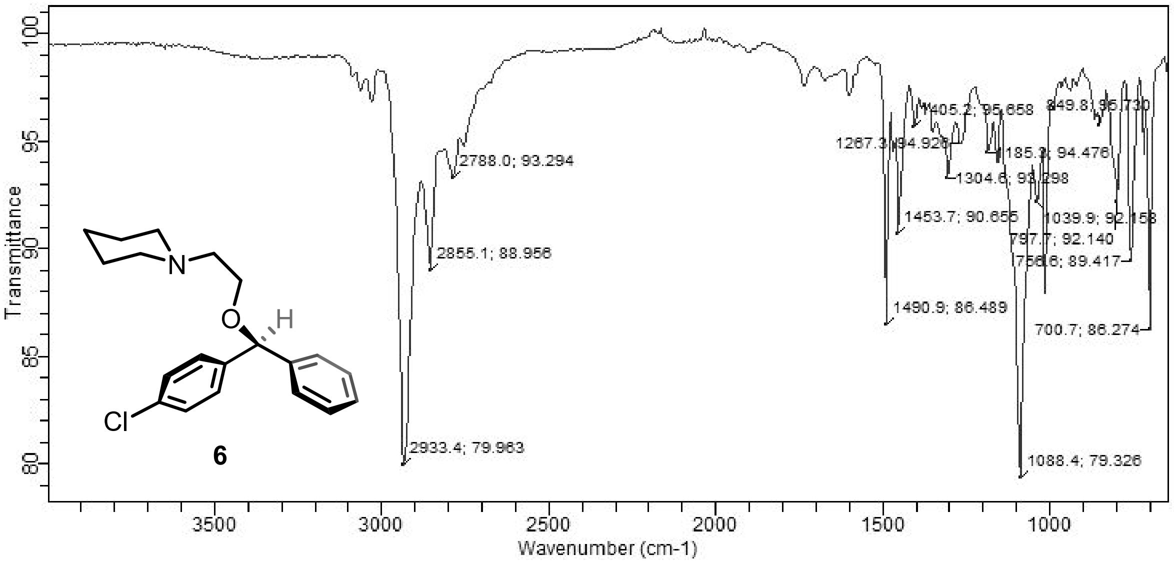

^{1}H NMR (400 MHz, $CDCl_3$) of **7**

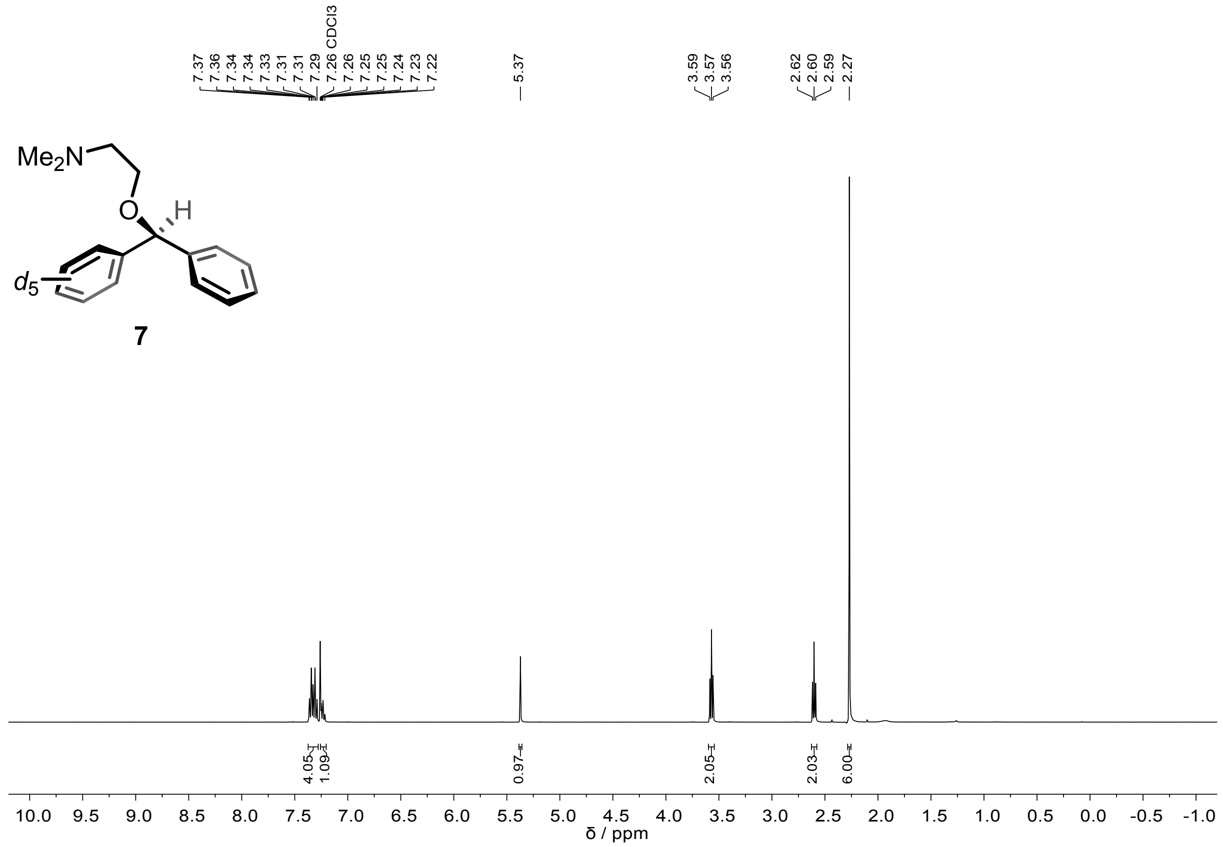

^{13}C{^{1}H} NMR (126 MHz, $CDCl_3$) of **7**

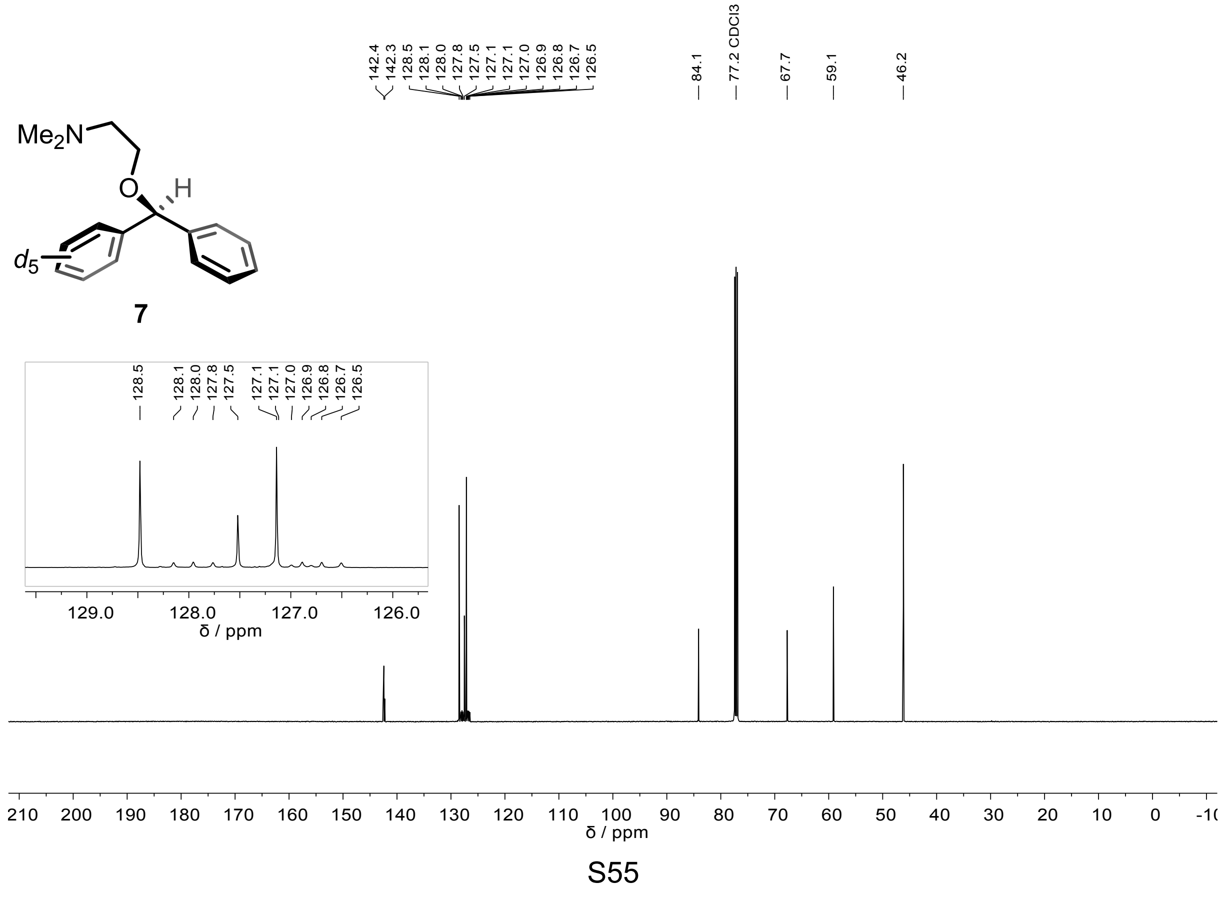

^{2}H NMR (61 MHz, $CHCl_3$) of **7**

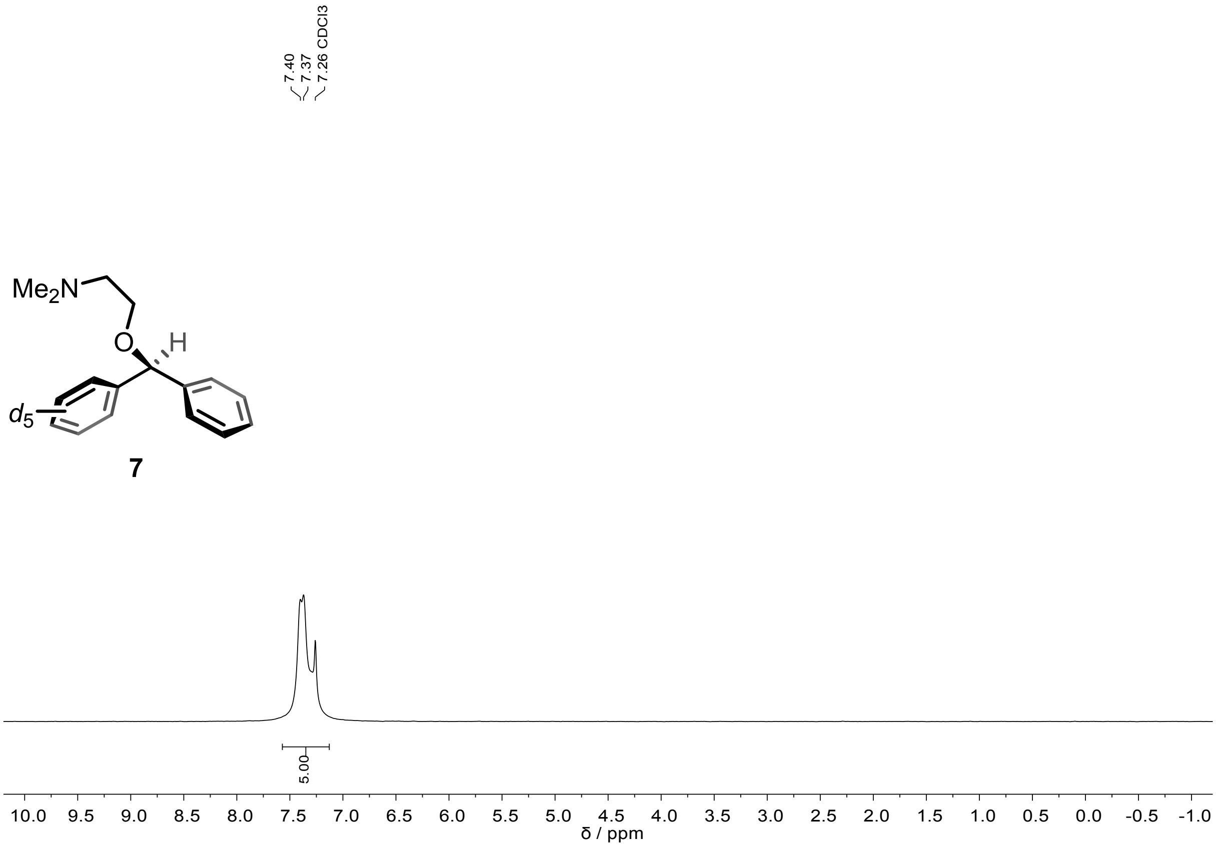

IR (ATR, neat) of **7**

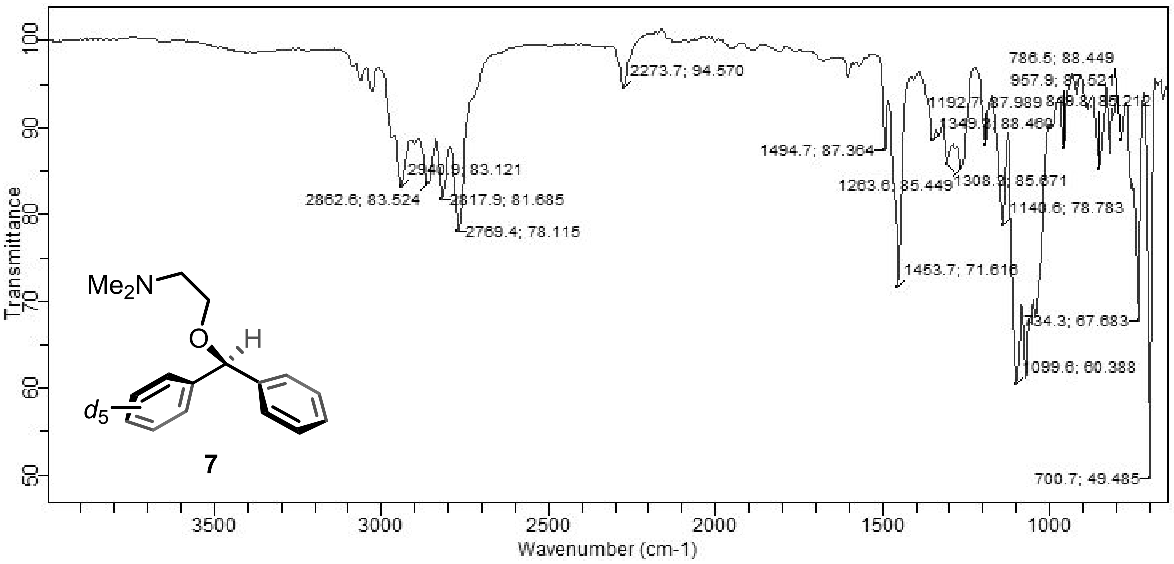

^{1}H NMR (400 MHz, $CDCl_3$) of crude **S9**

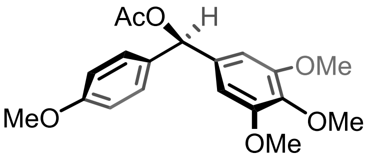

S9

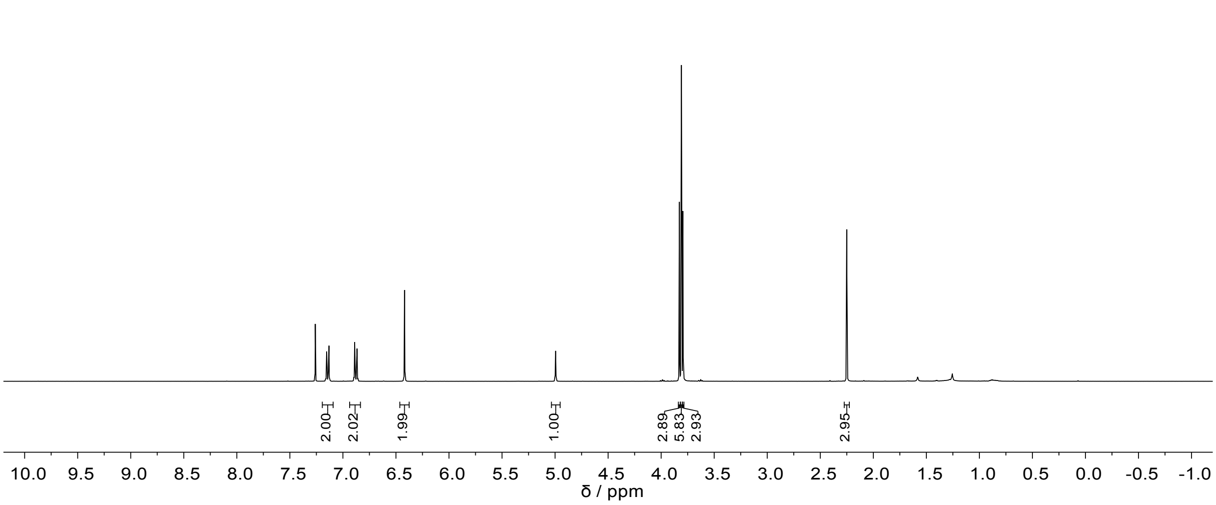

^{1}H NMR (300 MHz, $CDCl_3$) of crude **S10**

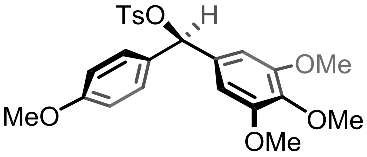

S10

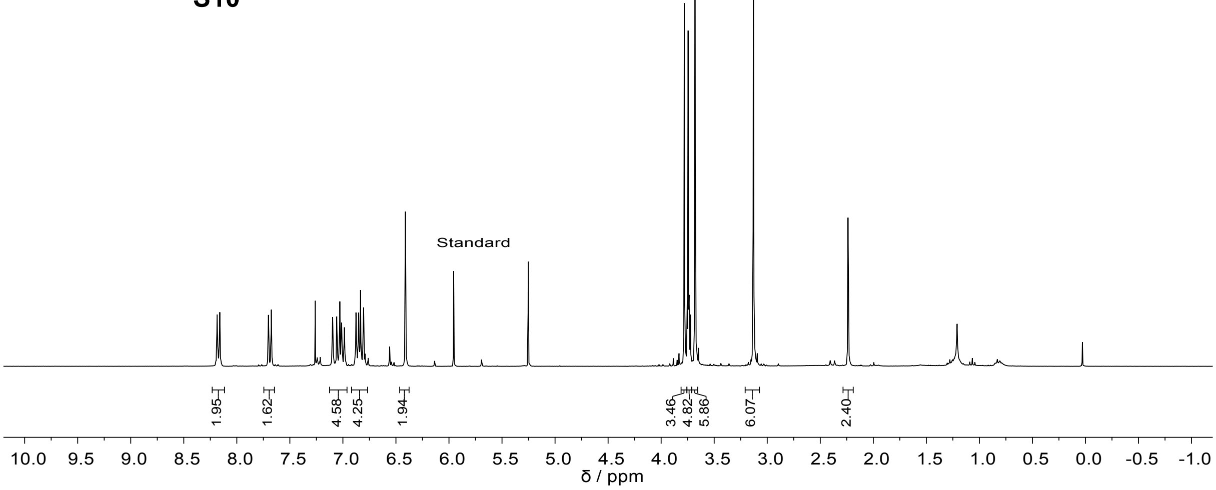

^{1}H NMR (400 MHz, $CDCl_3$) of **S11**

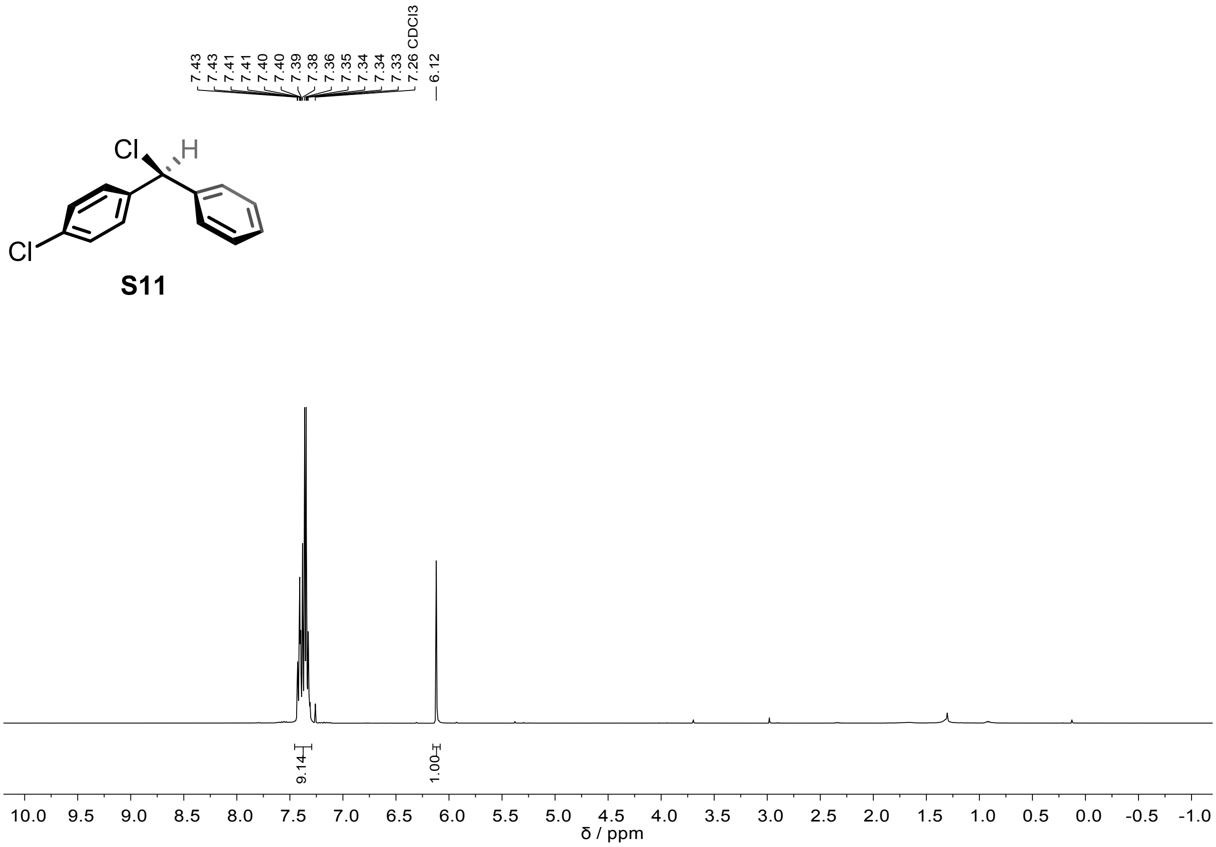

$^{13}C\{^{1}H\}$ NMR (101 MHz, $CDCl_3$) of **S11**

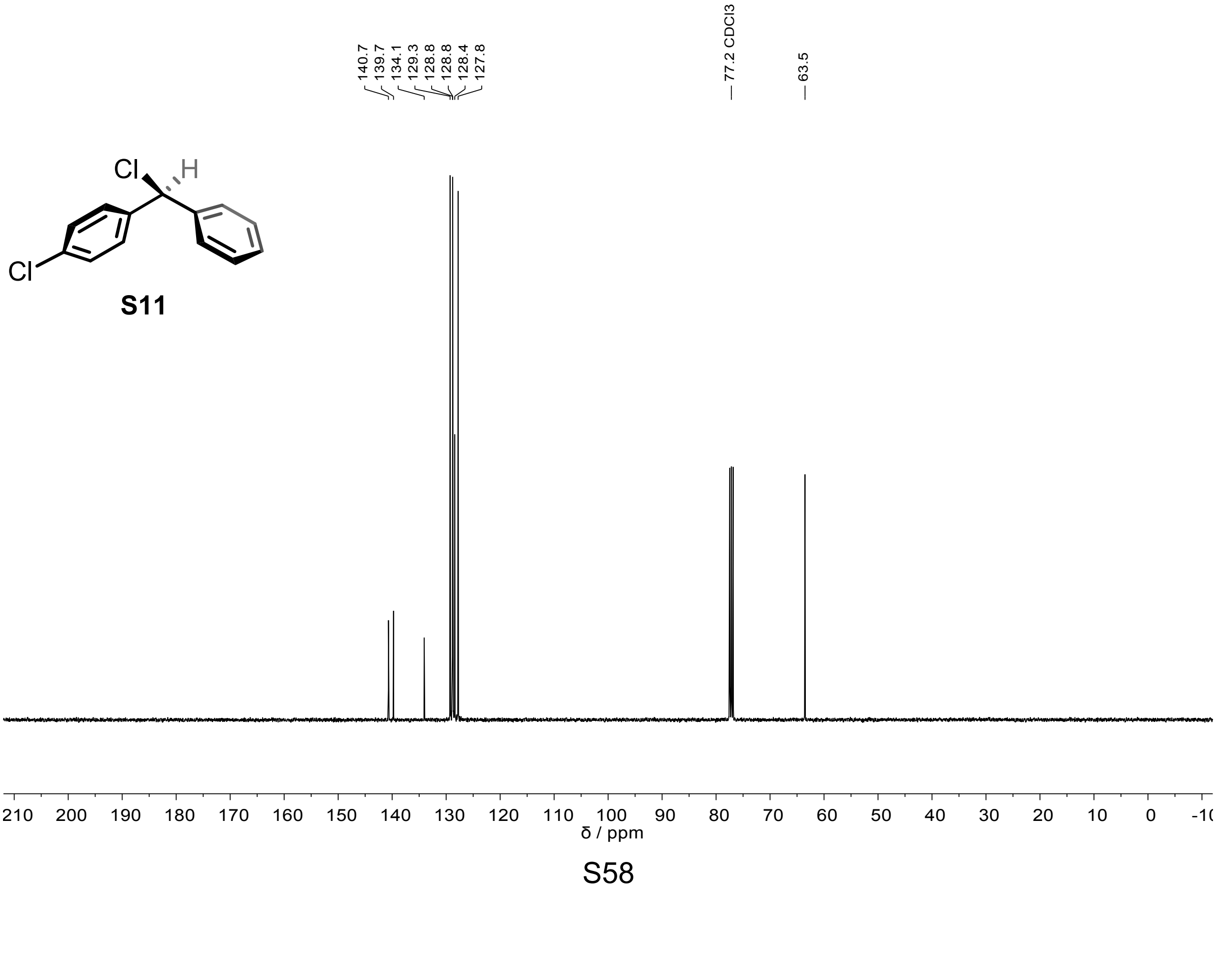

IR (ATR, neat) of **S11**

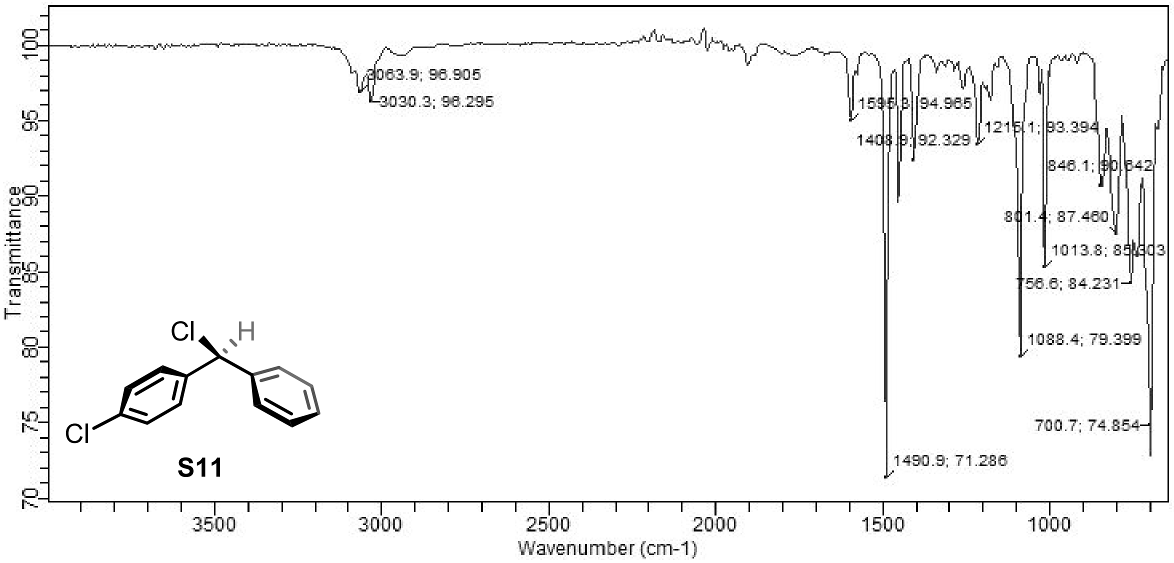

^{1}H NMR (400 MHz, $CDCl_3$) of **8**

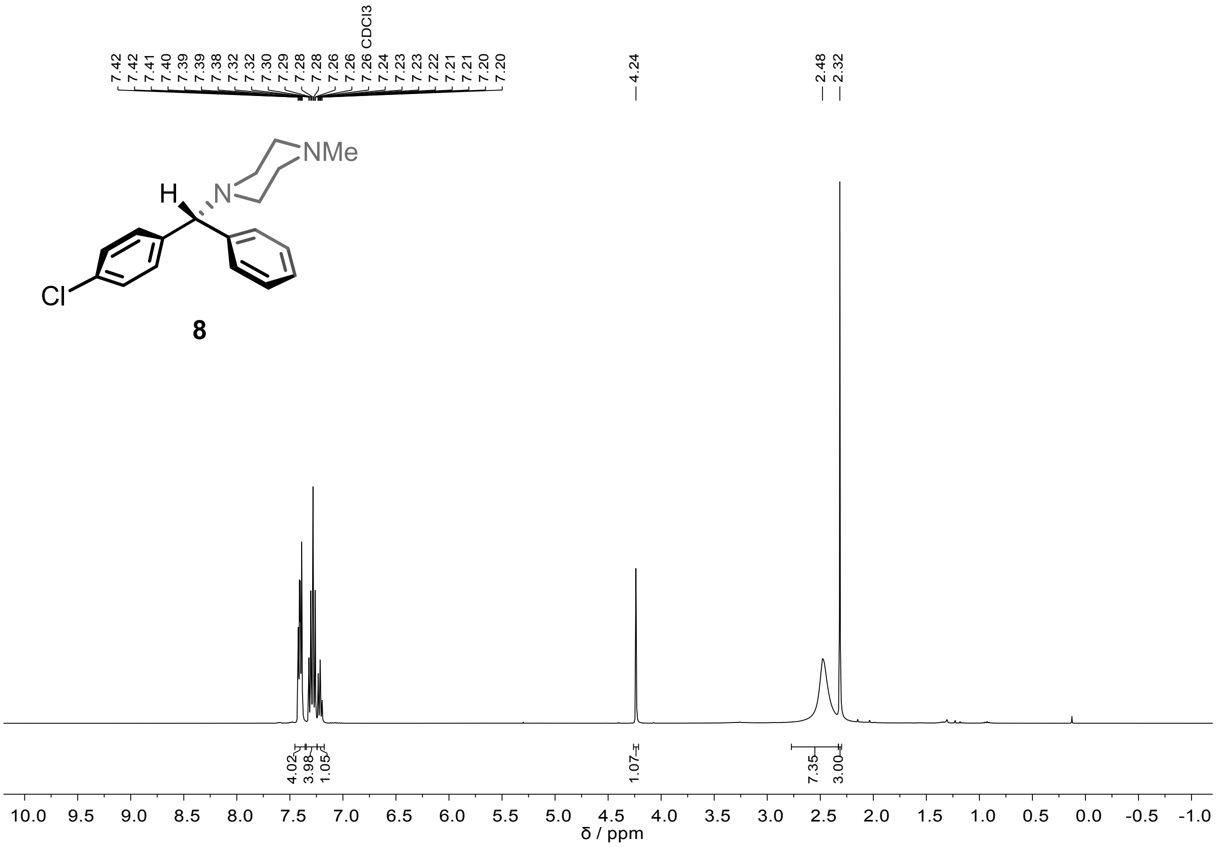

$^{13}C\{^{1}H\}$ NMR (101 MHz, $CDCl_3$) of **8**

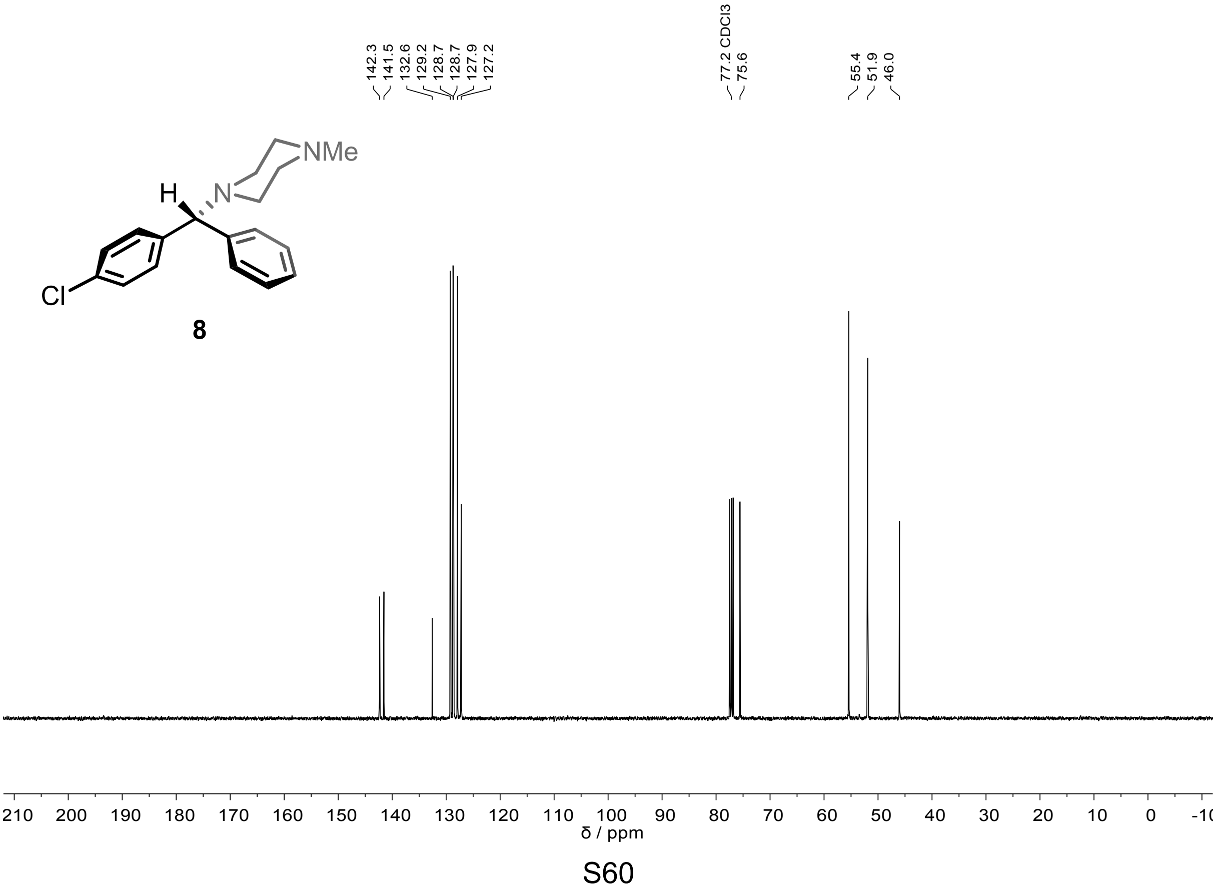

IR (ATR, neat) of **8**

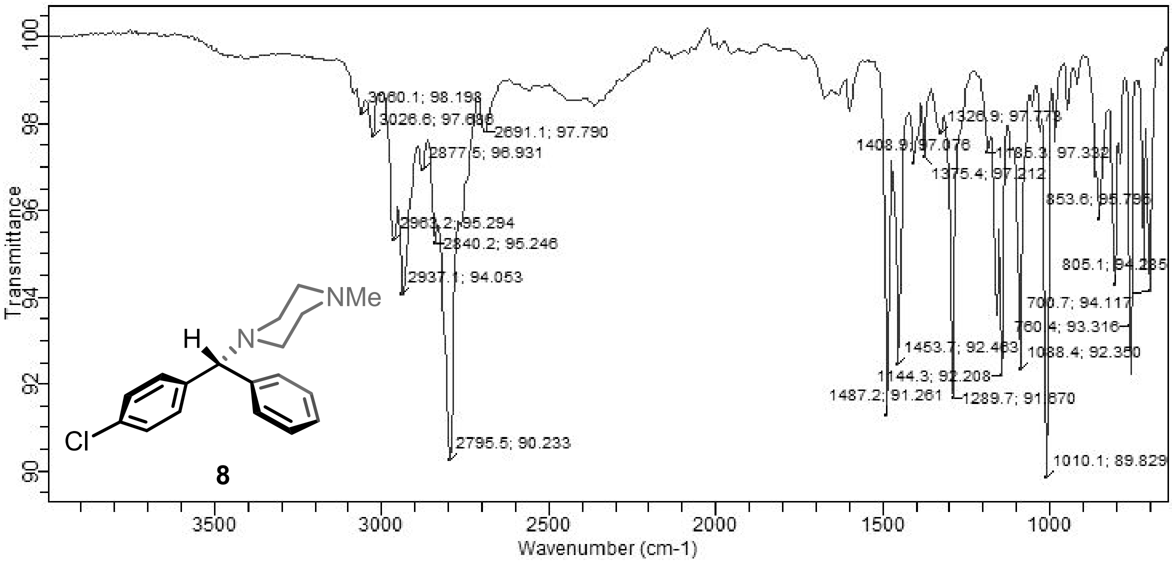

9. HPLC data

(*R*)-1-Phenyl-1-(*o*-tolyl)propan-2-one (1a)

IC-3, *n*-hexane:*i*-PrOH 99:1, flow rate 0.6 mL/min, 220 nm, 25 °C

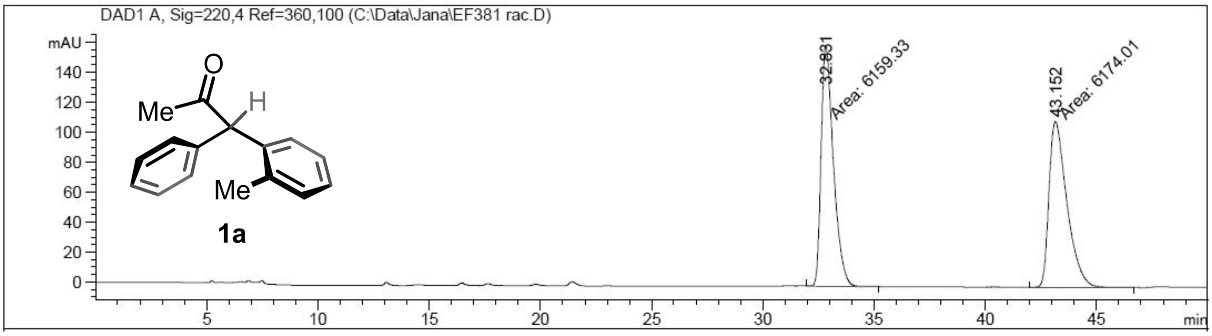

Signal 1: DAD1 A, Sig=220,4 Ref=360,100

Peak #	RetTime [min]	Type	Width [min]	Area [mAU*s]	Height [mAU]	Area %
1	32.831	MM	0.6406	6159.32910	160.25870	49.9405
2	43.152	MM	0.9318	6174.00879	110.43122	50.0595

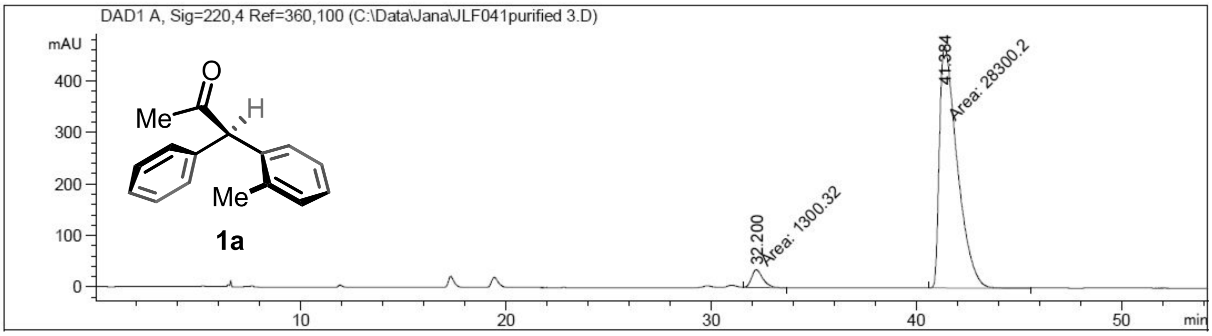

Signal 1: DAD1 A, Sig=220,4 Ref=360,100

Peak #	RetTime [min]	Type	Width [min]	Area [mAU*s]	Height [mAU]	Area %
1	32.200	MM	0.6309	1300.31995	34.35051	4.3929
2	41.384	MM	1.0036	2.83002e4	469.98840	95.6071

(*R*)-1-(Naphthalen-2-yl)-1-(*p*-tolyl)propan-2-one (1b)

ID-3, *n*-hexane:*i*-PrOH 98:2, flow rate 0.8 mL/min, 220 nm, 25 °C

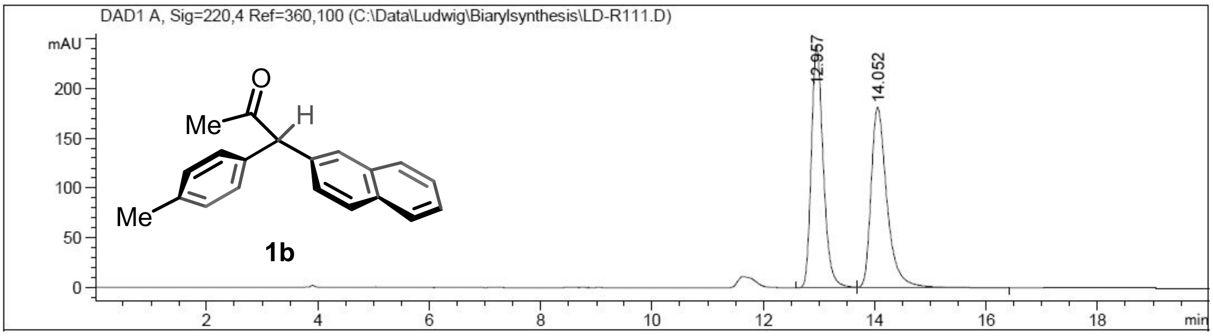

Signal 1: DAD1 A, Sig=220,4 Ref=360,100

Peak #	RetTime [min]	Type	Width [min]	Area [mAU*s]	Height [mAU]	Area %
1	12.957	BV	0.2300	3622.04395	242.82278	49.9335
2	14.052	VB	0.3033	3631.68994	181.63216	50.0665

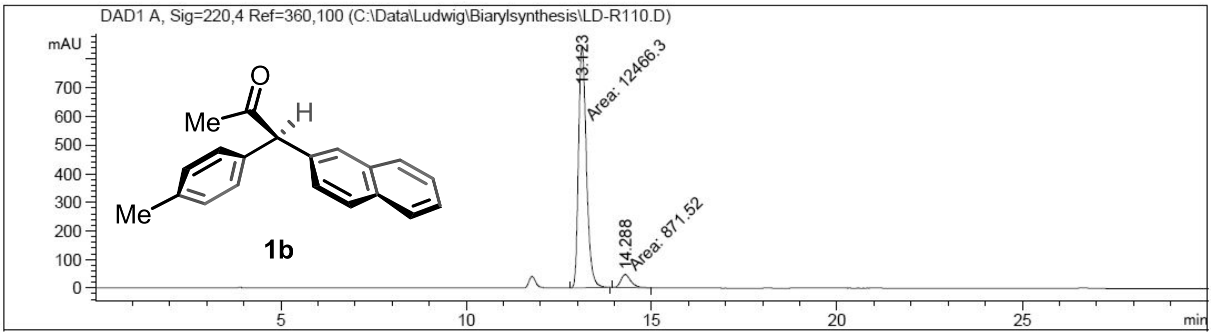

Signal 1: DAD1 A, Sig=220,4 Ref=360,100

Peak #	RetTime [min]	Type	Width [min]	Area [mAU*s]	Height [mAU]	Area %
1	13.123	MM	0.2463	1.24663e4	843.46277	93.4658
2	14.288	MM	0.3141	871.51996	46.24353	6.5342

(*R*)-1-Phenyl-1-(*p*-tolyl)propan-2-one (1c)

IA-3, *n*-hexane:*i*-PrOH 95:5, flow rate 0.8 mL/min, 220 nm, 25 °C

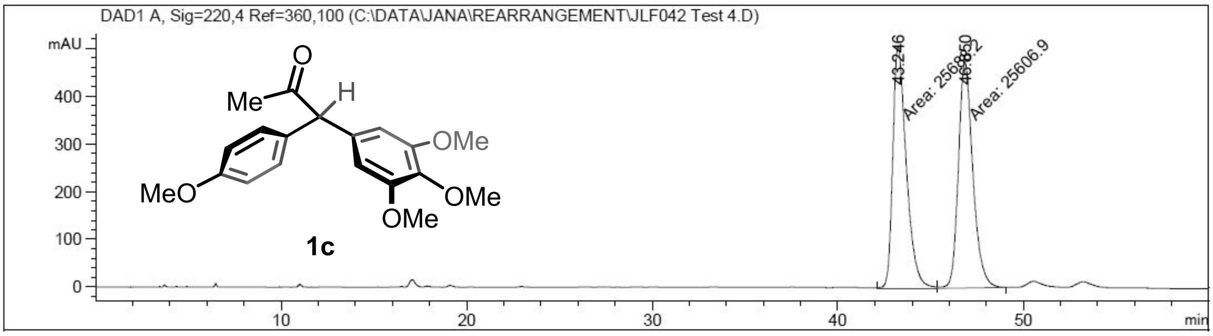


```
Signal 1: DAD1 A, Sig=220,4 Ref=360,100

Peak RetTime Type  Width     Area      Height     Area
  #   [min]        [min]   [mAU*s]     [mAU]        %
----|-------|----|-------|----------|----------|--------|
   1  43.246 MM     0.8454 2.56882e4  506.45117  50.0792
   2  46.850 MM     0.9020 2.56069e4  473.16360  49.9208
```

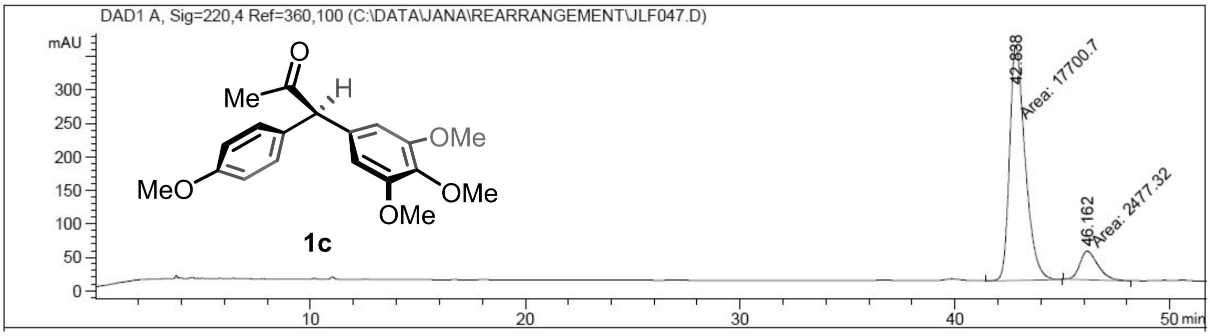


```
Signal 1: DAD1 A, Sig=220,4 Ref=360,100

Peak RetTime Type  Width     Area      Height     Area
  #   [min]        [min]   [mAU*s]     [mAU]        %
----|-------|----|-------|----------|----------|--------|
   1  42.838 MM     0.8416 1.77007e4  350.53458  87.7227
   2  46.162 MM     0.9645 2477.32153   42.80663  12.2773
```

(*S*)-1-(4-Chlorophenyl)-1-phenylpropan-2-one (1d)

ID-3, *n*-hexane:*i*-PrOH 98:2, flow rate 0.8 mL/min, 220 nm, 25 °C

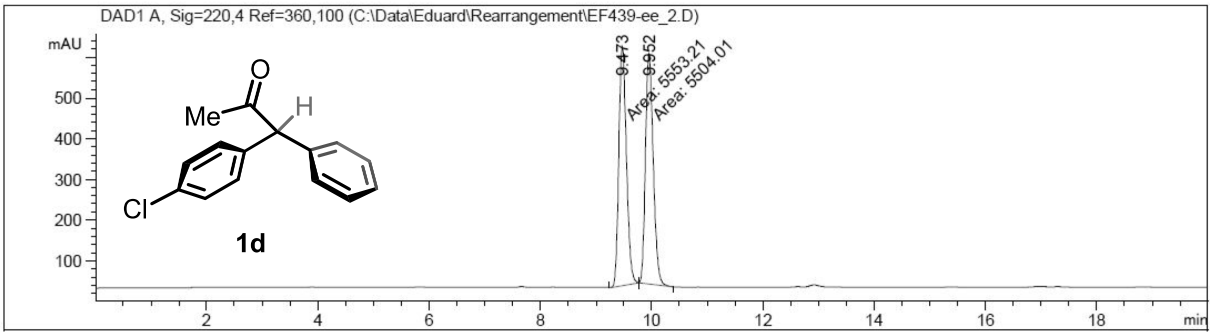

Signal 1: DAD1 A, Sig=220,4 Ref=360,100

Peak #	RetTime [min]	Type	Width [min]	Area [mAU*s]	Height [mAU]	Area %
1	9.473	MM	0.1576	5553.21094	587.12848	50.2225
2	9.952	MM	0.1622	5504.01318	565.57697	49.7775

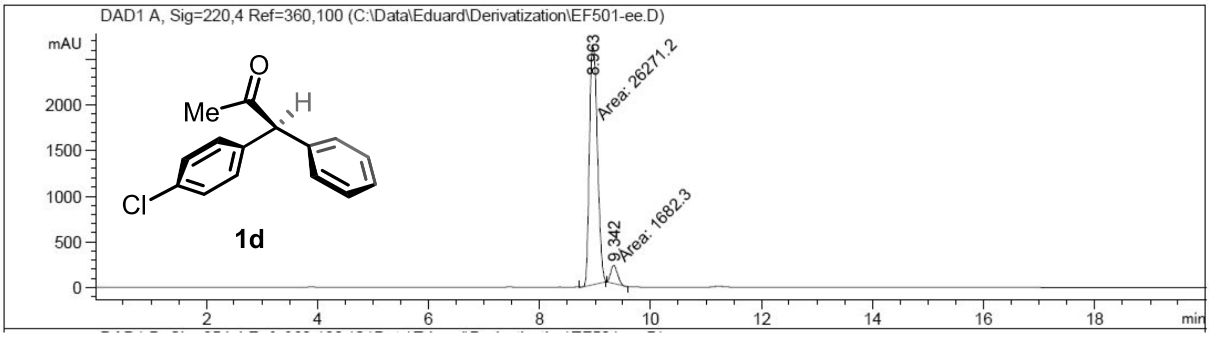

Signal 1: DAD1 A, Sig=220,4 Ref=360,100

Peak #	RetTime [min]	Type	Width [min]	Area [mAU*s]	Height [mAU]	Area %
1	8.963	MM	0.1672	2.62712e4	2618.66748	93.9818
2	9.342	MM	0.1426	1682.30493	196.60786	6.0182

(*R*)-Phenyl(*o*-tolyl)methanol (2a)

IC-3, *n*-hexane:*i*-PrOH 95:5, flow rate 0.8 mL/min, 220 nm, 25 °C

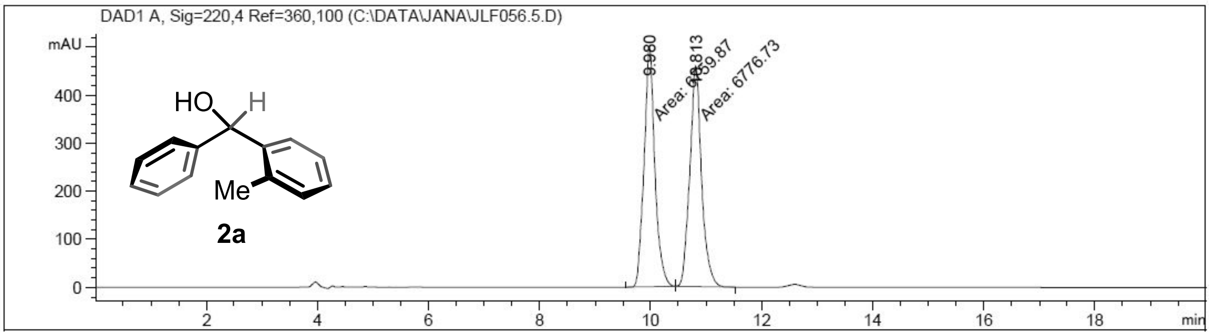

Signal 1: DAD1 A, Sig=220,4 Ref=360,100

Peak #	RetTime [min]	Type	Width [min]	Area [mAU*s]	Height [mAU]	Area %
1	9.980	MM	0.2254	6759.87402	499.90536	49.9377
2	10.813	MM	0.2458	6776.73291	459.56058	50.0623

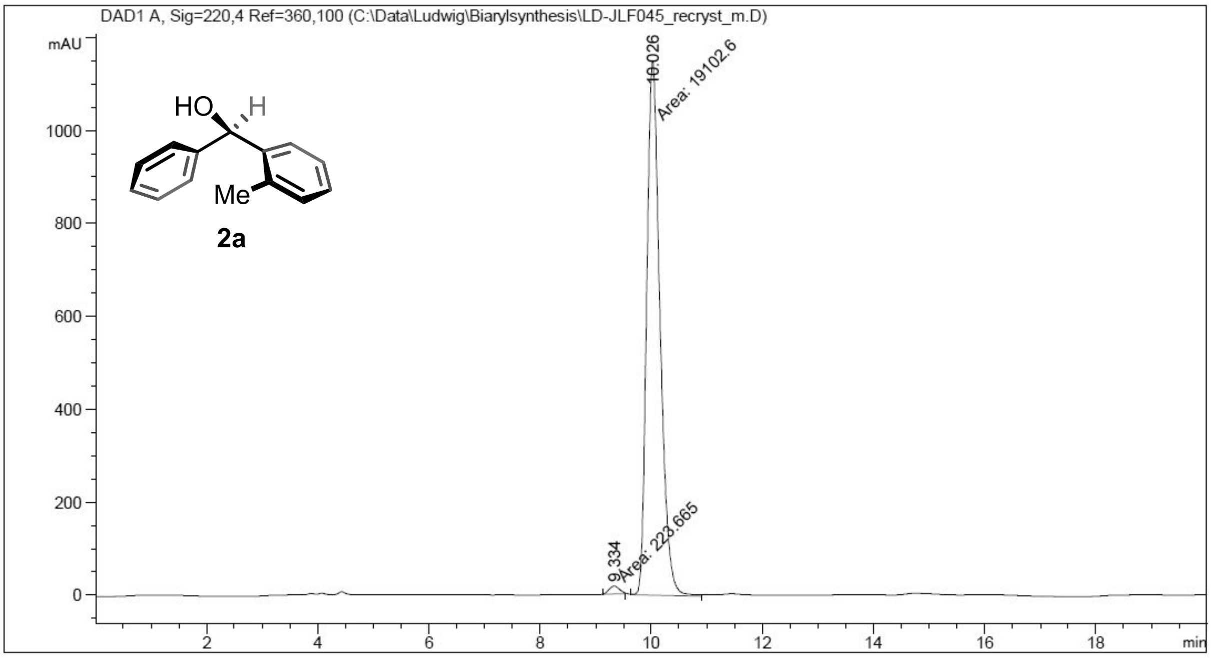

Signal 1: DAD1 A, Sig=220,4 Ref=360,100

Peak #	RetTime [min]	Type	Width [min]	Area [mAU*s]	Height [mAU]	Area %
1	9.334	MM	0.2171	223.66527	17.17059	1.1573
2	10.026	MM	0.2772	1.91026e4	1148.48877	98.8427

(*R*)-Naphthalen-2-yl(*p*-tolyl)methanol (2b)

OD-3, *n*-hexane:*i*-PrOH 95:5, flow rate 0.8 mL/min, 250 nm, 25 °C

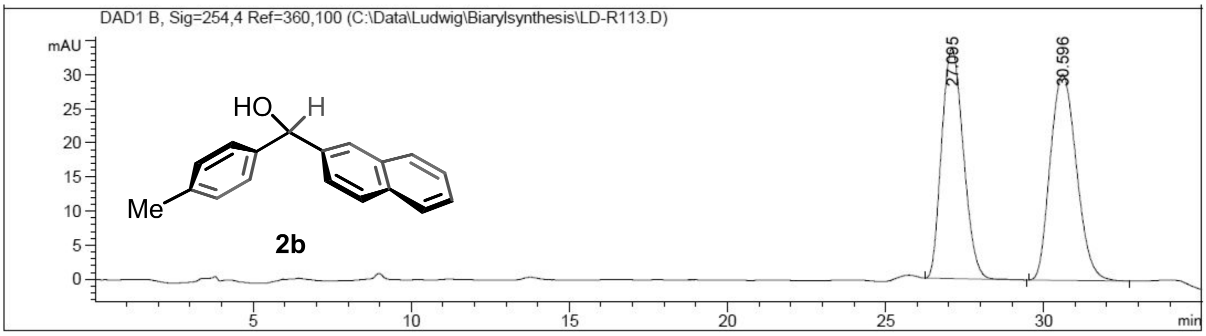

Signal 2: DAD1 B, Sig=254,4 Ref=360,100

Peak #	RetTime [min]	Type	Width [min]	Area [mAU*s]	Height [mAU]	Area %
1	27.095	BB	0.7627	1612.86279	33.67892	48.1840
2	30.596	BB	0.9166	1734.43872	30.17459	51.8160

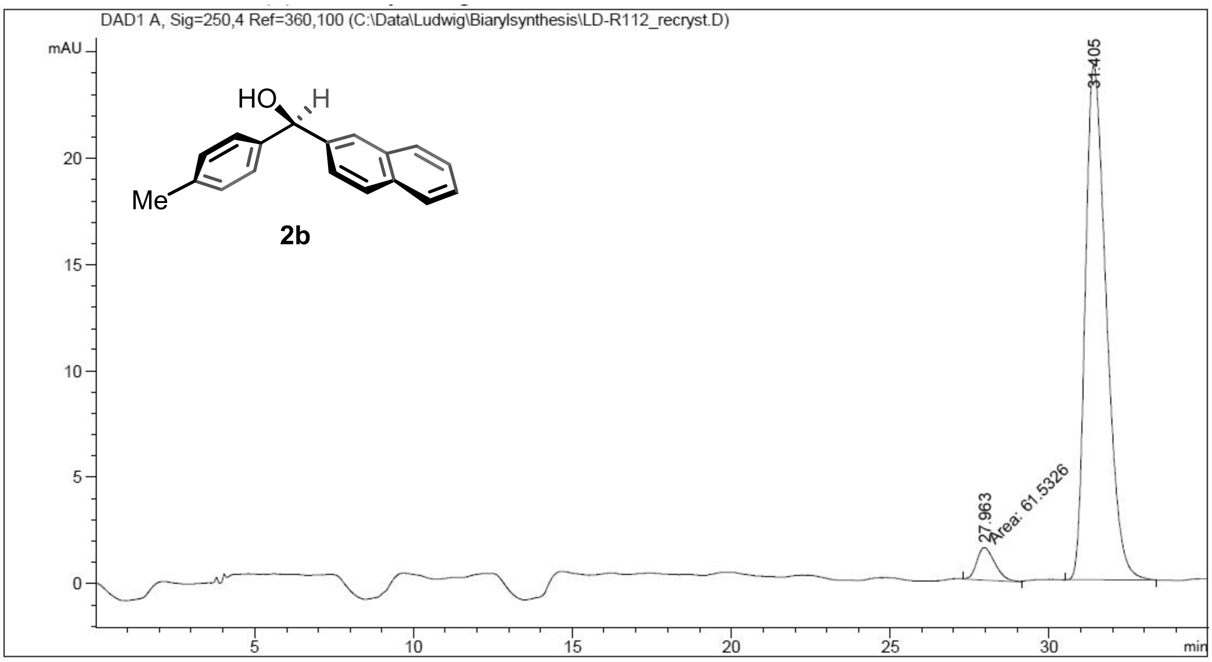

Signal 1: DAD1 A, Sig=250,4 Ref=360,100

Peak #	RetTime [min]	Type	Width [min]	Area [mAU*s]	Height [mAU]	Area %
1	27.963	MM	0.6682	61.53258	1.53476	5.2421
2	31.405	BB	0.6902	1112.27991	24.20239	94.7579

(*R*)-(4-Methoxyphenyl)(3,4,5-trimethoxyphenyl)methanol (2c)

IB-3, *n*-hexane:*i*-PrOH 90:10 to 80:20, flow rate 0.8 mL/min, 220 nm, 25 °C

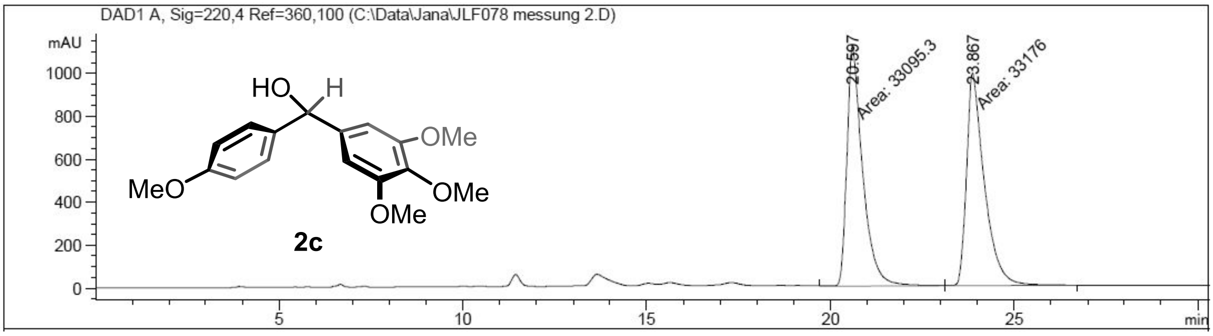


```
Signal 1: DAD1 A, Sig=220,4 Ref=360,100

Peak RetTime Type  Width     Area      Height     Area
  #   [min]        [min]   [mAU*s]     [mAU]        %
----|-------|----|-------|----------|----------|--------|
   1  20.597 MM    0.4933 3.30953e4 1118.13037  49.9391
   2  23.867 MM    0.5590 3.31760e4  989.08502  50.0609
```

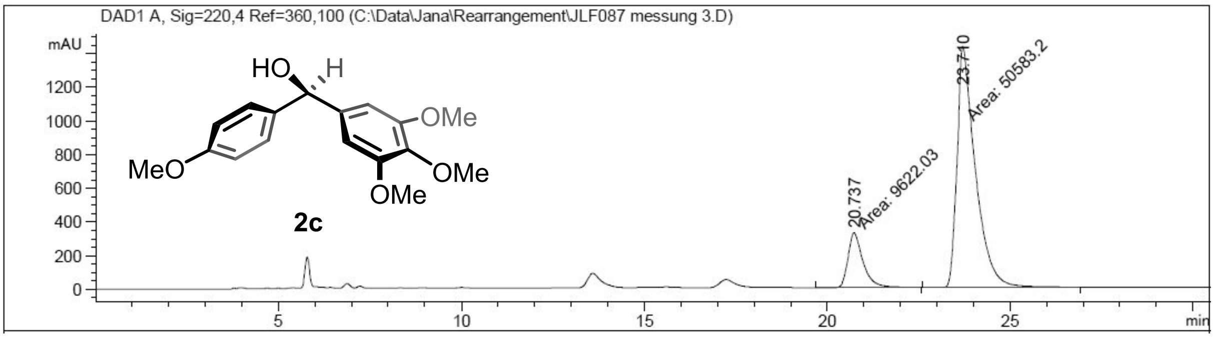


```
Signal 1: DAD1 A, Sig=220,4 Ref=360,100

Peak RetTime Type  Width     Area      Height     Area
  #   [min]        [min]   [mAU*s]     [mAU]        %
----|-------|----|-------|----------|----------|--------|
   1  20.737 MM    0.4898 9622.03223  327.39017  15.9821
   2  23.710 MM    0.5877 5.05832e4 1434.37634  84.0179
```

(*S*)-(4-Chlorophenyl)(phenyl)methanol (2d)

IA-3, *n*-hexane:*i*-PrOH 98:2, flow rate 0.8 mL/min, 220 nm, 25 °C

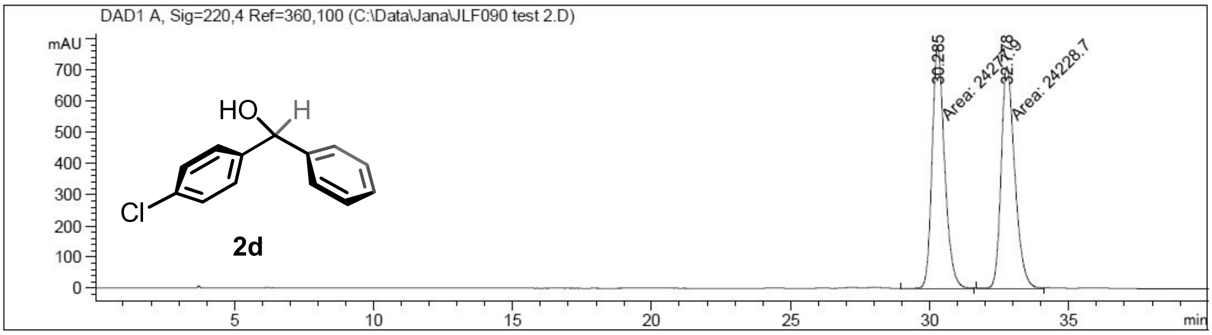


```
Signal 1: DAD1 A, Sig=220,4 Ref=360,100

Peak RetTime Type  Width     Area      Height     Area
  #   [min]        [min]   [mAU*s]     [mAU]        %
----|-------|----|-------|----------|----------|--------|
   1  30.285 MM    0.5214 2.42779e4  776.00793  50.0507
   2  32.778 MM    0.5676 2.42287e4  711.39172  49.9493
```

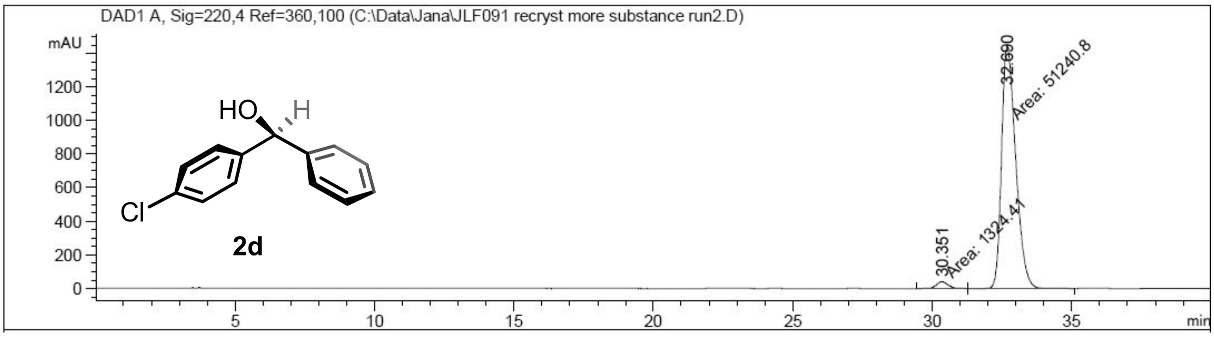


```
Signal 1: DAD1 A, Sig=220,4 Ref=360,100

Peak RetTime Type  Width     Area      Height     Area
  #   [min]        [min]   [mAU*s]     [mAU]        %
----|-------|----|-------|----------|----------|--------|
   1  30.351 MM    0.5251 1324.41382   42.04012   2.5196
   2  32.690 MM    0.5910 5.12408e4 1444.90955  97.4804
```

(*R*)-*N,N*-Dimethyl-2-(phenyl(*o*-tolyl)methoxy)ethan-1-amine (3)

IB-3, *n*-hexane:(*i*-PrOH:EtOH = 1:1 + 0.5% (*v*/*v*) Et_2NH) 95:5, flow rate 0.8 mL/min, 220 nm, 20 °C

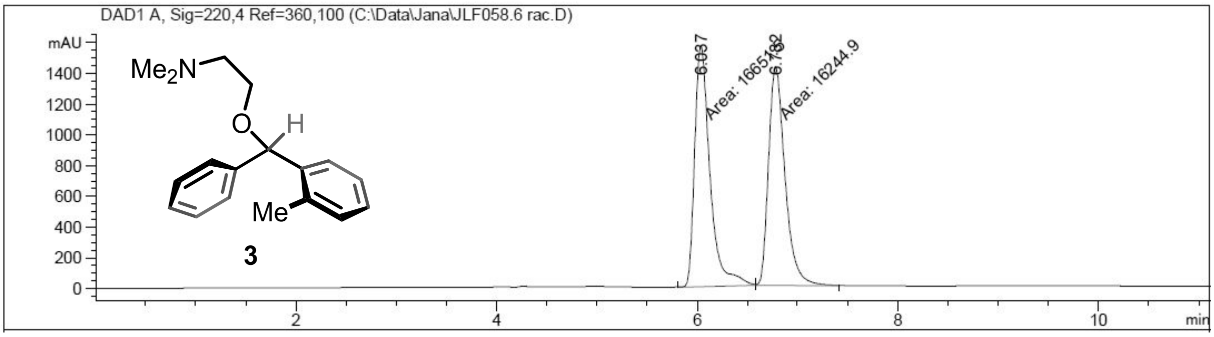

Signal 1: DAD1 A, Sig=220,4 Ref=360,100

Peak #	RetTime [min]	Type	Width [min]	Area [mAU*s]	Height [mAU]	Area %
1	6.037	MM	0.1769	1.66515e4	1568.84900	50.6179
2	6.782	MM	0.1901	1.62449e4	1423.93579	49.3821

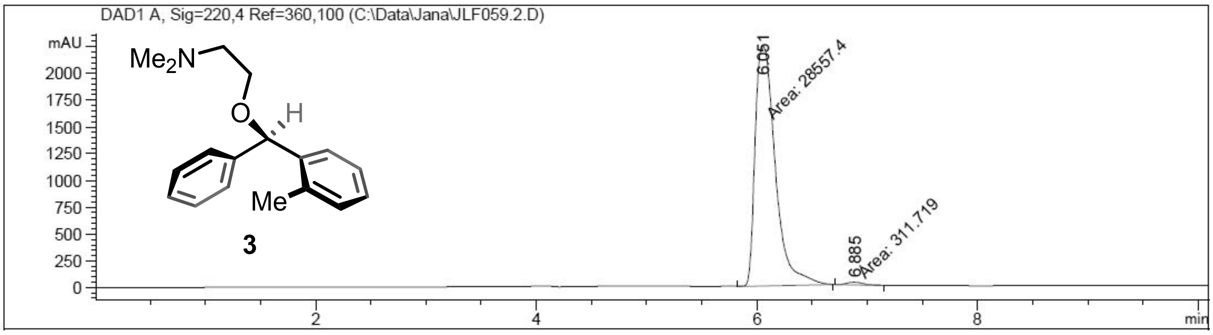

Signal 1: DAD1 A, Sig=220,4 Ref=360,100

Peak #	RetTime [min]	Type	Width [min]	Area [mAU*s]	Height [mAU]	Area %
1	6.051	MM	0.2118	2.85574e4	2247.12109	98.9202
2	6.885	MM	0.1934	311.71936	26.85960	1.0798

(*R*)-1-(2-(Phenyl(*o*-tolyl)methoxy)ethyl)piperidine (3')

OJ-3, *n*-hexane:(*i*-PrOH + 1% (*v*/*v*) Et_2NH) 99:1, flow rate 0.8 mL/min, 220 nm, 25 °C

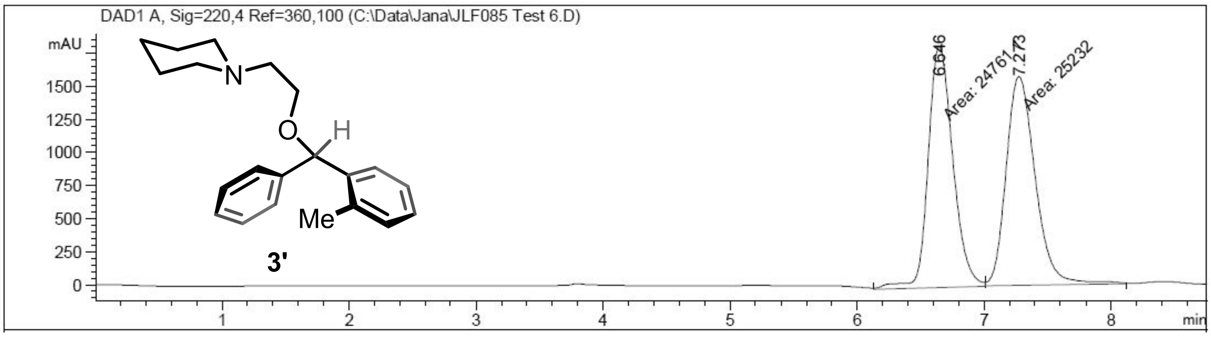

Signal 1: DAD1 A, Sig=220,4 Ref=360,100

Peak #	RetTime [min]	Type	Width [min]	Area [mAU*s]	Height [mAU]	Area %
1	6.646	MM	0.2264	2.47611e4	1822.96838	49.5290
2	7.273	MM	0.2662	2.52320e4	1579.65332	50.4710

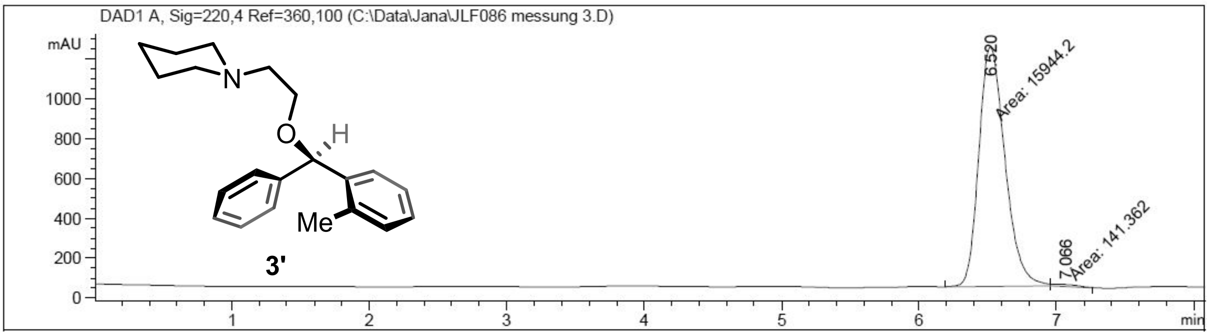

Signal 1: DAD1 A, Sig=220,4 Ref=360,100

Peak #	RetTime [min]	Type	Width [min]	Area [mAU*s]	Height [mAU]	Area %
1	6.520	MM	0.2208	1.59442e4	1203.72815	99.1212
2	7.066	MM	0.2203	141.36169	10.52314	0.8788

(*R*)-*N*,*N*-Dimethyl-2-(naphthalen-2-yl(*p*-tolyl)methoxy)ethan-1-amine (4)

OD-3, *n*-hexane:(*i*-PrOH:EtOH = 1:1 + 1% (*v*/*v*) Et_2NH) 99:1, flow rate 0.8 mL/min, 220 nm, 25 °C

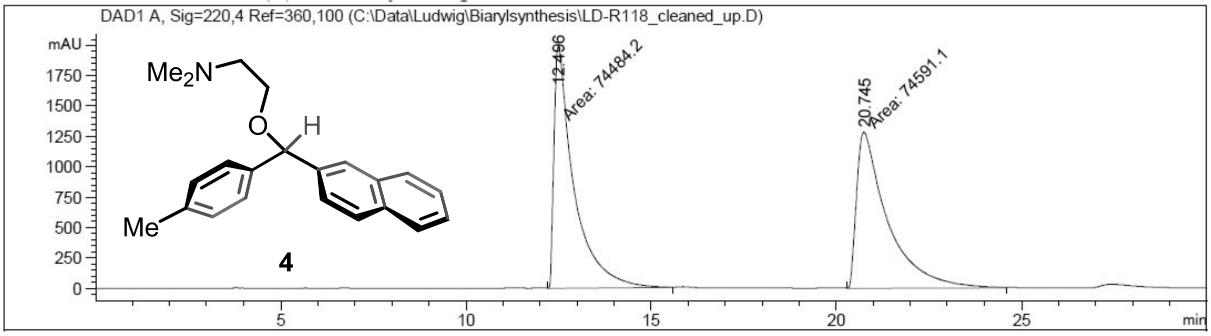

Signal 1: DAD1 A, Sig=220,4 Ref=360,100

Peak #	RetTime [min]	Type	Width [min]	Area [mAU*s]	Height [mAU]	Area %
1	12.496	MM	0.6241	7.44842e4	1989.25073	49.9641
2	20.745	MM	0.9683	7.45911e4	1283.84814	50.0359

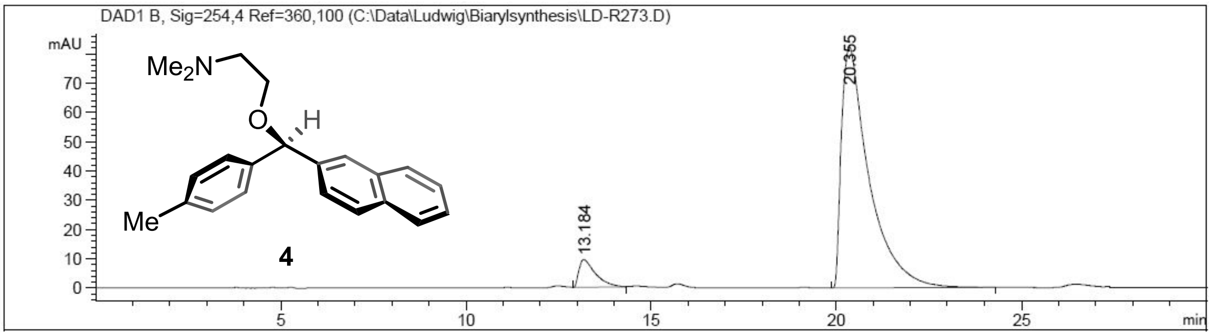

Signal 1: DAD1 B, Sig=254,4 Ref=360,100

Peak #	RetTime [min]	Type	Width [min]	Area [mAU*s]	Height [mAU]	Area %
1	13.184	BB	0.4427	290.79608	9.45936	6.3992
2	20.355	BB	0.7488	4253.49414	82.32162	93.6008

(*S*)-1-(2-((4-Chlorophenyl)(phenyl)methoxy)ethyl)piperidine (6)

OD-3, *n*-hexane:(*i*-PrOH + 1% (*v*/*v*) Et_2NH) 99:1, flow rate 0.6 mL/min, 220 nm, 25 °C

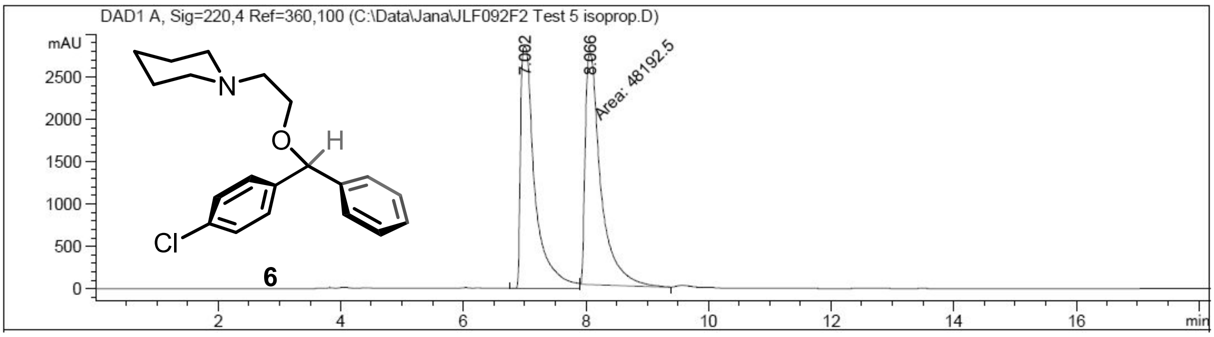

Signal 1: DAD1 A, Sig=220,4 Ref=360,100

Peak #	RetTime [min]	Type	Width [min]	Area [mAU*s]	Height [mAU]	Area %
1	7.002	BV	0.2435	4.68759e4	2856.11621	49.3076
2	8.066	MM	0.2947	4.81925e4	2725.10547	50.6924

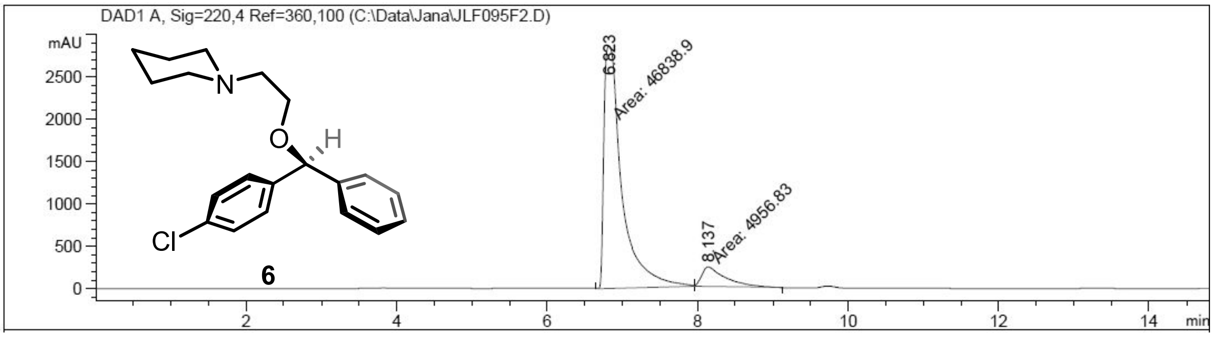

Signal 1: DAD1 A, Sig=220,4 Ref=360,100

Peak #	RetTime [min]	Type	Width [min]	Area [mAU*s]	Height [mAU]	Area %
1	6.823	MM	0.2730	4.68389e4	2859.61572	90.4300
2	8.137	MM	0.3611	4956.83301	228.78406	9.5700

(*S*)-1-Chloro-4-(chloro(phenyl)methyl)benzene (S11)

OD-3, *n*-hexane:*i*-PrOH 99.9:0.1, flow rate 0.6 mL/min, 220 nm, 25 °C

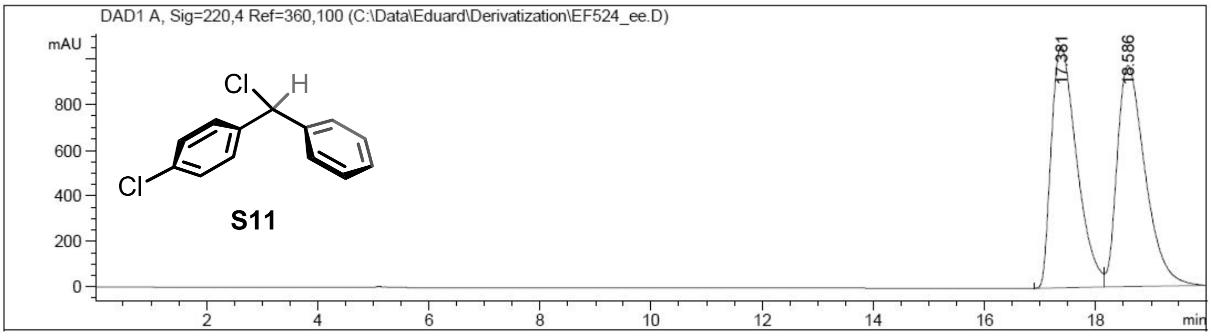

Signal 1: DAD1 A, Sig=220,4 Ref=360,100

Peak #	RetTime [min]	Type	Width [min]	Area [mAU*s]	Height [mAU]	Area %
1	17.381	BV	0.4946	3.36686e4	1059.83923	49.6918
2	18.586	VBA	0.5410	3.40862e4	972.40503	50.3082

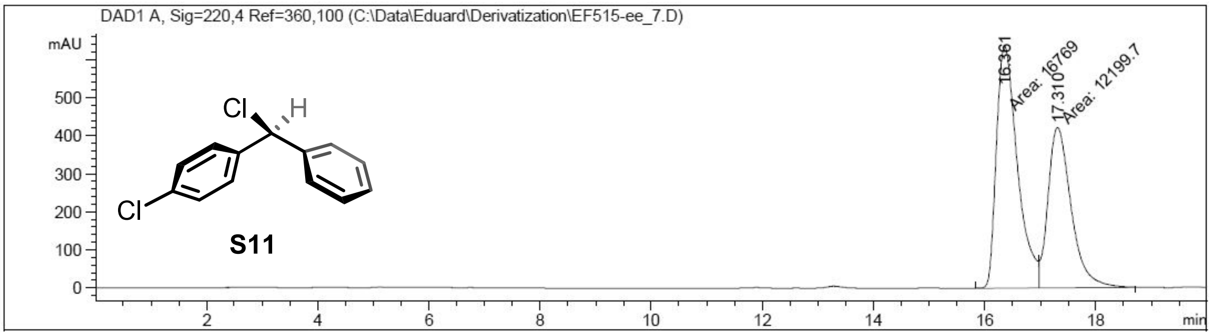

Signal 1: DAD1 A, Sig=220,4 Ref=360,100

Peak #	RetTime [min]	Type	Width [min]	Area [mAU*s]	Height [mAU]	Area %
1	16.361	MF	0.4401	1.67690e4	634.97919	57.8867
2	17.310	FM	0.4826	1.21997e4	421.31967	42.1133

(*R*)-1-((4-Chlorophenyl)(phenyl)methyl)-4-methylpiperazine (8)

OD-3, *n*-hexane:(*i*-PrOH + 1% (*v*/*v*) Et_2NH) 98:2, flow rate 0.8 mL/min, 220 nm, 25 °C

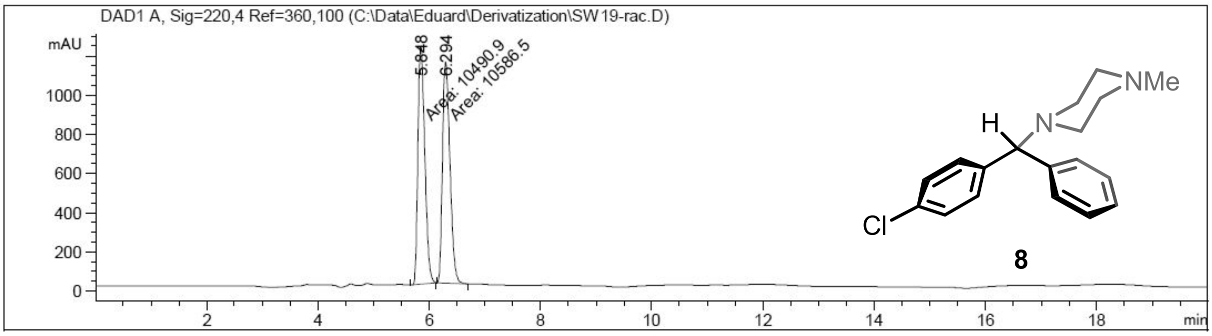

Signal 1: DAD1 A, Sig=220,4 Ref=360,100

Peak #	RetTime [min]	Type	Width [min]	Area [mAU*s]	Height [mAU]	Area %
1	5.848	MM	0.1441	1.04909e4	1213.60779	49.7732
2	6.294	MM	0.1562	1.05865e4	1129.91858	50.2268

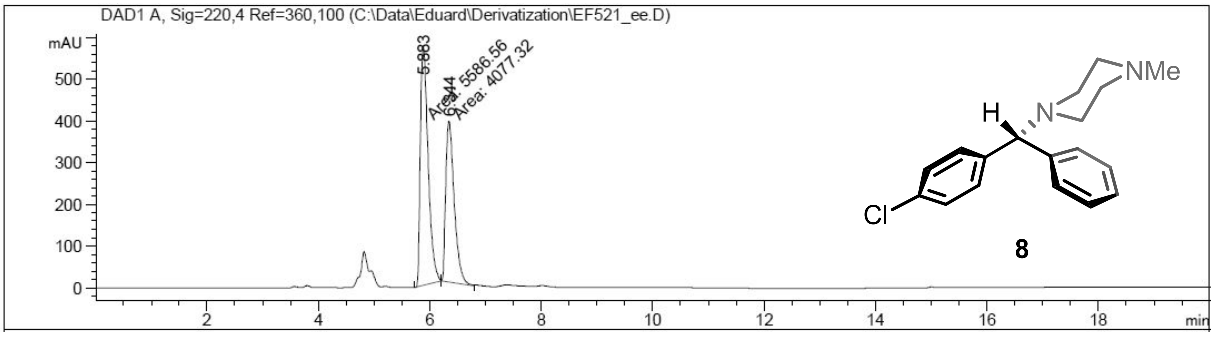

Signal 1: DAD1 A, Sig=220,4 Ref=360,100

Peak #	RetTime [min]	Type	Width [min]	Area [mAU*s]	Height [mAU]	Area %
1	5.883	MM	0.1619	5586.56006	575.12598	57.8087
2	6.344	MM	0.1758	4077.31836	386.50586	42.1913
